# Supplementary material for: Income determines the impact of cash transfers on HIV/AIDS: cohort study of 22.7 million Brazilians
Source: Nat Commun. 2024 Feb 12;15:1307. doi: 10.1038/s41467-024-44975-z (PMC10861499; doi:10.1038/s41467-024-44975-z)
Supplement: Supplementary file 1 — Supplementary Information [file 41467_2024_44975_MOESM1_ESM.pdf]

Supplementary Appendix

Income determines the impact of cash transfers on HIV/AIDS: cohort study of 22.7 million Brazilians

Silva AF; Dourado I; Lua I; Jesus GS; Guimarães NS; Morais GAS; Anderle, RVR; Pescarini JM; Machado DB; Carlos AST Santos; Ichihara MY; Barreto ML; Magno L; Souza LE; Macinko J; Rasella D

Table of contents

1.Definitions.. 3
2. Descriptive analysis by outcome.. 5
Table S1.. 5
Table S2.. 6
Table S3.. 7
Table S4.. 8
Table S5.. 9
Table S6.. 10
Table S7.. 11
Table S8.. 15
Table S9.. 17
3. Estimation of logistic regression by outcome.. 19
Table S10.. 20
4. Sensitivity Analyses.. 21
Table S11.. 22
Table S12.. 23
Table S13.. 24
Table S14.. 25
Table S15.. 26
Table S16.. 27
Table S17.. 28
Table S18.. 29
Table S19.. 30
Table S20.. 31
Table S21.. 32
5. Triangulation Analyses.. 34
Table S22.. 35
6. IPTW Poisson regression unadjusted.. 37
Table S23.. 37
7. Complementary analyzes.. 38
Table S24.. 38
Table S25.. 39
Table S26.. 40
Table S27.. 41
Table S28.. 43
Table S29.. 44
Table S30.. 45
Table S31.. 46
Table S32.. 47
8. IPTW Poisson regression stratified models.. 48
Table S33.. 49
Table S34.. 51
Table S35.. 53
Table S36.. 55
Table S37.. 57
References.. 59

1. Definitions

The *Cadastro Único* (CadUnico) database and the 100 Million Brazilian Cohort

The 100 Million Brazilian Cohort was based on the baseline information of families, during the period from January 1, 2001 to December 31, 2017, who sought to benefit from the Brazilian government's social programs through registration in the Unified Registry for Social Programs (in Portuguese: *Cadastro Único para Programas Sociais* – CadUnico). The CadUnico is an administrative database, to which Brazilians aged 16 or over can apply by registering their personal information (age, sex, race/ethnicity, education and others) and household information (household density, familiar income, structural characteristics of the residence and others), as long as they are within one of these categories: (i) belong to a family with a monthly per capita income of up to half a minimum wage; (ii) belong to a family with a total monthly income of up to three minimum wages; (iii) belong to a family with an income greater than three minimum wages, provided that the registration is linked to inclusion in social programs in the three spheres of government; (iv) be the only resident of the household, or; (v) living on the streets (alone or with the family).

Upon registration, individuals receive a unique identifier code and are searched for socioeconomic characteristics. At the end of 2017, CadÚnico had approximately 114 million individuals on its register, which represents around 50% of the Brazilian population. It is a social tool that identifies and characterizes especially low-income families, allowing the government to know the socioeconomic aspect of the poorest and use it for the selection of social programs.[1,2]

In addition to the information from CadÚnico, the Cohort is also composed of health-related databases. For our study, the information used to identify morbidity and mortality from AIDS comes from the National System of Disease Notification (in Portuguese: *Sistema de Informação de Agravos de Notificação - SINAN*) and the Mortality Information System (in Portuguese: *Sistema de Informações Sobre Mortalidade - SIM*).[3] Created by the Center for Integration of Data and Knowledge for Health (CIDACS / FIOCRUZ)[2], the Cohort aims to facilitate research and continuous assessment of social determinants and the effects of social policies and programs in health contexts in Brazil. It has 246 variables with demographic and socioeconomic information at the individual and family level. The codes and linking algorithms between the databases were built to make efficient and specific links through five identifiers: the date of birth, the municipality of residence, the sex, the name and the mother's name of each individual presented in each of the databases[2,4,5]. The linkage was performed at the individual level in two stages using the CIDACS-RL (https://gitHub.com/gcgbarbosa/cidacs-rl). First, the inputs were linked deterministically. In the second step, for cases that were not deterministically linked, they were linked based on a similarity score for all pairwise comparisons, ranging from 0 to 1. The entries with the highest similarity scores were considered as linked pairs. The quality of each link for all causes between CadUnico, SINAN e SIM has been extensively evaluated and validated.[6,7]

The variables at the municipal level of the resident individual's baseline were linked to the CadÚnico records by the municipality code and the year of information. The average variables for the period that capture the level of health surveillance and the risk of AIDS endemicity were linked only by the municipal identifier.

The *Programa Bolsa Família* (PBF)

This conditional cash tranfer (CCT) is a direct income transfer program for families living in poverty and extreme poverty in Brazil. Created in 2003, the program was originated from the unification of Other four government social protection policies (In portuguese: *Bolsa Escola* (2001), *Bolsa Alimentação* (2001), *Auxílio-Gás* (2002) e *Fome-Zero* (2003)).[8] The program's objectives are: to fight against hunger and to promote nutritional and food security; alleviate poverty and other forms of deprivation; and toprovide easy access to public health, education, social assistance. The PBF seeks to ensure that these families are able to get out of poverty and vulnerability conditions in the short-long term. The target beneficiaries of the program are made up of poor or extremely poor families. Extremely poor families are considered to have a monthly *per capita* income of up to US$12 (BRL 60) in 2007-2008 period, up to US$14 (BRL 70) from 2009 to 2013, and up to US$15.4 (BRL 77) in 2014 and 2015. Poor families are those with a *per capita* income between US$12 (BRL 60.01) and US$24 (BRL 120) in 2007 and 2008, between US$14 (BRL 70.01) and US$28 (BRL 140) from 2009 to 2013, and between US$15.4 (BRL 77.01) and US$30.8 (BRL 154) in 2014 and 2015. Poor families are allowed to participate in the program as long as they include in the registry children or adolescents between 0 and 17 years old, or pregnant women of any age. The registration on the CadUnico is a prerequisite for receiving the PBF (or any other federal social protection program), but it does not imply approval or immediate receipt. After registering with CadUnico and fulfilling the aforementioned requirements, the Ministry of Citizenship (in Portuguese: *Ministério da Cidadania*) elects the beneficiaries of the program by following the previously described criteria. Once the income transfer is approved, there are conditions for its maintenance according to each family composition. For pregnant women, for instance, the attendance to prenatal appointments is required. For mothers who breastfeed, they must participate in educational activities provided by the Ministry of Health – MoH (In Portguese: *Ministério da Saúde*). For children aged from 0 to 7, their parents must follow the vaccination schecule of them. In the case of women aged 14 to 44 years, they must be monitored by the health team. Finally, is required the guarantee of minimum school attendance of 85% for children and 75% for teenagers. The monthly amount received per family is the sum of the types of benefits provided by the PBF. There are two types, the first one is the basic benefit referring to extreme poor only (amount of US$17.8; BRL 89) and the second is the variable benefit, in which poor families can accumulate up to 5 benefits, depending on the number of breastfeeding mothers, pregnant women, or the number of children and adolescents from 0 to 16 years old (amount varies between US$8.2 and US$9.6; BRL 41 and BRL 48, respectively). Families covered by the PBF are required to update their information every two years. The Ministry of Citizenship periodically carries out an inspection of the suitable profiles for the maintenance of the benefit.

Criteria for classifying the exposition status of the individuals under study

In our study the beneficiary group was defined as eligible individuals who received PBF benefits within the required administrative period, and their exposure started with receipt of the benefit until the end of their cohort follow-up. While the majority of them was receiving the benefits until the end of the cohort follow-up, some individuals ended their receipt before, but still we considered the end of the cohort follow-up as end of their exposure period. This was because individuals that improve their economic conditions -and are not eligible anymore for PBF- should exit the PBF, but we considered this period of improved economic situation and its reduced probability of AIDS incidence and mortality as a long-term PBF effect.

For additional analysis (see Table S29), we created a new group of PBF beneficiaries, with the cohort ending on the date of the termination of cash transfer receipt. The identified effect follows the same direction and intensity as the main model.

The non-beneficiary group was defined as individuals who had never benefited from the program throughout their follow-up period. In cases where an eligible individual did not receive the PBF benefit at all or did not receive it within the required administrative period following the enrollment on Unified Registry (6 months),[9] it was classified in the non-beneficiary group and, in case the benefits arrived later, the start date of PBF receipt was considered the end of his follow-up period as non-beneficiary.

2. Descriptive analysis by outcome

The number of observations per AIDS cases, AIDS deaths and AIDS deaths/AIDS cases are presented in Table S1. Descriptive analyses related to AIDS outcome (incidence, mortality, and case-fatality rate) by variables are presented on the Tables S2, S3 and S4. The number of observations of AIDS cases, AIDS deaths and AIDS deaths/AIDS cases by wealth quartile are shown in Table S5. The incidence, mortality, and case-fatality rates (calculated per person-year) by wealth, sex and age are shown in Table S6. The stratified descriptive statistics are displayed in the Tables S7, S8, and S9. All the information in these Tables is divided into two groups, PBF beneficiaries and non-beneficiaries. The analyses stratified by quartile of wealth were built according to measured by capita expenses proportional to the baseline minimum wage (MW): quartile 1: 0% a 0.1%; quartile 2: 0.1%< a 18.5%; quartile 3: 18.5% < a 56.5%; quartile 4: 56.5%< (Table S7). The descriptive statistics by sex (female and male) are presented in Table S8. Finally, the analyses by age were stratified into adolescents and youth, adults, and older people in Table S9.

Table S1. Number of observations per AIDS case, death from AIDS and death from AIDS/AIDS case by *Programa Bolsa Família* (PBF) beneficiaries (BF) and non-beneficiaries (N-BF).

Variables	*Programa Bolsa Familia* (PBF)		Total
	N-BF	BF	
**AIDS cases[a]**			
**No**	15,565,165	7,201,620	22,766,785
**Yes**	13,011	9,201	22,212
**Total**	15,578,176	7,210,821	22,788,997
**AIDS deaths[b]**			
**No**	15,573,758	7,207,589	22,781,347
**Yes**	4,418	3,232	7,650
**Total**	15,578,176	7,210,821	22,788,997
**AIDS deaths/AIDS cases [a,b]**			
**No**	5,367	5,973	11,340
**Yes**	1,379	1,629	3,008
**Total**	6,746	7,602	14,348

Notes: [a] New AIDS cases, defined by adapted CDC criteria, the Rio de Janeiro/Caracas criteria; [b] AIDS deaths, considering as underlying cause the ICD-10 codes B20 to B24.

Table S2. Descriptive analyses of AIDS cases of *Programa Bolsa Família* (PBF) beneficiaries (BF) and non-beneficiaries (N-BF) (n = 22,212), 2007-2015.

Social and Demographic Variables	N-BF (n = 13,011)		BF (n= 9,201)		P-value[a]	SMD[b]
	No. or Mean	(%) or CI	No. or Mean	(%) or CI		
**Sex**					<0.001	0.2679
Female	6,030	46.3	5,484	59.6		
Male	6,981	53.7	3,717	40.4		
**Age**					<0.001	0.1880
Adolescents and Youth people[c]	2,237	17.2	1,699	18.5		
Adults[d]	10,428	80.2	7,467	81.2		
Older people[e]	345	2.7	35	0.4		
**Race/ethnicity**					<0.001	0.1343
White	4,356	35.1	2.717	30.5		
Midex-race	6,531	52.7	4.756	53.4		
Black	1,485	12.0	1.379	15.5		
Indigenous	32	0.3	51	0.6		
**Education**					<0.001	0.1834
More than High School	271	2.3	63	0.8		
High school	2,389	20.6	1,392	16.7		
Elementary school	7,720	66.6	6,128	73.5		
Attended pre-school	79	0.7	49	0.6		
Never attended school	1,135	9.8	703	8.4		
**Wealth[f]**					<0.001	0.4545
Level 1 (More wealth)	787	6.1	111	1.2		
Level 2	1,802	13.9	419	4.6		
Level 3	1,411	10.8	845	9.2		
Level 4	4,554	35.0	4,190	45.5		
Level 5 (Lower wealth)	4,453	34.2	3,634	39.5		
**Water supply**					<0.001	0.0835
Public network	10,456	82.8	7,032	79.5		
Others[g]	2,178	17.2	1,814	20.5		
**Housing material (brick)**					<0.001	0.0190
Yes	9,973	78.9	7,052	79.7		
No[h]	2,661	21.1	1,795	20.3		
**Lighting**					<0.001	0.2016
Electricity	11,014	87.2	7,052	79.7		
Non-electric[i]	1,620	12.8	1,794	20.3		
**Region**					<0.001	0.1918
North	1,214	9.3	852	9.3		
Northeast	3,512	27.0	2,507	27.2		
Southeast	4,187	32.2	3,642	39.6		
South	3,028	23.3	1,667	18.1		
Central-west	1,068	8.2	533	5.8		
**Area of residence**					<0.001	0.0364
Rural	1,128	8.7	698	7.7		
Urban	11,788	91.3	8,331	92.3		
**Average AIDS incidence rate[j]**	28.49	(28.1-28.8)	31.82	(31.3-32.2)	<0.001	-0.1635
**Inadequate sanitation[l]**	8.98	(8.79-9.16)	8.27	(8.09-8.45)	<0.001	0.0721
**Unemployment rate (%)[m]**	8.76	(8.69-8.82)	9.34	(9.26-9.42)	<0.001	-0.1523
**Doctors per 1000 inhabitants[n]**	1.58	(1.55-1.60)	1.73	(1.71-1.76)	<0.001	-0.1328
**Nurses per 1,000 inhabitants[n]**	0.65	(0.65-0.66)	0.68	(0.67-0.69)	<0.001	-0.0709
**Hospital beds per 1,000 inhabitants[n]**	2.56	(2.53-2.59)	2.64	(2.60-2.67)	<0.001	-0.0481
**Year of entry of into the cohort**					<0.001	0.4084
2007	3,750	28.8	3,309	25.1		
2008	2,316	17.8	1,360	14.8		
2009	1,036	8.0	1,807	19.6		
2010	1,539	11.8	1,331	14.5		
2011	1,046	8.0	894	9.7		
2012	1,724	13.3	757	8.2		
2013	888	6.8	453	4.9		
2014	580	4.5	235	2.6		
2015	132	1.0	55	0.6		

Notes: [a] The following were used for a comparison between the groups: (i) the two-tailed t-test for continuous variables and (ii) the Pearson's chi-squared test (χ^2) for categorical variables. [b] SMD - Standardized Mean Difference. [c] Aged between 13 and 24; [d] Aged between 25 and 64; [e] Aged 65 or older. [f] Measured by capita expenses proportional to the baseline minimum wage (MW). Level 1 (More wealth): "1 or more". Level 2: "0.5 to 1". Level 3: "0.25 to 0.49". Level 4: "0< to 0.24". Level 5 (Lower wealth): "Nothing declared". [g] Water supply: Others – well, spring and others. [h] Housing Material: No – Coated clay, uncoated clay, wood, and others. [i] Lighting: Non-electric – No meter, lamp, candle and others. [j] Average rates for the period (2007-2015) by municipality. [l] % of the municipality's population with inadequate baseline sanitation. [m] Baseline municipality unemployment rate. [n] Per 1,000 inhabitants of the baseline municipality.

Table S3. Descriptive analyses of AIDS deaths of *Programa Bolsa Família* (PBF) beneficiaries (BF) and non-beneficiaries (N-BF) (n = 7,650), 2007-2015.

Social and Demographic Variables	N-BF (n = 4,418)		BF (n= 3,232)		P-value[a]	SMD[b]
	No. or Mean	(%) or CI	No. or Mean	(%) or CI		
**Sex**					<0.001	0.2528
Female	1,946	44.0	1,829	56.6		
Male	2,472	56.0	1,403	43.4		
**Age**					<0.001	0.2237
Adolescents and Youth people[c]	439	9.9	378	11.7		
Adults[d]	3,799	86.0	2,830	87.6		
Older people[e]	180	4.1	24	0.7		
**Race/ethnicity**					<0.001	0.1638
White	1,497	35.2	936	29.8		
Mixed-race	2,171	51.0	1,624	51.7		
Black	584	13.7	559	17.8		
Indigenous	5	0.1	20	0.6		
**Education**					<0.001	0.1505
More than High School	55	1.4	8	0.3		
High school	587	14.6	378	12.7		
Elementary school	2,869	71.3	2,245	75.7		
Attended pre-school	19	0.5	19	0.6		
Never attended school	494	12.3	317	10.7		
**Wealth[f]**					<0.001	0.4486
Level 1 (More wealth)	243	5.5	40	1.2		
Level 2	592	13.4	132	4.1		
Level 3	843	10.9	278	8.6		
Level 4	1,555	35.2	1,430	44.3		
Level 5 (Lower wealth)	1,544	35.0	1,351	41.8		
**Water supply**					<0.001	0.0745
Public network	3,525	82.3	2,483	79.3		
Others[g]	760	17.7	647	20.7		
**Housing material (Brick)**					<0.001	0.0245
Yes	3,390	79.1	2,508	80.1		
No[h]	895	20.9	623	19.9		
**Lighting**					<0.001	0.1857
Electricity	3,683	86.0	2,470	78.9		
Non-electric[i]	602	14.0	660	21.1		
**Region**					<0.001	0.2509
North	361	8.2	268	8.3		
Northeast	1,025	23.2	744	23.0		
Southeast	1,721	39.0	1,579	1,579		
South	986	22.3	469	469		
Central-west	325	7.4	172	172		
**Area of residence**					<0.001	0.0439
Rural	360	8.2	224	7.0		
Urban	4,033	91.8	2,963	93.0		
**Average AIDS mortality rate[j]**	8.79	(8.60-8.97)	9.50	(9.30-9.70)	<0.001	-0.1171
**Inadequate sanitation[l]**	8.81	(8.51-9.12)	8.18	(7.88-8.12)	<0.001	0.0659
**Unemployment rate (%)[m]**	8.84	(8.73-8.95)	9.48	(9.35-9.60)	<0.001	-0.1713
**Doctors per 1000 inhabitants[n]**	1.60	(1.57-1.64)	1.73	(1.69-1.77)	<0.001	-0.1108
**Nurses per 1,000 inhabitants[n]**	0.65	(0.64-0.66)	0.67	(0.66-0.68)	<0.001	-0.0423
**Hospital beds per 1,000 inhabitants[n]**	2.58	(2.53-2.63)	2.65	(2.59-2.70)	<0.001	-0.0410
**Year of entry of into the cohort**					<0.001	0.3898
2007	1,262	28.6	892	27.6		
2008	827	18.7	532	16.5		
2009	359	8.1	609	18.8		
2010	511	11.6	424	13.1		
2011	360	8.1	290	9.0		
2012	612	13.9	237	7.3		
2013	256	5.8	153	4.7		
2014	184	4.2	73	2.3		
2015	47	1.1	22	0.7		

Notes: [a] The following were used for a comparison between the groups: (i) the two-tailed t-test for continuous variables and (ii) the Pearson's chi-squared test (χ^2) for categorical variables. [b] SMD - Standardized Mean Difference. [c] Aged between 13 and 24. [d] Aged between 25 and 64. [e] Aged 65 or older. [f] Measured by capita expenses proportional to the baseline minimum wage (MW). Level 1 (More wealth): "1 or more". Level 2: "0.5 to 1". Level 3: "0.25 to 0.49". Level 4: "0< to 0.24". Level 5 (Lower wealth): "Nothing declared". [g] Water supply: Others – well, spring and others. [h] Housing Material: No – Coated clay, uncoated clay, wood, and others. [i] Lighting: Non-electric – No meter, lamp, candle and others. [j] Average rates for the period (2007-2015) by municipality. [l] % of the municipality's population with inadequate baseline sanitation. [m] Baseline municipality unemployment rate. [n] Per 1,000 inhabitants of the baseline municipality. All statistical tests used where two-sided.

Table S4. Descriptive analyses of AIDS deaths/AIDS cases (case-fatality) *of Programa Bolsa Família* (PBF) beneficiaries (BF) and non-beneficiaries (N-BF) (n = 3,008), 2007-2015.

Social and Demographic Variables	N-BF (n = 1,379)		BF (n= 1,629)		P-value[a]	SMD[b]
	No. or Mean	(%) or CI	No. or Mean	(%) or CI		
**Sex**					<0.001	0.3887
Female	503	35.5	904	55.5		
Male	876	63.5	725	44.5		
**Age**					<0.001	0.2964
Adolescents and Youth people[c]	116	8.4	203	12.5		
Adults[d]	1,189	86.2	1,414	86.8		
Older people[e]	74	5.4	12	0.7		
**Race/ethnicity**					<0.001	0.2364
White	453	34.6	442	28.0		
Mixed-race	712	54.3	848	53.7		
Black	145	11.1	276	17.5		
Indigenous	1	0.1	13	0.8		
**Education**					<0.001	0.2164
More than High School	24	1.9	3	0.2		
High school	206	16.4	201	13.5		
Elementary school	853	68.0	1,114	74.8		
Attended pre-school	4	0.3	9	0.6		
Never attended school	167	13.3	163	10.9		
**Wealth[f]**					<0.001	0.5965
Level 1 (More wealth)	81	5.9	21	1.3		
Level 2	259	18.8	67	4.1		
Level 3	177	12.8	156	9.6		
Level 4	469	34.0	715	43.9		
Level 5 (Lower wealth)	393	28.5	669	41.1		
**Water supply**					<0.001	0.1183
Public network	1,121	83.9	1,255	79.3		
Others[g]	215	16.1	327	20.7		
**Housing material (Brick)**					<0.001	0.0530
Yes	1,075	80.5	1,239	78.3		
No[h]	261	19.5	343	21.7		
**Lighting**					<0.001	0.2100
Electricity	1,150	86.1	1,235	78.1		
Non-electric[i]	186	13.9	347	21.9		
**Region**					<0.001	0.2097
North	115	8.3	137	8.4		
Northeast	357	25.9	447	27.4		
Southeast	481	34.9	685	42.1		
South	292	21.2	249	15.3		
Central-west	134	9.7	111	6.8		
**Area of residence**					<0.001	0.2097
Rural	105	7.7	121	7.5		
Urban	1,267	92.3	1,491	92.5		
**Average AIDS case-fatality rate[j]**	30.88	(20.8-30.8)	30.86	(30.8-30.8)	<0.001	-0.0671
**Inadequate sanitation[l]**	7.56	(7.33-7.78)	8.33	(8.13-8.54)	<0.001	-0.0386
**Unemployment rate (%)[m]**	8.01	(7.92-8.10)	9.33	(9.24-9.42)	<0.001	-0.3057
**Doctors per 1000 inhabitants[n]**	1.69	(1.66-1.72)	1.72	(1.70-1.75)	<0.001	-0.0167
**Nurses per 1,000 inhabitants[n]**	0.71	(0.70-0.72)	0.69	(0.68-0.69)	<0.001	0.0807
**Hospital beds per 1,000 inhabitants[n]**	2.57	(2.53-2.61)	2.63	(2.60-2.67)	<0.001	-0.2508
**Year of entry of into the cohort**					<0.001	0.5025
2007	310	22.5	450	27.6		
2008	203	14.7	261	16.0		
2009	118	8.6	308	18.9		
2010	174	12.6	229	14.1		
2011	130	9.4	150	9.2		
2012	229	16.6	123	7.6		
2013	108	7.8	68	4.2		
2014	89	6.5	36	2.2		
2015	18	1.3	4	0.2		

Notes: [a] The following were used for a comparison between the groups: (i) the two-tailed t-test for continuous variables and (ii) the Pearson's chi-squared test (χ^2) for categorical variables. [b] SMD - Standardized Mean Difference. [c] Aged between 13 and 24. [d] Aged between 25 and 64. [e] Aged 65 or older. [f] Measured by capita expenses proportional to the baseline minimum wage (MW). Level 1 (More wealth): "1 or more". Level 2: "0.5 to 1". Level 3: "0.25 to 0.49". Level 4: "0< to 0.24". Level 5 (Lower wealth): "Nothing declared". [g] Water supply: Others – well, spring and others. [h] Housing Material: No – Coated clay, uncoated clay, wood, and others. [i] Lighting: Non-electric – No meter, lamp, candle and others. [j] Average rates for the period (2007-2015) by municipality. [l] % of the municipality's population with inadequate baseline sanitation. [m] Baseline municipality unemployment rate. [n] Per 1,000 inhabitants of the baseline municipality. All statistical tests used where two-sided.

Table S5. Number of observations per AIDS case, death from AIDS and death from AIDS/AIDS case by *Programa Bolsa Família* (PBF) beneficiaries (BF) and non-beneficiaries (N-BF), stratified by wealth quartile.

Variables	Wealth[a]				Total
	Quartile 1 [b]	Quartile 2 [c]	Quartile 3 [d]	Quartile 4 [e]	
**AIDS cases [f]**					
		**Bolsa Familia = 0**			
**No**	3,914,812	2,643,379	3,927,723	5,079,251	15,565,165
**Yes**	5,595	3,014	2,107	2,295	13,011
**Total**	3,920,407	2,646,393	3,929,830	5,081,546	15,578,176
		**Bolsa Familia = 1**			
**No**	2,858,820	1,959,281	1,768,297	615,222	7,201,620
**Yes**	5,109	2,299	1,365	428	9,201
**Total**	2,863,929	1,961,580	1,769,662	615,650	7,210,821
		**General**			
**No**	6,773,632	4,602,660	5,696,020	5,694,473	22,766,785
**Yes**	10,704	5,313	3,472	2,723	22,212
**Total**	6,784,336	4,607,973	5,699,492	5,697,196	22,788,997
**AIDS deaths [g]**					
		**Bolsa Familia = 0**			
**No**	3,918,515	2,645,320	3,929,119	5,080,804	15,573,758
**Yes**	1,892	1,073	711	742	4,418
**Total**	3,920,407	2,646,393	3,929,830	5,081,546	15,578,176
		**Bolsa Familia = 1**			
**No**	2,862,125	1,960,766	1,769,192	615,506	7,207,589
**Yes**	1,804	814	470	144	3,232
**Total**	2,863,929	1,961,580	1,769,662	615,650	7,201,821
		**General**			
**No**	6,780,640	4,606,086	5,698,311	5,696,310	22,781,347
**Yes**	3,696	1,887	1,181	886	7,650
**Total**	6,784,336	4,607,973	5,699,492	5,697,196	22,788,997
**AIDS deaths/AIDS cases [f,g]**					
		**Bolsa Familia = 0**			
**No**	1,557	1,211	1,133	1,466	5,367
**Yes**	461	352	263	303	1,379
**Total**	2,018	1,563	1,396	1,769	6,746
		**Bolsa Familia = 1**			
**No**	3,307	1,485	895	286	5,973
**Yes**	907	404	245	73	1,629
**Total**	4,214	1,889	1,140	359	7,602
		**Geral**			
**No**	4,864	2,696	2,028	1,752	11,340
**Yes**	1,368	756	508	376	3,008
**Total**	6,232	3,452	2,536	2,128	14,348

Notes: [a] Measured by capita expenses proportional to the baseline minimum wage (MW). [b] Quartile 1: 0% a 0.1%. [c] Quartile 2: 0.1%< a 18.5%. [d] Quartile 3: 18.5% < a 56.5%. [e] Quartile 4: 56.5%<. [f] New AIDS cases, defined by adapted CDC criteria, the Rio de Janeiro/Caracas criteria; [g] AIDS deaths, considering as underlying cause the ICD-10 codes B20 to B24.

Table S6. AIDS incidence, mortality and case-fatality rates by wealth, sex, and age subgroups [by *Bolsa Família Program* (PBF) beneficiaries (BF) and non-beneficiaries (N-BF)].

Wealth[a]	Incidence Rate[i]			Mortality Rate[i]			Case-Fatality Rate[i]		
	**Total**	**N-BF**	**BF**	**Total**	**N-BF**	**BF**	**Total**	**N-BF**	**BF**
**Quartile 1** [b]	32.83	43.74	25.79	11.33	14.79	9.09	7.09	9.15	6.35
**Quartile 2** [c]	28.47	32.01	24.86	10.11	11.39	8.80	7.76	8.43	7.24
**Quartile 3** [d]	22.83	22.87	22.76	7.77	7.72	7.85	10.1	10.80	9.46
**Quartile 4** [e]	18.98	18.48	22.25	6.17	5.97	7.49	9.89	9.60	8.20
**Sex**									
**Female**	24.59	25.35	23.80	8.06	8.18	7.93	6.63	8.22	5.97
**Male**	31.47	34.82	26.57	11.40	12.32	10.06	9.43	10.11	8.71
**Age**									
**Adolescents and young**[f]	17.33	21.93	13.58	3.59	4.30	3.02	4.60	4.97	4.40
**Adults**[g]	35.07	38.61	31.09	12.99	14.06	11.78	8.51	9.95	7.57
**Older people**[h]	5.37	5.21	7.74	2.88	2.72	5.30	17.10	16.29	28.64

Notes: [a] Measured by capita expenses proportional to the baseline minimum wage (MW). [b] Quartile 1: 0% a 0.1%. [c] Quartile 2: 0.1%< a 18.5%. [d] Quartile 3: 18.5% < a 56.5%. [e] Quartile 4: 56.5%<. [f] Aged between 13 and 24. [g] Aged between 25 and 64. [h] Aged 65 or older. [i] Calculated by person-years.

Table S7. Descriptive analyses of *Programa Bolsa Família* (PBF) beneficiaries (BF) and non-beneficiaries (N-BF), 2007-2015, by subpopulation of wealth[l].

(to be continued)

Social and Demographic Variables	Quartile 1 [m] (n=6,784,336)				Quartile 2 [n] (n=4,607,973)			
	N-BF (n =3,920,407)		BF (n=2,863,929)		N-BF (n =2,646,393)		BF (n=1,961,580)	
	No. or Mean	(%) or CI	No. or Mean	(%) or CI	No. or Mean	(%) or CI	No. or Mean	(%) or CI
**Sex**								
Female	2,058,99	52.5	1,736,040	60.6	1,593,765	60.2	1,333,162	68.0
Male	1,861,408	47.5	1,127,889	39.4	1,052,628	39.8	628,418	32.0
**Age**								
Adolescents and Youth people[a]	1,407,964	35.9	1,038.778	36.3	648,668	34.4	610,713	31.1
Adults[b]	2,304,993	58.8	1,793,629	62.6	1,685,256	63.7	1,329,574	67.8
Older people[c]	207,306	5.3	31,418	1.1	312,359	11.8	21,200	1.1
**Race/ethnicity**								
White	1,020,162	27.1	707,534	25.2	882,647	34.4	572,765	30.4
Mixed-race	2,443,843	64.8	1,771,368	63.2	1,479,821	57.6	1,114,458	59.2
Black	296,327	7.9	268,470	9.6	198,277	7.7	169,545	9.0
Indigenous	10,882	0.3	56,388	2.0	7,029	0.3	24,681	1.3
**Education**								
More than High School	30,363	0.9	13,440	0.6	44,211	1.9	14,661	0.8
High school	504,021	15.6	373,860	15.3	552,528	23.7	552,192	30.5
Elementary school	2,206,589	68.2	1,769,387	72.4	1,416,945	60.8	1,107,929	61.2
Attended pre-school	32,789	1.0	19,952	0.8	26,769	1.1	13,226	0.7
Never attended school	462,103	14.3	266,239	10.9	289,188	12.4	121,328	6.7
**Water supply**								
Public network	2,605,589	68.4	1,907,469	67.9	1,947,091	75.8	1,388,041	73.3
Others[d]	1,206,031	31.6	900,345	32.1	622,648	24.2	505,220	26.7
**Housing material (Brick)**								
Yes	2,786,440	73.1	2,126,740	75.7	2,109,689	82.1	1,546,017	81.7
No [e]	1,025,015	26.9	680,965	24.3	460,040	17.9	347,248	18.3
**Lighting**								
Electricity	3,241,399	85.0	2,276,553	81.1	2,298,871	89.5	1,567,631	82.8
Non-electric [f]	570,165	15.0	531,224	18.9	270,864	10.5	325,630	17.2
**Region**								
North	549,044	14.0	433,619	15.1	301,483	11.4	263,273	13.4
Northeast	1,728,468	44.1	1,137,253	39.7	921,657	34.8	591,530	30.2
Southeast	1,023,413	26.1	940,729	32.8	887,940	33.6	834,717	42.6
South	361,356	9.2	179,565	6.3	310,881	11.7	148,192	7.6
Central-west	257,750	6.6	172,727	6.0	224,428	8.5	123,868	6.3
**Area of residence**								
Rural	1,094,140	28.2	661,238	23.3	519,309	19.6	366,299	18.7
Urban	2,789,896	71.8	2,180,502	76.7	2,125,800	80.4	1,591,746	81.3
**Average AIDS incidence rate[g]**	15.75	(12.6-15.7)	20.23	(13.9-20.2)	18.84	(18.8-18.8)	21.61	(21.5-21.6)
**Average AIDS mortality rate[g]**	4.61	(3.95-4.61)	5.95	(4.32-5.95)	5.50	(5.50-5.51)	6.46	(6.45-6.47)
**Average AIDS case-fatality rate[g]**	30.84	(9.33-30.8)	30.65	(7.95-30.6)	30.61	(30.5-30.6)	30.96	(30.9-30.9)
**Inadequate sanitation [h]**	16.72	(15.5-16.7)	14.18	(14.8-14.1)	11.57	(11.5-11.6)	10.69	(10.6-10.7)

**Unemployment rate (%)[i]**	9.64	(3.79-9.63)	10.0	(3.80-9.99)	8.54	(8.53-8.54)	8.32	(8.31-8.32)
**Doctors per 1000 inhabitants[j]**	0.94	(0.87-0.94)	1.20	(0.95-1.20)	1.25	(1.25-1.25)	1.45	(1.45-1.45)
**Nurses per 1000 inhabitants[j]**	0.46	(0.26-0.460	0.52	(0.29-0.52)	0.60	(0.60-0.60)	0.68	(0.67-0.68)
**Hospital beds per 1000 inhabitants[j]**	2.24	(1.76-2.24)	2.34	(1.62-2.34)	2.31	(2.31-2.31)	2.28	(2.28-2.29)
**Year of entry into the cohort**								
2007	1,768,370	45.1	980,479	34.2	510,538	19.3	205,097	10.5
2008	896,667	22.9	479,413	16.7	479,296	18.1	160,145	8.2
2009	451,876	11.5	775,699	27.1	358,652	13.6	298,508	15.2
2010	574,907	14.7	455,849	15.9	384,856	14.5	255,025	13.0
2011	68,870	1.8	62,373	2.2	107,594	4.1	268,664	13.7
2012	57,384	1.5	30,506	1.1	305,187	11.5	270,153	13.8
2013	35,738	0.9	29,677	1.0	196,686	7.4	241,847	12.3
2014	43,958	1.1	26,885	0.9	210,773	8.0	168,446	8.6
2015	22,637	0.6	23,048	0.9	92,811	3.5	93,695	4.8

Notes: [a] Aged between 13 and 24. [b] Aged between 25 and 64. [c] Aged 65 or older. [d] Water supply: Others – well, spring, and others. [e] Housing Material: No – Coated clay, uncoated clay, wood, and others. [f] Lighting: Non-electric – No meter, lamp, candle and others. [g] Average rates for the period (2007-2015) by municipality. [h] % of the municipality's population with inadequate baseline sanitation. [i] Baseline municipality unemployment rate. [j] Per 1,000 inhabitants of the baseline municipality. [l] Measured by capita expenses proportional to the baseline minimum wage (MW). [m] Quartile 1: 0% a 0.1%. [n] Quartile 2: 0.1%< a 18.5%.

Table S7. Descriptive analyses of *Programa Bolsa Família* (PBF) beneficiaries (BF) and non-beneficiaries (N-BF), 2007-2015, by subpopulation of wealth[l].

(conclusion)

Social and Demographic Variables	Quartile 3 [m] (5,699,492)				Quartile 4 [n] (5,697,196)			
	N-BF (n =3,929,830)		BF (n=1,769,662)		N-BF (n =5,081,546)		BF (n=615,650)	
	No. or Mean	(%) or CI	No. or Mean	(%) or CI	No. or Mean	(%) or CI	No. or Mean	(%) or CI
**Sex**								
Female	2,259,032	57.5	1,091,870	61.7	2,795,676	55.0	345,746	56.2
Male	1,670,798	42.5	677,792	38.3	2,285,870	45.0	269,904	43.8
**Age**								
Adolescents and Youth people[a]	764,453	19.5	519,849	29.4	905,845	17.8	196,461	31.9
Adults[b]	2,683,672	68.3	1,224,933	69.2	3,202,477	63.0	398,246	64.7
Older people[c]	481,557	12.3	24,815	1.4	973,074	19.1	20,922	3.4
**Race/ethnicity**								
White	1,393,935	38.0	603,387	36.2	2,350,542	49.5	256,093	44.2
Mixed-race	2,000,215	54.5	918,628	55.1	2,097,100	44.1	279,433	48.2
Black	267,213	7.3	136,230	8.2	296,901	6.3	43,150	7.4
Indigenous	7,327	0.2	7,497	0.5	5,403	0.1	1,165	0.2
**Education**								
More than High School	121,531	3.1	22,201	1.3	272,134	5.4	15,182	2.5
High school	1,487,124	38.4	689,277	39.4	272,134	34.7	239,190	39.1
Elementary school	1,851,348	47.8	941,191	53.8	2,490,446	49.3	322,904	52.8
Attended pre-school	45,246	1.2	13,091	0.7	59,052	1.2	4,865	0.8
Never attended school	369,177	9.5	82,297	4.7	477,238	9.4	29,506	4.8
**Water supply**								
Public network	3,079,976	81.6	1,412,975	82.5	4,397,400	88.7	539,514	90.2
Others[d]	693,025	18.4	299,508	17.5	560,156	11.3	58,375	9.8
**Housing material (Brick)**								
Yes	3,317,505	87.9	1,510,763	88.2	4,502,367	90.8	549,382	91.9
No [e]	455,485	12.1	201,717	11.8	455,189	9.2	48,507	8.1
**Lighting**								
Electricity	3,531,175	93.6	1,540,984	90.0	4,825,106	97.3	568,326	95.1
Non-electric [f]	241,811	6.4	171,500	10.0	132,450	2.7	29,563	4.9
**Region**								
North	342,943	8.7	156632	8.9	276,131	5.4	29,376	4.8
Northeast	1,135,495	28.9	349,752	19.8	863,982	17.0	67,785	11.0
Southeast	153,0555	38.9	943,848	53.3	2,359,671	46.4	374,842	60.9
South	520,589	13.2	178,272	10.1	975,302	19.2	86,001	14.0
Central-west	400,242	10.2	141,158	8.0	606,457	11.9	57,646	9.4
**Area of residence**								
Rural	615,680	15.7	217,673	12.3	473,355	9.3	39,291	6.4
Urban	3,313,139	84.3	1,550,783	87.7	4,607,883	90.7	576,143	93.6
**Average AIDS incidence rate[g]**	19.85	(19.8-19.9)	23.06	(23.0-23.1))	21.36	(21.3-21.4)	24.43	(24.3-24.4)
**Average AIDS mortality rate[g]**	5.84	(5.84-5.85)	6.92	(6.92-6.93)	6.31	(6.30-6.31)	7.33	(7.32-7.34)
**Average AIDS case-fatality rate[g]**	30.82	(30.8-30.8)	30.98	(30.9-30.9)	31.08	(31.0-31.1)	31.05	(31.0-31.1)
**Inadequate sanitation [h]**	8.10	(8.09-8.11)	7.64	(7.63-7.66)	5.50	(5.49-5.50)	5.55	(5.53-5.57)

**Unemployment rate (%)[i]**	6.56	(6.55-6.56)	6.93	(6.92-6.93)	5.66	(5.66-5.67)	6.12	(6.12-6.13)
**Doctors per 1000 inhabitants[j]**	1.43	(1.43-1.43)	1.74	(1.74-1.74)	1.63	(1.63-1.64)	1.96	(1.96-1.97)
**Nurses per 1000 inhabitants[j]**	0.78	(0.78-0.78)	0.82	(0.82-0.82)	0.84	(0.84-0.84)	0.89	(0.89-0.89)
**Hospital beds per 1000 inhabitants[j]**	2.27	(2.27-2.27)	2.32	(2.32-2.32)	2.42	(2.41-2.42)	2.43	(2.43-2.43)
**Year of entry into the cohort**								
2007	88,787	2.3	35,659	2.0	18,795	0.4	6,375	1.0
2008	70,951	1.8	26,099	1.5	20,005	0.4	4,876	0.8
2009	61,580	1.6	47,607	2.7	16,591	0.3	7,304	1.2
2010	181,802	4.6	179,938	10.2	153,133	3.0	43,829	7.1
2011	398,980	10.2	364,879	20.6	662,567	13.0	138,944	22.6
2012	974,815	24.8	385,680	21.8	1,287,919	25.3	139,691	22.7
2013	774,582	19.7	332,547	18.8	980,642	19.3	114,700	18.6
2014	848,225	21.6	237,919	13.4	1,136,268	22.4	91,965	14.9
2015	530,108	13.5	159,334	9.0	805,626	15.9	67,966	11.0

Notes: [a] Aged between 13 and 24. [b] Aged between 25 and 64. [c] Aged 65 or older. [d] Water supply: Others – well, spring, and others. [e] Housing Material: No – Coated clay, uncoated clay, wood, and others. [f] Lighting: Non-electric – No meter, lamp, candle and others. [g] Average rates for the period (2007-2015) by municipality. [h] % of the municipality's population with inadequate baseline sanitation. [i] Baseline municipality unemployment rate. [j] Per 1,000 inhabitants of the baseline municipality. [l] Measured by capita expenses proportional to the baseline minimum wage (MW). [m] Quartile 1: 0% a 0.1%. [n] Quartile 2: 0.1%< a 18.5%.

Table S8. Descriptive analyses of *Programa Bolsa Família* (PBF) beneficiaries (BF) and non-beneficiaries (N-BF) (n = 22,788,998), 2007-2015, by subpopulation of sex.

Social and Demographic Variables	Female (n=13,214,290)				Male (n=9,574,707)			
	N-BF (n = 8,707,472)		BF (n= 4,506,818)		N-BF (n = 6,870,704)		BF (n= 2,704,003)	
	No. Or Mean	(%) or CI	No. or Mean	(%) or CI	No. or Mean	(%) or CI	No. or Mean	(%) or CI
**Age**								
Adolescents and Youth people[a]	2,176,650	25.0	1,513,254	33.6	1,550,280	22.6	852,547	31.5
Adults[b]	5,457,533	62.7	2,939,372	65.2	4,418,865	64.3	1,807,010	66.8
Older people[c]	1,072,975	12.3	54,051	1.0	901,321	13.1	44,304	1.6
**Race/ethnicity**								
White	3,245,293	39.2	1,360,328	31.4	2,401,993	37.1	779,451	29.9
Mixed-race	4,440,033	53.6	2,516,906	58.2	3,580,946	55.4	1,566,981	60.2
Black	587,437	7.1	397,646	9.2	471,281	7.3	219,749	8.4
Indigenous	16,406	0.2	52,075	1.2	14,235	0.2	37,656	1.4
**Education**								
More than High School	319,297	4.0	46,679	1.1	148,942	2.3	18,805	0.7
High school	2,632,478	32.7	1,303,879	31.9	1,666,905	25.9	550,640	21.8
Elementary school	4,196,733	52.1	2,445,932	59.9	3,768,595	58.6	1,695,479	67.1
Attended pre-school	85,004	1.1	28,694	0.7	78,852	1.2	22,440	0.9
Never attended school	829,114	10.3	261,111	6.4	768,592	11.9	238,259	9.4
**Wealth[d]**								
Level 1 (More wealth)	963,008	11.1	69,907	1.6	815,135	11.9	58,236	2.2
Level 2	2,218,077	25.5	372,416	8.3	1,767,097	25.7	280,638	10.4
Level 3	1,508,594	17.3	686,568	15.2	1,115,490	16.2	440,889	16.3
Level 4	2,463,074	28.3	2,249,490	49.9	1,641,204	23.9	1,127,953	41.7
Level 5 (Lower wealth)	1,552,174	17.8	1,127,656	25.0	1,529,777	22.3	795,743	29.4
**Water supply**								
Public network	6,861,528	81.1	3,374,504	76.7	5,168,528	77.7	1,873,495	71.7
Others[e]	1,595,827	18.9	1,023,807	23.3	1,486,033	22.3	739,641	28.3
**Housing material (Brick)**								
Yes	7,200,335	85.1	3,656,219	83.1	5,515,666	82.9	2,076,683	79.5
No[f]	1,256,961	14.9	742,044	16.9	1,138,768	17.1	536,393	20.5
**Lighting**								
Electricity	7,797,199	92.2	3,733,456	84.9	6,099,352	91.7	2,220,038	85.0
Non-electric[g]	660,151	7.8	664,841	15.1	555,139	8.3	393,076	15.0
**Region**								
North	831,869	9.6	549,045	12.2	637,732	9.3	333,855	12.3
Northeast	2,596,720	29.8	1,308,628	29.0	2,052,882	29.9	837,692	31.0
Southeast	3,286,580	37.7	1,991,894	44.2	2,514,999	36.6	1,102,242	40.8
South	1,163,102	13.4	351,413	7.8	1,005,026	14.6	240,617	8.9
Central-west	829,091	9.5	305,824	6.8	659,786	9.6	189,575	7.0
**Area of residence**								
Rural	1,336,718	15.4	684,767	15.2	1,365,766	19.9	599,734	22.4
Urban	7,356,304	84.6	3,818,402	84.8	5,480,414	80.1	2,080,772	77.6
**Average AIDS incidence rate[h]**	19.78	(19.7-19.7)	22.71	(22.7-22.7)	18.33	(18.3-19.3)	19.91	(19.9-19.9)
**Average AIDS mortality rate[h]**	5.82	(5.8-5.8)	6.77	(6.7-6.8)	5.38	(5.3-5.4)	5.91	(5.9-5.9)

**Average AIDS case-fatality rate[h]**	30.84	(30.8-30.8)	30.84	(30.8-30.8)	30.95	(30.9-30.9)	30.90	(30.9-30.9)
**Inadequate sanitation [i]**	9.80	(9.8-9.8)	10.34	(10.3-10.3)	10.27	(10.2-10.3)	11.81	(11.7-11.8)
**Unemployment rate (%)[j]**	7.45	(7.4-7.4)	8.55	(8.5-8.6)	7.29	(7.2-7.3)	8.30	(8.2-8.3)
**Doctors per 1000 inhabitants[l]**	1.38	(1.3-1.4)	1.52	(1.5-1.5)	1.29	(1.2-1.3)	1.36	(1.3-1.4)
**Nurses per 1000 inhabitants[l]**	0.70	(0.7-0.7)	0.68	(0.6-0.7)	0.68	(0.6-0.7)	0.65	(0.6-0.6)
**Hospital beds per 1000 inhabitants[l]**	2.34	(2.3-2.3)	2.36	(2.3-2.4)	2.29	(2.2-2.3)	2.27	(2.2-2.3)
**Year of entry into the cohort**								
2007	1,234,100	14.2	712,526	15.8	1,152,390	16.8	515,084	19.0
2008	839,255	9.6	412,118	9.1	627,664	9.1	258,415	9.6
2009	486,319	5.6	720,710	16.0	402,380	5.9	408,408	15.1
2010	764,113	8.8	628,983	14.0	530,585	7.7	305,658	11.3
2011	690,359	7.9	520,780	11.6	547,652	8.0	314,080	11.6
2012	1,513,187	17.4	525,116	11.7	1,112,118	16.2	300,914	11.1
2013	1,114,190	12.8	452,833	10.0	873,458	12.7	265,938	9.8
2014	1,266,338	14.5	327,330	7.3	972,886	14.2	197,885	7.3
2015	799,611	9.2	206,422	4.6	651,571	9.5	137,621	5.1

Notes: [a] Aged between 13 and 24. [b] Aged between 25 and 64. [c] Aged 65 or older. [d] Measured by capita expenses proportional to the baseline minimum wage (MW). Level 1 (More wealth): "1 or more". Level 2: "0.5 to 1". Level 3: "0.25 to 0.49". Level 4: "0< to 0.24". Level 5 (Lower wealth): "Nothing declared". [e] Water supply: Others – well, spring, and others. [f] Housing Material: No – Coated clay, uncoated mud, wood and others. [g] Lighting: Non-electric – No meter, lamp, candle and others. [h] Average rates for the period (2007-2015) by municipality. [i] % of the municipality's population with inadequate baseline sanitation. [j] Baseline municipality unemployment rate. [l] Per 1,000 inhabitants of the baseline municipality.

Table S9. Descriptive analyses of *Programa Bolsa Família* (PBF) beneficiaries (BF) and non-beneficiaries (N-BF) (n = 22,788,998), 2007-2015, by subpopulation of age.

Social and Demographic Variables	Adolescents and Youth people (n=6,092,731) 13=< a <=24				Adults (n=14,622,780) 25<= a <=64				Older people (n=2,072,651) 65<= or more			
	N-BF (n =3,726,930)		BF (n= 2,365,801)		N-BF (n = 9,876,398)		BF (n= 4,746,382)		N-BF (n =1,974,296)		BF (n= 98,355)	
	No. or Mean	(%) or CI	No. or Mean	(%) or CI	No. or Mean	(%) or CI	No. or Mean	(%) or CI	No. or Mean	(%) or CI	No. or Mean	(%) or CI
**Sex**												
Females	2,176,650	58.4	1,513,254	64.0	5,457,533	55.3	2,939,372	61.9	1,072,975	54.3	54,051	55.0
Male	1,550,280	41.6	852,547	36.0	4,418,865	44.7	1,807,010	38.1	901,321	45.7	44,304	45.0
**Race/ethnicity**												
White	1,234,669	34.6	659,316	28.6	3,546,038	38.2	1,444,530	31.9	866,392	45.5	35,854	37.7
Mixed-race	2,101,870	59.0	1,428,790	62.0	5,018,659	54.0	2,606,284	57.5	900,128	47.3	48,646	51.1
Black	217,243	6.1	175,121	7.6	708,529	7.6	432,782	9.6	132,907	7.0	9,460	9.9
Indigenous	9,834	0.3	41,999	1.8	16,542	0.2	46,503	1.0	4,261	0.2	1,224	1.3
**Education**												
More than High School	97,039	2.9	13,016	0.6	353,539	3.9	51,88	1.2	17,651	0.9	580	0.6
High school	1,396,671	41.4	706,670	32.1	2,798,764	30.5	1,143,385	26.5	103,803	5.3	4,401	4.6
Elementary school	1,759,634	52.1	1,407,656	64.0	5,074,560	55.3	2,682,497	62.2	1,130,879	58.2	51,139	53.4
Attended pre-school	20,253	0.6	10,473	0.5	99,056	1.1	38,840	0.9	44,534	2.3	1,817	1.9
Never attended school	101,697	3.0	61,876	2.8	850,459	9.3	399,504	9.3	645,451	33.2	37,913	39.6
**Wealth**[a]												
Level 1 (More wealth)	336,249	9.0	41,448	1.8	1,095,061	11.1	80,703	1.7	346,787	17.6	5,989	6.1
Level 2	685,791	18.4	205,547	8.7	2,557,625	25.9	428,975	9.0	741,631	37.6	18,507	18.8
Level 3	498,644	13.4	329,394	13.9	1,800,362	18.2	781,480	16.5	324,982	16.5	16,543	16.8
Level 4	1,068,041	28.7	1,053,941	44.6	2,664,887	27.0	2,289,123	48.2	371,185	18.8	34,237	34.8
Level 5 (Lower wealth)	1,137,213	30.5	735,038	31.1	1,755,768	17.8	1,165,236	24.6	188,853	9.6	23,052	23.4
**Water supply**												
Public network	2,731,855	76.1	1,666,743	72.4	7,709,671	80.3	3,502,771	75.9	1,588,148	82.6	78,297	82.0
Others[b]	858,136	23.9	635,466	27.6	1,888,759	19.7	1,110,673	24.1	334,816	17.4	17,218	18.0
**Housing material (Brick)**												
Yes	2,891,258	80.5	1,811,451	78.7	8,168,997	85.1	3,839,540	83.2	1,655,330	86.1	81,692	85.5
No[c]	698,602	19.5	490,686	21.3	1,429,375	14.9	773,871	16.8	267,637	13.9	13,820	14.5
**Lighting**												
Electricity	3,197,015	89.1	1,906,435	82.8	8,852,720	92.2	3,959,733	85.8	1,846,343	96.0	87,100	91.2
Non-electric[d]	392,943	10.9	395,751	17.2	745,659	7.8	653,701	14.2	76,630	4.0	8,412	8.8
**Region**												
North	438,332	11.8	342,721	14.5	876,431	8.9	531,623	11.2	154,756	7.8	8,512	8.7
Northeast	1,234,940	33.1	758,929	32.1	2,849,243	28.8	1,361,791	28.7	565,207	28.6	25,498	25.9
Southeast	1,245,812	33.4	924,304	39.1	3,784,988	38.3	2,122,678	44.7	770,627	39.0	47,057	47.8
South	484,904	13.0	182,851	7.7	1,388,463	14.1	399,576	8.4	294,708	14.9	9,580	9.7
Central-west	322,724	8.7	156,971	6.6	977,103	9.9	330,703	7.0	188,997	9.6	7,708	7.8
**Area of residence**												
Rural	745,804	20.2	476,805	20.2	1,625,723	16.5	794,281	16.8	330,834	16.8	13,343	13.6
Urban	2,950,247	79.8	1,885,207	79.8	8,243,122	83.5	3,928,986	83.2	1,642,921	83.2	84,770	86.4

**Average AIDS incidence rate**[e]	18.58	(18.5-18.6)	20.88	(20.8-20.9)	19.64	(19.6-19.6)	22.05	(22.0-22.0)	17.67	(17.6-17.7)	22.01	(21.9-22.1)
**Average AIDS mortality rate**[e]	5.43	(5.4-5.4)	6.20	(6.1-6.2)	5.77	(5.7-5.8)	6.57	(6.5-6.6)	5.29	(5.2-5.3)	6.59	(6.5-6.6)
**Average AIDS case-fatality rate**[e]	30.73	(30.7-30.7)	30.85	(30.8-30.8)	30.81	(30.8-30.8)	30.86	(30.8-30.9)	31.64	(31.6-31.6)	31.09	(31.0-31.1)
**Inadequate sanitation**[f]	12.04	(12.0-12.1)	12.38	(12.3-12.4)	9.38	(9.3-9.4)	10.18	(10.1-10.2)	9.33	(9.3-9.3)	9.35	(9.2-9.4)
**Unemployment rate (%)**[g]	7.82	(7.8-7.8)	8.56	(8.5-8.6)	7.34	(7.3-7.3)	8.41	(8.4-8.4)	6.77	(6.7-6.8)	8.19	(8.1-8.2)
**Doctors per 1000 inhabitants**[h]	1.25	(1.2-1.2)	1.37	(1.3-1.4)	1.39	(1.3-1.4)	1.51	(1.5-1.5)	1.28	(1.2-1.3)	1.54	(1.5-1.5)
**Nurses per 1000 inhabitants**[h]	0.64	(0.6-0.6)	0.64	(0.6-0.6)	0.70	(0.7-0.7)	0.68	(0.6-0.7)	0.70	(0.7-0.7)	0.69	(0.6-0.7)
**Hospital beds per 1000 inhabitants**[h]	2.26	(2.2-2.2)	2.27	(2.2-2.2)	2.34	(2.3-2.3)	2.35	(2.3-2.4)	2.32	(2.3-2.3)	2.42	(2.4-2.4)
**Year of entry into the cohort**												
2007	905,898	24.3	462,379	19.5	1,365,505	13.8	748,622	15.8	115,011	5.8	16,570	16.8
2008	407,993	10.9	221,929	9.4	890,541	9.0	437,614	9.2	168,344	8.5	10,966	11.1
2009	215,163	5.8	367,504	15.5	554,350	5.6	748,325	15.8	119,164	6.0	13,251	13.5
2010	313,096	8.4	301,794	12.8	844,409	8.5	623,889	13.1	137,144	6.9	8,934	9.1
2011	209,991	5.6	257,924	10.9	785,768	8.0	562,503	11.9	242,212	12.3	14,404	14.6
2012	498,490	13.4	249,160	10.5	1,707,678	17.3	564,355	11.9	418,983	21.2	12,457	12.7
2013	413,770	11.1	225,880	9.5	1,333,515	13.5	483,097	10.2	240,296	12.2	9,578	9.9
2014	464,163	12.5	166,958	7.1	1,463,360	14.8	350,856	7.4	311,638	15.8	7,373	7.5
2015	298,366	8.0	112,273	4.7	931,272	9.4	227,121	4.8	221,504	11.2	4,642	4.7

Notes: [a] Measured by capita expenses proportional to the baseline minimum wage (MW). Level 1 (More wealth): “1 or more”. Level 2: “0.5 to 1”. Level 3: “0.25 to 0.49”. Level 4: “0< to 0.24”. Level 5 (Lower wealth): “Nothing declared”. [b] Water supply: Others – well, spring, and others. [c] Housing Material: No – Coated clay, uncoated clay, wood, and others. [d] Lighting: Non-electric – No meter, lamp, candle and others. [e] Average rates for the period (2007-2015) by municipality. [f] % of the municipality's population with inadequate baseline sanitation. [g] Baseline municipality unemployment rate. [h] Per 1,000 inhabitants of the baseline municipality.

3. Estimation of logistic regression by outcome

For each dataset by outcome variable, we estimated the probability of the individual being exposed to the PBF through multivariate logistic regression adjusted for the relevant demographic and socioeconomic covariates (Table S10). Covariates include sex, race/ethnicity, age, education, per capita wealht, AIDS treatment, water supply, housing material, lighting, region and area of residence, year of entry into the cohort, and municipal-level covariates such as average AIDS incidence, mortality and fatality rates, inadequate sanitation, unemployment rate, and rate of doctors, nurses and beds per 1000 inhabitants.

Table S10. Logistic regression prediction models for individuals benefiting or not from the *Programa Bolsa Família* (PBF), for AIDS incidence, mortality and case-fatality rate, 2007-2015. Estimates for the Propensity Score (PS).

Variables	Outcomes (OR[a] – IC[b] 95%)		
	Incidence	Mortality	Case-Fatality
**Sex**			
Female	1 (base)	1 (base)	1 (base)
Male	0.84*** (0.84-0.85)	0.85***(0.84-0.85)	0.5264***(0.48-0.57)
**Age**			
Adolescents and Youth people[c]	1 (base)	1 (base)	1 (base)
Adults[d]	0.77***(0.76-0.77)	0.77***(0.77-0.77)	0.66***(0.58-0.74)
Older people[e]	0.10***(0.10-0.10)	0.10***(0.10-0.10)	0.08***(0.04-0.14)
**Race/ethnicity**			
White	1 (base)	1 (base)	1 (base)
Mixed-race	1.07***(1.07-1.08)	1.05***(1.04-1.05)	1.01 (0.90-1.13)
Black	1.12***(1.11-1.12)	1.11***(1.10-1.11)	1.21* (1.05-1.41)
Indigenous	6.27***(6.17-6.36)	6.39***(6.29-6.49)	2.37* (1.11-5.08)
**Education**			
More than High School	1 (base)	1 (base)	1 (base)
High school	2.27***(2.24-2.29)	2.18***(2.16-2.20)	2.60***(1.80-3.76)
Elementary school	2.62***(2.60-2.65)	2.53***(2.51-2.56)	3.17***(2.21-4.54)
Attended pre-school	2.20***(2.17-2.23)	2.16***(2.13-2.19)	2.29* (1.18-4.46)
Never attended school	2.30***(2.28-2.33)	2.22***(2.19-2.24)	2.48***(1.68-3.67)
**Wealth[f]**			
Level 1 (More wealth)	1 (base)	1 (base)	1 (base)
Level 2	2.28***(2.26-2.29)	2.23***(2.22-2.25)	1.81***(1.35-2.43)
Level 3	5.66***(5.62-5.69)	5.55***(5.52-5.59)	4.99***(3.76-6.63)
Level 4	10.59***(10.5-10.6)	10.6***(10.5-10.6)	8.66***(6.55-11.44)
Level 5 (Lower wealth)	9.14***(9.07-9.21)	9.21***(9.14-9.27)	10.10***(7.55-13.51)
**AIDS Treatment**			
Yes	x	x	1 (base)
No	x	x	1.12* (1.01-1.25)
**Water supply**			
Public network	1 (base)	1 (base)	1 (base)
Others[g]	1.14***(1.14-1.15)	1.17***(1.16-1.17)	1.18* (1.03-1.35)
**Housing material (Brick)**			
Yes	1 (base)	1 (base)	1 (base)
No[h]	1.08***(1.08-1.09)	1.06***(1.05-1.06)	0.97 (0.85-1.10)
**Lighting**			
Electricity	1 (base)	1 (base)	1 (base)
Non-electric[i]	1.27***(1.24-1.25)	1.21***(1.21-1.22)	1.57***(1.38-1.79)
**Region**			
North	1 (base)	1 (base)	1 (base)
Northeast	0.91***(0.91-0.92)	0.86***(0.85-0.86)	1.02 (0.85-1.23)
Southeast	1.34***(1.33-1.35)	1.09***(1.08-1.09)	1.30* (1.06-1.60)
South	0.67***(0.67-0.68)	0.74***(0.74-0.75)	0.84 (0.67-1.05)
Central-west	0.85***(0.85-0.86)	0.82***(0.81-0.82)	0.77* (0.60-1.00)
**Area of residence**			
Rural	1 (base)	1 (base)	1 (base)
Urban	1.04***(1.04-1.05)	1.04***(1.03-1.04)	0.80* (0.64-1.00)
**Average AIDS incidence rate[j]**	1.01***(1.01-1.01)	x	x
**Average AIDS mortality rate[j]**	x	1.02***(1.02-1.03)	x
**Average AIDS case-fatality rate[j]**	x	x	1.00* (1.00-1.01)
**Inadequate sanitation[l]**	0.99***(0.99-0.99)	0.99***(0.99-0.99)	0.99 (0.98-1.00)
**Unemployment rate (%)[m]**	1.01***(1.01-1.01)	1.02***(1.02-1.02)	1.02* (1.00-1.04)
**Doctors per 1000 inhabitants[n]**	1.12***(1.11-1.12)	1.06***(1.06-1.07)	0.93 (0.86-1.01)
**Nurses per 1000 inhabitants[n]**	1.03***(1.02-1.03)	0.69***(0.69-0.70)	1.47** (1.16-1.87)
**Hospital beds per 1000 inhabitants[n]**	0.97***(0.97-0.97)	1.00***(0.69-0.70)	0.99 (0.95-1.03)
**Year of entry into the cohort**	yes	yes	yes
Obs.:	19,577,880	19,577,880	10,423

Notes: *** p-value <0,001; **p-valor <0,01; *p-valor <0,05. [a] Odds Ratio. [b] Confidence Interval. [c] Aged between 13 and 24. [d] Aged between 25 and 64. [e] Aged 65 or older. [f] Measured by capita expenses proportional to the baseline minimum wage (MW). Level 1 (More wealth): "1 or more". Level 2: "0.5 to 1". Level 3: "0.25 to 0.49". Level 4: "0< to 0.24". Level 5 (Lower wealth): "Nothing declared". [g] Water supply: Other – well, spring, and others. [h] Housing Material: No – Coated clay, uncoated clay, wood, and others. [i] Lighting: Non-electric – No meter, lamps, candles, and others. [j] Average rates for the period (2007-2015) by municipality. [l] % of the municipal population with inadequate baseline sanitation. [m] Baseline municipal unemployment rate. [n] Per 1,000 inhabitants of the baseline municipality. All statistical tests used where two-sided and, where appropriate, adjustments were made for multiple comparisons.

4. Sensitivity Analyses

Several sensitivity analyses were performed. First, to observe the importance of adopting variables that measure characteristics at the municipal level, three models were tested with different groups of variables at the aggregate level, holding the endemicity variables (i.e., incidence, mortality, and fatality rates) constant. The groups of variables in each model are described as follow: i) percentage of the municipal population with garbage collection, GDP per capita, proportion of poor people and rate of specialized clinics per 1,000 inhabitants (Table S11); ii) GDP per capita and proportion of the poor population were replaced by per capita income and proportion of extremely poor population (Table S12); iii) the income variable was replaced by the municipal Gini index (Table S13). The entire set of estimates showed similar effects of the PBF on the outcomes in terms of direction, magnitude, and statistical significance, compared to those presented in the main result.

Second, to investigate the influence of the municipal AIDS endemicity levels on the estimates, the same regressions described above were performed in different conditions: (i) estimates without the municipal average rate of each outcome (incidence, mortality and case-fatality) holding the remaining variables aggregated in the models (Table S14); (ii) in addition to the variables at the individual level, only the remained municipal endemic variables (Table S15), and; (iii) without considering any aggregated variables (the results of the unadjusted and adjusted models for relevant covariates are presented in Table S16).

Third, to verify the effect of the adoption of the per capita wealth variable, the estimates were performed for the three outcomes using the per capita income variable rather than the per capita wealth (Table S17). The per capita wealth variable was adopted as a proxy for per capita income because the income variable is self-declared by individuals when registering in the CadÚnico, and, considering that having the per capita income below certain thresholds is one of the main prerequisites for receiving any social program from the government, it could be underreported. The wealth, mensured by the per capita expenditure is not considered and is not perceived by the interviewed, as an elegibility criteria and for that reason could suffer less of underreporting bias.

Fourth, to verify the relevance of the IPTW to the unbiased estimates of the PBF, we performed the same multivariate Poisson regressions without the IPTW and compared the results with the IPTW (Table S18). All the PBF effects found on the outcomes were comparable with the main results. However, the PBF impacts were considerably stronger on AIDS incidence and mortality rates without weighting IPTW regression.

Fifth, we estimate a specific model without robust cluster standard errors at the individual level (Table S19).

Sixth, in Table S20, we estimate the models only with the selection of municipalities with adequate vital information quality. Although the 17% reduction in the number of observations (about 3,390,00 individuals) in the incidence and mortality models and 4% (395 individuals) in case-fatality, the results did not presented significant changes.

Seventh, additionally, in Table S21, we estimated the association of PBF for all individuals, that is, participants with complete data on all covariates and participants with missing data for one or more variables in estimating PS and adjusted Poisson model.

Eighth, methodological triangulation is a practice adopted to obtain more reliable answers to research problems.[10] Through the integration of different methodologies, if the results of the different approaches point to the same conclusion, this strengthens the confidence of the findings, mainly in the scope of causal inference. In our study, in addition to the Poisson model (main model), as a complementary analysis, we included survival models (cox proportional hazard model) and propensity score matching to verify the robustness of the results. With the same set of covariates for adjusted, the three models showed, on average, the same magnitude of the effect of the PBF on the incidence, mortality and case fatality due to AIDS in the most socioeconomically vulnerable population in Brazil, between 2007 and 2015 (Table S22). For the survival model, being exposed to PBF reduces the relative risk (hazard ratio) of incidence {35% [= (0.65-1) *100%]}, mortality {35% [= (0.65-1) *100%]} and case fatality {14% [= (0.86-1) *100%]} by AIDS. In PSM, in the overall matched dataset, individuals who received cash transfers had lower odds of incidence [32% (OR 0.68, 95% CI 0.65-0.70)], mortality [32% (OR 0.68, 95% CI 0.64-0.71)] and case fatality [54% (OR 0.46, 95% CI 0.35-0.55)] by AIDS than those who did not.

Table S11. Estimates of the average effect of the *Programa Bolsa Família* (PBF), in unadjusted and adjusted Poisson models (with robust standard error), on AIDS incidence, mortality and case-fatality rate, 2007-2015 - Group 1 of aggregated variables at the municipal level.

Models	Outcomes (IRR[a] – IC[b] 95%)		
	**Incidence**	**Mortality**	**Case-Fatality**
**Unadjusted Models**			
**PBF**	0.66*** (0.64-0.68)	0.67*** (0.63-0.70)	0.73*** (0.65-0.82)
**Adjusted Model**			
**PBF**	0.59*** (0.57-0.61)	0.60*** (0.56-0.63)	0.74*** (0.66-0.84)
**Sex**			
Female	1 (base)	1 (base)	1 (base)
Male	1.21*** (1.17-1.25)	1.34*** (1.27-1.42)	1.29*** (1.15-1.46)
**Age**			
Adolescents and Youth people[c]	1 (base)	1 (base)	1 (base)
Adults[d]	2.02*** (1.93-2.10)	3.50*** (3.20-3.82)	1.66*** (1.38-2.01)
Older people[e]	0.32*** (0.28-0.37)	0.78* (0.64-0.95)	2.75*** (1.72-4.38)
**Race/ethnicity**			
White	1 (base)	1 (base)	1 (base)
Mixed-race	1.25*** (1.20-1.30)	1.25*** (1.17-1.34)	1.08 (0.93-1.26)
Black	1.67*** (1.58-1.76)	1.81*** (1.67-1.98)	1.02 (0.84-1.24)
Indigenous	1.37* (1.01-1.85)	0.92 (0.51-1.65)	0.59 (0.21-1.66)
**Education**			
More than High School	1 (base)	1 (base)	1 (base)
High school	1.04 (0.91-1.19)	1.49** (1.12-2.00)	1.94* (1.05-3.59)
Elementary school	1.41*** (1.24-1.60)	2.71*** (2.04-3.60)	2.77** (1.51-5.08)
Attended pre-school	0.90 (0.71-1.14)	1.30 (0.81-2.08)	1.04 (0.37-2.91)
Never attended school	1.29*** (1.12-1.48)	2.86*** (2.13-3.84)	3.69*** (1.96-6.93)
**Wealth[f]**			
Level 1 (More wealth)	1 (base)	1 (base)	1 (base)
Level 2	0.99 (0.89-1.09)	0.92 (0.77-1.09)	1.25 (0.81-1.91)
Level 3	1.16** (1.06-1.28)	1.21* (1.02-1.43)	1.63* (1.07-2.46)
Level 4	1.58*** (1.43-1.74)	1.56*** (1.32-1.85)	1.34 (0.88-2.02)
Level 5 (Lower wealth)	2.14*** (1.94-2.37)	2.35*** (1.98-2.80)	1.59* (1.04-2.45)
**AIDS Treatment**			
Yes	x	x	1 (base)
No	x	x	2.63*** (2.29-3.03)
**Water supply**			
Public network	1 (base)	1 (base)	1 (base)
Others[g]	1.02 (0.98-1.08)	1.09* (1.00-1.18)	1.01 (0.85-1.19)
**Housing material (Brick)**			
Yes	1 (base)	1 (base)	1 (base)
No[h]	1.24*** (1.18-1.30)	1.35*** (1.25-1.46)	1.19* (1.01-1.40)
**Lighting**			
Electricity	1 (base)	1 (base)	1 (base)
Non-electric[i]	1.30*** (1.24-1.37)	1.36*** (1.25-1.47)	1.23* (1.04-1.45)
**Region**			
North	1 (base)	1 (base)	1 (base)
Northeast	1.20*** (1.13-1.29)	1.09 (0.97-1.22)	1.04 (0.80-1.34)
Southeast	1.15*** (1.07-1.23)	1.29*** (1.14-1.46)	0.96 (0.71-1.29)
South	1.42*** (1.31-1.54)	1.27** (1.10-1.46)	0.85 (0.62-1.16)
Central-west	1.19*** (1.09-1.30)	1.13 (0.97-1.32)	0.97 (0.69-1.36)
**Area of residence**			
Rural	1 (base)	1 (base)	1 (base)
Urban	1.90*** (1.77-2.04)	2.20*** (1.95-2.48)	1.04 (0.78-1.39)
**Average AIDS incidence rate[j]**	1.02*** (1.02-1.03)	x	x
**Average AIDS mortality rate[j]**	x	1.09*** (1.09-1.10)	x
**Average AIDS case-fatality rate[j]**	x	x	1.01*** (1.00-1.02)
**Garbage collection[l]**	1.00*** (1.00-1.00)	1.00* (1.00-1.00)	0.99 (0.97-1.00)
**Per capita GDP[m]**	0.99*** (0.99-0.99)	0.99* (0.99-1.00)	0.99* (0.99-0.99)
**Proportion of Poor[n]**	0.99 (0.99-1.00)	0.99* (0.98-0.99)	0.98* (0.96-0.99)
**Specialized Clinic rate[o]**	0.99 (0.85-1.14)	0.93 (0.72-1.19)	0.79 (0.42-1.48)
**Year of entry into the cohort**	yes	yes	yes
Obs.:	19,577,629	19,577,649	9,965

Notes: *** p-value <0,001; **p-valor <0,01; *p-valor <0,05. [a] Incidence Rate Ratios. [b] Confidence Interval. [c] Aged between 13 and 24. [d] Aged between 25 and 64. [e] Aged 65 or older. [f] Measured by capita expenses proportional to the baseline minimum wage (MW). Level 1 (More wealth): "1 or more". Level 2: "0.5 to 1". Level 3: "0.25 to 0.49". Level 4: "0< to 0.24". Level 5 (Lower wealth): "Nothing declared". [g] Water supply: Others – well, spring, and others. [h] Housing Material: No – Coated clay, uncoated clay, wood, and others. [i] Lighting: Non-electric – No meter, lamp, candle and others. [j] Average rates for the period (2007-2015) by municipality. [l] % of municipality population with baseline garbage collection. [m] Gross Domestic Product (GDP) per capita at baseline. [n] % poor in the baseline municipality (according to the nominal minimum wage: earn between 1/4 and 1/2 salary). [o] Per 1,000 inhabitants of the baseline municipality. All statistical tests used where two-sided and, where appropriate, adjustments were made for multiple comparisons.

Table S12. Estimates of the average effect of the *Programa Bolsa Família* (PBF), in unadjusted and adjusted Poisson models (with robust standard error), on AIDS incidence, mortality and case-fatality rate, 2007-2015 - Group 2 of aggregated variables at the municipal level.

Models	Outcomes (IRR[a] – IC[b] 95%)		
	**Incidence**	**Mortality**	**Case-Fatality**
**Unadjusted Models**			
**PBF**	0.67*** (0.65-0.69)	0.67*** (0.64-0.71)	0.73*** (0.65-0.82)
**Adjusted Model**			
**PBF**	0.59*** (0.57-0.61)	0.60*** (0.57-0.64)	0.75*** (0.66-0.85)
**Sex**			
Female	1 (base)	1 (base)	1 (base)
Male	1.21*** (1.17-1.25)	1.34*** (1.27-1.42)	1.30*** (1.15-1.46)
**Age**			
Adolescents and Youth people[c]	1 (base)	1 (base)	1 (base)
Adults[d]	2.02*** (1.94-2.11)	3.53*** (3.23-3.85)	1.66*** (1.38-2.01)
Older people[e]	0.32*** (0.28-0.37)	0.78* (0.63-0.95)	2.73*** (1.72-4.32)
**Race/ethnicity**			
White	1 (base)	1 (base)	1 (base)
Mixed-race	1.25*** (1.20-1.30)	1.25*** (1.17-1.34)	1.07 (0.92-1.25)
Black	1.67*** (1.58-1.76)	1.81*** (1.66-1.97)	1.00 (0.83-1.22)
Indigenous	1.39* (1.02-1.88)	0.93 (0.52-1.66)	0.59 (0.21-1.67)
**Education**			
More than High School	1 (base)	1 (base)	1 (base)
High school	1.03 (0.90-1.18)	1.47** (1.10-1.97)	1.90* (1.00-3.61)
Elementary school	1.40*** (1.23-1.60)	2.64*** (1.98-3.52)	2.65** (1.41-5.00)
Attended pre-school	0.89 (0.70-1.13)	1.27 (0.79-2.04)	0.98 (0.34-2.80)
Never attended school	1.27** (1.10-1.46)	2.78*** (2.07-3.74)	3.51*** (1.82-6.77)
**Wealth[f]**			
Level 1 (More wealth)	1 (base)	1 (base)	1 (base)
Level 2	0.98 (0.89-1.09)	0.91 (0.76-1.07)	1.25 (0.82-1.91)
Level 3	1.16** (1.06-1.28)	1.21* (1.02-1.43)	1.64* (1.09-2.48)
Level 4	1.57*** (1.43-1.73)	1.54*** (1.30-1.83)	1.35 (0.90-2.03)
Level 5 (Lower wealth)	2.14*** (1.93-2.37)	2.35*** (1.97-2.80)	1.62* (1.06-2.47)
**AIDS Treatment**			
Yes	x	x	1 (base)
No	x	x	2.64*** (2.29-3.05)
**Water supply**			
Public network	1 (base)	1 (base)	1 (base)
Others[g]	1.01 (0.96-1.06)	1.08* (1.00-1.17)	1.01 (0.85-1.20)
**Housing material (Brick)**			
Yes	1 (base)	1 (base)	1 (base)
No [h]	1.24*** (1.18-1.30)	1.34*** (1.25-1.45)	1.19* (1.01-1.40)
**Lighting**			
Electricity	1 (base)	1 (base)	1 (base)
Non-electric [i]	1.33*** (1.26-1.39)	1.38*** (1.27-1.50)	1.22* (1.03-1.44)
**Region**			
North	1 (base)	1 (base)	1 (base)
Northeast	1.23*** (1.15-1.31)	1.08 (0.96-1.21)	1.04 (0.81-1.34)
Southeast	1.20*** (1.12-1.28)	1.33*** (1.18-1.50)	1.01 (0.76-1.35)
South	1.44*** (1.33-1.55)	1.31*** (1.15-1.51)	0.93 (0.69-1.26)
Central-west	1.25*** (1.15-1.36)	1.19* (1.03-1.39)	1.06 (0.76-1.48)
**Area of residence**			
Rural	1 (base)	1 (base)	1 (base)
Urban	1.91*** (1.78-2.04)	2.22*** (1.97-2.50)	1.05 (0.79-1.40)
**Average AIDS incidence rate[j]**	1.03*** (1.03-1.03)	x	x
**Average AIDS mortality rate[j]**	x	1.10*** (1.09-1.10)	x
**Average AIDS case-fatality rate[j]**	x	x	1.01*** (1.00-1.02)
**Garbage collection[l]**	1.00** (1.00-1.00)	1.00* (1.00-1.00)	0.99 (0.98-1.00)
**Per capita income[m]**	0.99*** (0.99-0.99)	0.99** (0.99-1.00)	0.99 (0.99-1.00)
**Extremely poor proportion[n]**	0.99* (0.98-0.99)	0.99 (0.98-1.00)	0.97 (0.95-1.00)
**Specialized Clinic rate[o]**	1.25** (1.07-1.45)	1.12 (0.86-1.46)	0.90 (0.46-1.77)
**Year of entry into the cohort**	yes	yes	yes
Obs.:	19,577,629	19,577,649	9,965

Notes: *** p-value <0,001; **p-valor <0,01; *p-valor <0,05. [a] Incidence Rate Ratios. [b] Confidence Interval. [c] Aged between 13 and 24. [d] Aged between 25 and 64. [e] Aged 65 or older. [f] Measured by capita expenses proportional to the baseline minimum wage (MW). Level 1 (More wealth): "1 or more". Level 2: "0.5 to 1". Level 3: "0.25 to 0.49". Level 4: "0< to 0.24". Level 5 (Lower wealth): "Nothing declared".. [g] Water supply: Others – well, spring, and others. [h] Housing Material: No – Coated clay, uncoated clay, wood, and others. [i] Lighting: Non-electric – No meter, lamp, candles and others. [j] Average rates for the period (2007-2015) by municipality. [l] % of municipality population with baseline garbage collection. [m] Baseline variable. [n] % of the extremely poor in the baseline municipality (according to the nominal minimum wage: earn up to 1/4 salary). [o] Per 1,000 inhabitants of the baseline municipality. All statistical tests used where two-sided and, where appropriate, adjustments were made for multiple comparisons.

Table S13. Estimates of the average effect of the *Programa Bolsa Família* (PBF), in unadjusted and adjusted Poisson models (with robust standard error), on AIDS incidence, mortality and case-fatality rate, 2007-2015 - Group 3 of aggregated variables at the municipal level.

Models	Outcomes (IRR[a] – IC[b] 95%)		
	Incidence	Mortality	Case-Fatality
**Unadjusted Models**			
**PBF**	0.67*** (0.65-0.69)	0.67*** (0.64-0.71)	0.74*** (0.66-0.82)
**Adjusted Model**			
**PBF**	0.60*** (0.57-0.62)	0.60*** (0.57-0.64)	0.75*** (0.66-0.85)
**Sex**			
Female	1 (base)	1 (base)	1 (base)
Male	1.21*** (1.17-1.25)	1.34*** (1.27-1.42)	1.30*** (1.15-1.46)
**Age**			
Adolescents and Youth people[c]	1 (base)	1 (base)	1 (base)
Adults[d]	2.02*** (1.93-2.10)	3.50*** (3.21-3.83)	1.66*** (1.38-2.01)
Older people[e]	0.32*** (0.28-0.36)	0.77* (0.63-0.94)	2.71*** (1.72-4.28)
**Race/ethnicity**			
White	1 (base)	1 (base)	1 (base)
Mixed-race	1.25*** (1.20-1.30)	1.25*** (1.17-1.34)	1.08 (0.92-1.26)
Black	1.67*** (1.59-1.77)	1.81*** (1.66-1.98)	1.00 (0.83-1.22)
Indigenous	1.39* (1.02-1.89)	0.95 (0.53-1.70)	0.60 (0.21-1.68)
**Education**			
More than High School	1 (base)	1 (base)	1 (base)
High school	1.03 (0.90-1.18)	1.47** (1.10-1.97)	1.90* (0.99-3.61)
Elementary school	1.40*** (1.23-1.60)	2.65*** (1.99-3.53)	2.63** (1.39-4.96)
Attended pre-school	0.89 (0.70-1.13)	1.27 (0.79-2.04)	0.97 (0.34-2.78)
Never attended school	1.27** (1.11-1.46)	2.78*** (2.07-3.74)	3.50*** (1.81-6.76)
**Wealth[f]**			
Level 1 (More wealth)	1 (base)	1 (base)	1 (base)
Level 2	1.00 (0.90-1.00)	0.92 (0.78-1.09)	1.26 (0.83-1.92)
Level 3	1.18*** (1.07-1.30)	1.23* (1.04-1.46)	1.67* (1.10-2.51)
Level 4	1.60*** (1.45-1.77)	1.59*** (1.34-1.87)	1.36 (0.90-2.05)
Level 5 (Lower wealth)	2.17*** (1.96-2.41)	2.40*** (2.01-2.85)	1.63* (1.07-2.49)
**AIDS Treatment**			
Yes	x	x	1 (base)
No	x	x	2.66*** (2.31-3.07)
**Water supply**			
Public network	1 (base)	1 (base)	1 (base)
Others[g]	1.02 (0.97-1.07)	1.08* (1.00-1.18)	1.01 (0.85-1.20)
**Housing material (Brick)**			
Yes	1 (base)	1 (base)	1 (base)
No[h]	1.24*** (1.19-1.30)	1.35*** (1.25-1.45)	1.20* (1.02-1.41)
**Lighting**			
Electricity	1 (base)	1 (base)	1 (base)
Non-electric[i]	1.32*** (1.25-1.38)	1.37*** (1.26-1.49)	1.23* (1.04-1.45)
**Region**			
North	1 (base)	1 (base)	1 (base)
Northeast	1.22*** (1.14-1.30)	1.08 (0.96-1.22)	1.04 (0.80-1.33)
Southeast	1.07* (1.00-1.15)	1.23** (1.09-1.39)	0.97 (0.73-1.29)
South	1.29*** (1.19-1.40)	1.21* (1.04-1.40)	0.88 (0.65-1.20)
Central-west	1.14** (1.05-1.24)	1.12 (0.96-1.30)	1.03 (0.75-1.43)
**Area of residence**			
Rural	1 (base)	1 (base)	1 (base)
Urban	1.90*** (1.78-2.04)	2.21*** (1.97-2.49)	1.05 (0.79-1.40)
**Average AIDS incidence rate[j]**	1.03*** (1.02-1.03)	x	x
**Average AIDS mortality rate[j]**	x	1.10*** (1.09-1.10)	x
**Average AIDS case-fatality rate[j]**	x	x	1.01*** (1.00-1.02)
**Garbage collection[l]**	1.00** (1.00-1.00)	1.00** (1.00-1.00)	0.99 (0.98-1.00)
**Gini index[m]**	0.99*** (0.98-0.99)	0.99*** (0.98-0.99)	0.99 (0.98-1.00)
**Extremely poor proportion[n]**	0.99 (0.99-1.00)	0.99 (0.98-1.00)	0.98 (0.95-1.00)
**Specialized Clinic rate[o]**	1.11 (0.96-1.29)	1.05 (0.82-1.36)	0.89 (0.47-1.66)
**Year of entry into the cohort**	yes	yes	yes
Obs.:	19,577,629	19,577,649	9,965

Notes: *** p-value <0,001; **p-valor <0,01; *p-valor <0,05. [a] Incidence Rate Ratios. [b] Confidence Interval. [c] Aged between 13 and 24. [d] Aged between 25 and 64. [e] Aged 65 or older. [f] Measured by capita expenses proportional to the baseline minimum wage (MW). Level 1 (More wealth): "1 or more". Level 2: "0.5 to 1". Level 3: "0.25 to 0.49". Level 4: "0< to 0.24". Level 5 (Lower wealth): "Nothing declared". [g] Water supply: Others – well, spring, and others. [h] Housing Material: No – Coated clay, uncoated clay, wood, and others. [i] Lighting: Non-electric – No meter, lamp, candles and others. [j] Average rates for the period (2007-2015) by municipality. [l] % of municipality population with baseline garbage collection. [m] Baseline variable. [m] % of the extremely poor in the baseline municipality (according to the nominal minimum wage: earn up to 1/4 salary). [o] Per 1,000 inhabitants of the baseline municipality. All statistical tests used where two-sided and, where appropriate, adjustments were made for multiple comparisons.

Table S14. Estimates of the average effect of the *Programa Bolsa Família* (PBF), in unadjusted and adjusted Poisson models (with robust standard error), on AIDS incidence, mortality and case-fatality rate, 2007-2015 – No endemic variables.

Models	Outcomes (IRR[a] – IC[b] 95%)		
	**Incidence**	**Mortality**	**Case-Fatality**
**Unadjusted Models**			
**PBF**	0.71*** (0.68-0.73)	0.71*** (0.67-0.75)	0.74*** (0.67-0.83)
**Adjusted Model**			
**PBF**	0.64*** (0.62-0.66)	0.65*** (0.61-0.69)	0.76*** (0.67-0.85)
**Sex**			
Female	1 (base)	1 (base)	1 (base)
Male	1.18*** (1.14-1.22)	1.30*** (1.23-1.38)	1.28*** (1.15-1.43)
**Age**			
Adolescents and Youth people[c]	1 (base)	1 (base)	1 (base)
Adults[d]	2.00*** (1.92-2.09)	3.45*** (3.16-3.77)	1.67*** (1.42-1.98)
Older people[e]	0.32*** (0.28-0.36)	0.78* (0.64-0.96)	2.33*** (1.53-3.56)
**Race/ethnicity**			
White	1 (base)	1 (base)	1 (base)
Mixed-race	1.23*** (1.18-1.28)	1.22*** (1.14-1.30)	1.04 (0.91-1.20)
Black	1.76*** (1.66-1.85)	1.88*** (1.73-2.06)	0.98 (0.81-1.18)
Indigenous	1.42* (1.05-1.93)	0.94 (0.52-1.71)	1.06 (0.43-2.59)
**Education**			
More than High School	1 (base)	1 (base)	1 (base)
High school	1.05 (0.92-1.20)	1.47** (1.09-1.97)	1.93* (1.09-3.44)
Elementary school	1.41*** (1.24-1.61)	2.63*** (1.97-3.51)	2.85*** (1.62-5.02)
Attended pre-school	0.90 (0.71-1.14)	1.20 (0.75-1.92)	1.55 (0.61-3.90)
Never attended school	1.25*** (1.09-1.44)	2.68*** (1.99-3.62)	3.76*** (2.09-6.76)
**Wealth[f]**			
Level 1 (More wealth)	1 (base)	1 (base)	1 (base)
Level 2	0.98 (0.89-1.08)	0.90 (0.75-1.07)	1.12 (0.75-1.66)
Level 3	1.17** (1.06-1.29)	1.22* (1.03-1.45)	1.50* (1.02-2.20)
Level 4	1.58*** (1.43-1.74)	1.56*** (1.32-1.86)	1.26 (0.86-1.84)
Level 5 (Lower wealth)	2.13*** (1.92-2.36)	2.36*** (1.97-2.83)	1.47 (0.99-2.18)
**AIDS Treatment**			
Yes	x	x	1 (base)
No	x	x	2.72*** (2.39-3.09)
**Water supply**			
Public network	1 (base)	1 (base)	1 (base)
Others[g]	1.01 (0.96-1.06)	1.07 (0.98-1.16)	1.03 (0.88-1.20)
**Housing material (Brick)**			
Yes	1 (base)	1 (base)	1 (base)
No[h]	1.16*** (1.11-1.22)	1.25*** (1.16-1.35)	1.18* (1.01-1.37)
**Lighting**			
Electricity	1 (base)	1 (base)	1 (base)
Non-electric[i]	1.60*** (1.53-1.68)	1.67*** (1.54-1.81)	1.16 (0.99-1.36)
**Region**			
North	1 (base)	1 (base)	1 (base)
Northeast	1.84*** (0.74-0.90)	0.79*** (0.71-0.89)	1.02 (0.81-1.28)
Southeast	0.86*** (0.80-0.92)	1.27*** (1.13-1.43)	1.23 (0.96-1.57)
South	2.25*** (2.08-2.44)	2.55*** (2.23-2.92)	1.05 (0.79-1.38)
Central-west	0.97 (0.89-1.06)	1.14 (0.97-1.33)	1.33 (0.99-1.80)
**Area of residence**			
Rural	1 (base)	1 (base)	1 (base)
Urban	2.25*** (2.11-2.41)	2.60*** (2.32-2.92)	1.10 (0.88-1.37)
**Inadequate sanitation[j]**	0.99*** (0.99-0.99)	0.99*** (0.99-0.99)	1.00 (0.99-1.00)
**Unemployment rate (%)[l]**	1.05*** (1.04-1.06)	1.06*** (1.05-1.07)	1.00 (0.98-1.02)
**Doctors per 1000 inhabitants[m]**	1.22*** (1.20-1.25)	1.21*** (1.16-1.25)	0.85** (0.78-0.94)
**Nurses per 1000 inhabitants[m]**	1.05 (0.97-1.14)	1.08*** (0.94-1.24)	1.52** (1.14-2.02)
**Hospital beds per 1000 inhabitants[m]**	0.99 (0.98-1.00)	0.99 (0.99-1.00)	0.99 (0.96-1.03)
**Year of entry into the cohort**	yes	yes	yes
Obs.:	19,577,629	19,577,649	11,477

Notes: *** p-value <0,001; **p-valor <0,01; *p-valor <0,05. [a] Incidence Rate Ratios. [b] Confidence Interval. [c] Aged between 13 and 24. [d] Aged between 25 and 64. [e] Aged 65 or older. [f] Measured by capita expenses proportional to the baseline minimum wage (MW). Level 1 (More wealth): "1 or more". Level 2: "0.5 to 1". Level 3: "0.25 to 0.49". Level 4: "0< to 0.24". Level 5 (Lower wealth): "Nothing declared". [g] Water supply: Others – well, spring, and others. [h] Housing Material: No – Coated clay, uncoated clay, wood, and others. [i] Lighting: Non-electric – No meter, lamp, candle and others. [j] % of the municipality's population with inadequate baseline sanitation. [l] Baseline municipality unemployment rate. [m] Per 1,000 inhabitants of the baseline municipality. All statistical tests used where two-sided and, where appropriate, adjustments were made for multiple comparisons.

Table S15. Estimates of the average effect of the *Programa Bolsa Família* (PBF), in unadjusted and adjusted Poisson models (with robust standard error), on AIDS incidence, mortality and case-fatality rate in Brazil, 2007-2015 - Only with the endemic variables at the municipal level.

Models	Outcomes (IRR[a] – IC[b] 95%)		
	Incidence	Mortality	Case-Fatality
**Unadjusted Models**			
**PBF**	0.66*** (0.64-0.68)	0.67*** (0.63-0.71)	0.74*** (0.66-0.83)
**Adjusted Model**			
**PBF**	0.59*** (0.57-0.62)	0.60*** (0.57-0.64)	0.75*** (0.66-0.85)
**Sex**			
Female	1 (base)	1 (base)	1 (base)
Male	1.20*** (1.16-1.24)	1.34*** (1.26-1.41)	1.28*** (1.14-1.44)
**Age**			
Adolescents and Youth people[c]	1 (base)	1 (base)	1 (base)
Adults[d]	2.02*** (1.93-2.11)	3.55*** (3.25-3.87)	1.68*** (1.40-2.02)
Older people[e]	0.32*** (0.27-0.36)	0.77* (0.63-0.95)	2.79*** (1.74-4.50)
**Race/ethnicity**			
White	1 (base)	1 (base)	1 (base)
Mixed-race	1.24*** (1.19-1.29)	1.24*** (1.16-1.33)	1.08 (0.92-1.26)
Black	1.66*** (1.57-1.75)	1.81*** (1.66-1.98)	1.00 (0.83-1.22)
Indigenous	1.32* (0.97-1.79)	0.88 (0.49-1.56)	0.58 (0.20-1.62)
**Education**			
More than High School	1 (base)	1 (base)	1 (base)
High school	1.04 (0.91-1.19)	1.51** (1.13-2.01)	1.98* (1.09-3.59)
Elementary school	1.42*** (1.25-1.62)	2.74*** (2.06-3.63)	2.82*** (1.57-5.05)
Attended pre-school	0.90 (0.71-1.14)	1.27 (0.80-2.03)	1.06 (0.38-2.90)
Never attended school	1.28** (1.11-1.47)	2.82*** (2.10-3.78)	3.75*** (2.04-6.90)
**Wealth[f]**			
Level 1 (More wealth)	1 (base)	1 (base)	1 (base)
Level 2	0.99 (0.89-1.09)	0.92 (0.78-1.09)	1.25 (0.82-1.91)
Level 3	1.16** (1.06-1.28)	1.21* (1.02-1.43)	1.63* (1.08-2.45)
Level 4	1.59*** (1.44-1.75)	1.58*** (1.34-1.86)	1.33 (0.88-2.00)
Level 5 (Lower wealth)	2.13*** (1.92-2.35)	2.33*** (1.96-2.78)	1.58* (1.03-2.41)
**AIDS Treatment**			
Yes	x	x	1 (base)
No	x	x	2.61*** (2.27-3.00)
**Water supply**			
Public network	1 (base)	1 (base)	1 (base)
Others[g]	1.02 (0.97-1.07)	1.08* (1.00-1.18)	1.02 (0.86-1.21)
**Housing material (Brick)**			
Yes	1 (base)	1 (base)	1 (base)
No[h]	1.21*** (1.16-1.27)	1.31*** (1.21-1.41)	1.20* (1.02-1.41)
**Lighting**			
Electricity	1 (base)	1 (base)	1 (base)
Non-electric[i]	1.29*** (1.23-1.36)	1.34*** (1.24-1.46)	1.20* (1.02-1.42)
**Region**			
North	1 (base)	1 (base)	1 (base)
Northeast	1.21*** (1.14-1.29)	1.08 (0.97-1.21)	1.02 (0.79-1.30)
Southeast	1.20*** (1.12-1.28)	1.43*** (1.28-1.60)	1.02 (0.79-1.32)
South	1.46*** (1.36-1.58)	1.39*** (1.21-1.59)	0.93 (0.71-1.23)
Central-west	1.24*** (1.14-1.34)	1.12** (1.07-1.43)	1.12 (0.82-1.52)
**Area of residence**			
Rural	1 (base)	1 (base)	1 (base)
Urban	2.04*** (1.90-2.18)	2.45*** (2.18-2.75)	1.01 (0.76-1.35)
**Average AIDS incidence rate[j]**	1.03*** (1.02-1.03)	x	x
**Average AIDS mortality rate[j]**	x	1.10*** (1.09-1.10)	x
**Average AIDS case-fatality rate[j]**	x	x	1.01*** (1.01-1.02)
**Year of entry into the cohort**	yes	yes	yes
Obs.:	19,631,708	19,631,728	9,976

Notes: *** p-value <0,001; **p-valor <0,01; *p-valor <0,05. [a] Incidence Rate Ratios. [b] Confidence Interval. [c] Aged between 13 and 24. [d] Aged between 25 and 64. [e] Aged 65 or older. [f] Measured by capita expenses proportional to the baseline minimum wage (MW). Level 1 (More wealth): "1 or more". Level 2: "0.5 to 1". Level 3: "0.25 to 0.49". Level 4: "0< to 0.24". Level 5 (Lower wealth): "Nothing declared". [g] Water supply: Other – well, spring, and others. [h] Housing Material: No – Coated clay, uncoated clay, wood, and others. [i] Lighting: Non-electric – No meter, lamps, candles, and others. [j] Average rates for the period (2007-2015) by municipality. All statistical tests used where two-sided and, where appropriate, adjustments were made for multiple comparisons.

Table S16. Estimates of the average effect of the *Programa Bolsa Família* (PBF), in unadjusted and adjusted Poisson models (with robust standard error), on AIDS incidence, mortality and case-fatality rate, 2007-2015 – Without the aggregated variables at the municipal level.

Models	Outcomes (IRR[a] – IC[b] 95%)		
	**Incidence**	**Mortality**	**Case-Fatality**
**Unadjusted Models**			
**PBF**	0.74*** (0.72-0.77)	0.75*** (0.71-0.79)	0.74*** (0.67-0.83)
**Adjusted Models**			
**PBF**	0.67*** (0.64-0.69)	0.68*** (0.64-0.72)	0.76*** (0.67-0.85)
**Sex**			
Female	1 (base)	1 (base)	1 (base)
Male	1.15*** (1.12-1.19)	1.25*** (1.19-1.32)	1.29*** (1.15-1.44)
**Age**			
Adolescents and Youth people[c]	1 (base)	1 (base)	1 (base)
Adults[d]	2.01*** (1.93-2.10)	3.47*** (3.18-3.78)	1.68*** (1.42-1.98)
Older people[e]	0.31*** (0.27-0.35)	0.76* (0.62-0.93)	2.35*** (1.53-3.61)
**Race/ethnicity**			
White	1 (base)	1 (base)	1 (base)
Mixed-race	1.25*** (1.21-1.30)	1.26*** (1.18-1.35)	1.05 (0.92-1.21)
Black	1.93*** (1.83-2.03)	2.09*** (1.92-2.28)	0.98 (0.81-1.17)
Indigenous	1.38* (1.02-1.85)	0.96 (0.55-1.67)	1.05 (0.43-2.54)
**Education**			
More than High School	1 (base)	1 (base)	1 (base)
High school	1.03 (0.90-1.18)	1.51** (1.13-2.01)	2.02* (1.15-3.55)
Elementary school	1.35*** (1.18-1.54)	2.63*** (1.98-3.48)	2.98*** (1.71-5.18)
Attended pre-school	0.83 (0.66-1.05)	1.21 (0.73-1.93)	1.56 (0.63-3.88)
Never attended school	1.14 (0.99-1.31)	2.54*** (1.90-3.41)	4.00*** (2.25-7.11)
**Wealth[f]**			
Level 1 (More wealth)	1 (base)	1 (base)	1 (base)
Level 2	0.98 (0.89-1.08)	0.91 (0.77-1.08)	1.14 (0.77-1.69)
Level 3	1.17** (1.06-1.29)	1.23* (1.04-1.45)	1.51* (1.04-2.21)
Level 4	1.58*** (1.43-1.74)	1.60*** (1.35-1.89)	1.28 (0.88-1.87)
Level 5 (Lower wealth)	1.98*** (1.79-2.19)	2.25*** (1.89-2.69)	1.52* (1.02-2.24)
**AIDS Treatment**			
Yes	x	x	1 (base)
No	x	x	2.68*** (2.35-3.04)
**Water supply**			
Public network	1 (base)	1 (base)	1 (base)
Others[g]	0.97 (0.92-1.02)	1.03* (0.95-1.11)	1.02 (0.87-1.19)
**Housing material (Brick)**			
Yes	1 (base)	1 (base)	1 (base)
No[h]	1.06* (1.01-1.11)	1.15*** (1.06-1.23)	1.20* (1.03-1.39)
**Lighting**			
Electricity	1 (base)	1 (base)	1 (base)
Non-electric[i]	1.78*** (1.69-1.86)	1.88*** (1.74-2.03)	1.16 (0.99-1.35)
**Region**			
North	1 (base)	1 (base)	1 (base)
Northeast	0.98 (0.92-1.04)	0.94 (0.84-1.05)	1.01 (0.81-1.26)
Southeast	1.06 (0.99-1.13)	1.54*** (1.37-1.72)	1.11 (0.88-1.40)
South	2.40*** (2.24-2.57)	2.58*** (2.29-2.91)	0.95 (0.74-1.21)
Central-west	1.03 (0.95-1.12)	1.15* (1.00-1.33)	1.22 (0.93-1.60)
**Area of residence**			
Rural	1 (base)	1 (base)	1 (base)
Urban	2.95*** (2.77-3.15)	3.45*** (3.08-3.86)	1.03 (0.84-1.27)
**Year of entry into the cohort**	yes	yes	yes
Obs.:	19,634,193	19,634,213	11,495

Notes: *** p-value <0,001; **p-valor <0,01; *p-valor <0,05. [a] Incidence Rate Ratios. [b] Confidence Interval. [c] Aged between 13 and 24. [d] Aged between 25 and 64. [e] Aged 65 or older. [f] Measured by capita expenses proportional to the baseline minimum wage (MW). Level 1 (More wealth): "1 or more". Level 2: "0.5 to 1". Level 3: "0.25 to 0.49". Level 4: "0< to 0.24". Level 5 (Lower wealth): "Nothing declared". [g] Water supply: Other – well, spring, and others. [h] Housing Material: No – Coated clay, uncoated clay, wood, and others. [i] Lighting: Non-electric – No meter, lamps, candles, and others. All statistical tests used where two-sided and, where appropriate, adjustments were made for multiple comparisons.

Table S17. Estimates of the average effect of the *Programa Bolsa Família* (PBF), in unadjusted and adjusted Poisson models (with robust standard error), on AIDS incidence, mortality and case-fatality rate, 2007-2015 - With per capita income.

Models	Outcomes (IRR[a] – IC[b] 95%)		
	Incidence	Mortality	Case-Fatality
**Unadjusted Models**			
**PBF**	0.66*** (0.64-0.69)	0.71*** (0.67-0.75)	0.75*** (0.67-0.85)
**Adjusted Models**			
**PBF**	0.56*** (0.54-0.58)	0.60*** (0.57-0.64)	0.78*** (0.68-0.90)
**Sex**			
Female	1 (base)	1 (base)	1 (base)
Male	1.19*** (1.15-1.23)	1.34*** (1.26-1.42)	1.28*** (1.13-1.45)
**Age**			
Adolescents and Youth people[c]	1 (base)	1 (base)	1 (base)
Adults[d]	2.02*** (1.93-2.11)	3.36*** (3.05-3.70)	1.67*** (1.37-2.04)
Older people[e]	0.32*** (0.28-0.37)	0.72** (0.58-0.88)	2.78*** (1.73-4.47)
**Race/ethnicity**			
White	1 (base)	1 (base)	1 (base)
Mixed-race	1.25*** (1.20-1.31)	1.25*** (1.16-1.35)	1.08 (0.92-1.27)
Black	1.69*** (1.60-1.80)	1.84*** (1.68-2.02)	1.02 (0.84-1.25)
Indigenous	1.49* (1.07-2.06)	0.80 (0.40-1.57)	0.57 (0.18-1.47)
**Education**			
More than High School	1 (base)	1 (base)	1 (base)
High school	1.10 (0.96-1.26)	1.50** (1.13-2.01)	2.29* (1.32-3.96)
Elementary school	1.52*** (1.33-1.73)	2.73*** (2.06-3.60)	3.19*** (1.87-5.43)
Attended pre-school	0.95 (0.75-1.21)	1.18 (0.74-1.88)	1.33 (0.52-3.41)
Never attended school	1.41*** (1.22-1.62)	2.85*** (2.13-3.81)	3.86*** (2.20-6.77)
**Per capita income**			
> ½ of minimum wage	1 (base)	1 (base)	1 (base)
¼ < and ≤ ½ of minimum wage	0.97 (0.89-1.06)	0.86 (0.73-1.00)	0.75 (0.52-1.07)
0< and ≤ ¼ of minimum wage	1.18*** (1.09-1.27)	0.92 (0.81-1.06)	0.76 (0.55-1.04)
Nothing declared	2.02*** (1.85-2.20)	1.75*** (1.51-2.03)	0.87 (0.61-1.23)
**AIDS Treatment**			
Yes	x	x	1 (base)
No	x	x	2.76*** (2.39-3.19)
**Water supply**			
Public network	1 (base)	1 (base)	1 (base)
Others[f]	1.00 (0.95-1.05)	1.05 (0.97-1.15)	1.08 (0.90-1.29)
**Housing material (Brick)**			
Yes	1 (base)	1 (base)	1 (base)
No [g]	1.22*** (1.16-1.28)	1.31*** (1.21-1.42)	1.17 (0.99-1.38)
**Lighting**			
Electricity	1 (base)	1 (base)	1 (base)
Non-electric [h]	1.38*** (1.31-1.46)	1.46*** (1.34-1.60)	1.28** (1.07-1.52)
**Region**			
North	1 (base)	1 (base)	1 (base)
Northeast	1.12** (1.04-1.20)	0.98 (0.87-1.11)	1.01 (0.77-1.33)
Southeast	1.11** (1.03-1.19)	1.25*** (1.10-1.41)	1.00 (0.74-1.35)
South	1.41*** (1.29-1.53)	1.27** (1.09-1.48)	0.94 (0.67-1.31)
Central-west	1.12* (1.02-1.22)	1.04 (0.88-1.22)	1.08 (0.76-1.55)
**Area of residence**			
Rural	1 (base)	1 (base)	1 (base)
Urban	1.95*** (1.82-2.10)	2.32*** (2.06-2.62)	1.16 (0.86-1.56)
**Average AIDS incidence rate[i]**	1.03*** (1.02-1.03)	x	x
**Average AIDS mortality rate[i]**	x	1.10*** (1.09-1.11)	x
**Average AIDS case-fatality rate[i]**	x	x	1.01*** (1.00-1.02)
**Inadequate sanitation [j]**	0.99*** (0.99-0.99)	0.99*** (0.98-0.99)	1.00 (0.99-1.01)
**Unemployment rate (%)[l]**	1.02*** (1.01-1.02)	1.02*** (1.01-1.03)	0.98 (0.95-1.01)
**Doctors per 1000 inhabitants[m]**	1.02 (0.99-1.05)	1.05* (1.00-1.10)	0.84* (0.75-0.94)
**Nurses per 1000 inhabitants[m]**	0.82*** (0.74-0.90)	0.79*** (0.68-0.92)	1.71* (1.22-2.40)
**Hospital beds per 1000 inhabitants[m]**	1.00 (0.98-1.01)	0.99 (0.99-1.00)	0.98 (0.94-1.02)
**Year of entry of individual in cohort**	yes	yes	yes
Obs.:	19,537,673	19,537,693	9,929

Notes: *** p-value <0,001; **p-valor <0,01; *p-valor <0,05. [a] Incidence Rate Ratios. [b] Confidence Interval. [c] Aged between 13 and 24. [d] Aged between 25 and 64. [e] Aged 65 or older. [f] Water supply: Others – well, spring, and others. [g] Housing Material: No – Coated clay, uncoated clay, wood, and others. [h] Lighting: Non-electric – No meter, lamp, candle and others. [i] Average rates for the period (2007-2015) by municipality. [j] % of the municipality's population with inadequate baseline sanitation. [l] Baseline municipality unemployment rate. [m] Per 1,000 inhabitants of the baseline municipality. All statistical tests used where two-sided and, where appropriate, adjustments were made for multiple comparisons.

Table S18. Estimates of the average effect of the *Programa Bolsa Família* (PBF), in unadjusted and adjusted Poisson models (with robust standard error), on AIDS incidence, mortality and case-fatality rate in Brazil, 2007-2015 – Without IPTW.

Models	Outcomes (IRR[a] – IC[b] 95%)		
	Incidence	Mortality	Case-Fatality
**Unadjusted Models**			
**PBF**	0.83*** (0.81-0.86)	0.86*** (0.82-0.90)	0.74*** (0.64-0.81)
**Adjusted Models**			
**PBF**	0.58*** (0.56-0.60)	0.59*** (0.57-0.62)	0.77*** (0.68-0.86)
**Sex**			
Female	1 (base)	1 (base)	1 (base)
Male	1.21*** (1.18-1.25)	1.34*** (1.28-1.41)	1.32*** (1.18-1.47)
**Age**			
Adolescents and Youth people[c]	1 (base)	1 (base)	1 (base)
Adults[d]	2.03*** (1.95-2.11)	3.56*** (3.29-3.85)	1.73*** (1.47-2.04)
Older people[e]	0.30*** (0.27-0.34)	0.72*** (0.61-0.86)	2.83*** (1.92-4.15)
**Race/ethnicity**			
White	1 (base)	1 (base)	1 (base)
Mixed-race	1.21*** (1.17-1.26)	1.22*** (1.15-1.30)	1.06 (0.93-1.22)
Black	1.66*** (1.59-1.75)	1.83*** (1.69-1.98)	1.05 (0.88-1.25)
Indigenous	1.05 (0.84-1.33)	0.98 (0.64-1.49)	0.59 (0.20-1.68)
**Education**			
More than High School	1 (base)	1 (base)	1 (base)
High school	1.05 (0.93-1.18)	1.40* (1.07-1.83)	1.65* (1.01-2.70)
Elementary school	1.41*** (1.26-1.59)	2.53*** (1.95-3.29)	2.27** (1.40-3.68)
Attended pre-school	0.95 (0.76-1.18)	1.29 (0.84-1.99)	1.21 (0.49-3.01)
Never attended school	1.32*** (1.16-1.50)	2.71*** (2.07-3.56)	2.71*** (1.62-4.51)
**Wealth[f]**			
Level 1 (More wealth)	1 (base)	1 (base)	1 (base)
Level 2	1.04 (0.96-1.13)	1.01 (0.88-1.17)	1.40* (1.02-1.91)
Level 3	1.31*** (1.21-1.43)	1.38*** (1.19-1.60)	1.74** (1.27-2.38)
Level 4	1.75*** (1.61-1.90)	1.78*** (1.54-2.05)	1.46* (1.07-1.99)
Level 5 (Lower wealth)	2.39*** (2.19-2.61)	2.67*** (2.29-3.11)	1.67** (1.21-2.32)
**AIDS Treatment**			
Yes	x	x	1 (base)
No	x	x	2.65*** (2.34-3.00)
**Water supply**			
Public network	1 (base)	1 (base)	1 (base)
Others[g]	1.00 (0.96-1.05)	1.06 (0.98-1.14)	1.04 (0.89-1.22)
**Housing material (Brick)**			
Yes	1 (base)	1 (base)	1 (base)
No [h]	1.25*** (1.20-1.30)	1.32*** (1.23-1.42)	1.11 (0.84-1.29)
**Lighting**			
Electricity	1 (base)	1 (base)	1 (base)
Non-electric [i]	1.27*** (1.21-1.32)	1.33*** (1.24-1.43)	1.21* (1.04-1.40)
**Region**			
North	1 (base)	1 (base)	1 (base)
Northeast	1.23*** (1.16-1.31)	1.07 (0.96-1.19)	1.03 (0.81-1.31)
Southeast	1.22*** (1.15-1.30)	1.43*** (1.28-1.59)	1.08 (0.83-1.40)
South	1.53*** (1.42-1.64)	1.41*** (1.24-1.62)	0.97 (0.73-1.29)
Central-west	1.24*** (1.15-1.35)	1.17* (1.01-1.39)	1.22 (0.89-1.68)
**Area of residence**			
Rural	1 (base)	1 (base)	1 (base)
Urban	1.93*** (1.81-2.05)	2.23*** (2.00-2.49)	1.06 (0.82-1.38)
**Average AIDS incidence rate[j]**	1.03*** (1.02-1.03)	x	x
**Average AIDS mortality rate[j]**	x	1.10*** (1.09-1.10)	x
**Average AIDS case-fatality rate[j]**	x	x	1.01*** (1.01-1.02)
**Inadequate sanitation [l]**	0.99*** (0.99-0.99)	0.99*** (0.99-0.99)	1.00 (0.99-1.01)
**Unemployment rate (%)[m]**	1.01*** (1.00-1.02)	1.02*** (1.01-1.03)	0.99 (0.97-1.01)
**Doctors per 1000 inhabitants[n]**	1.02* (1.00-1.05)	1.04* (1.00-1.09)	0.84** (0.76-0.93)
**Nurses per 1000 inhabitants[n]**	0.84*** (0.78-0.91)	0.82** (0.71-0.93)	1.73*** (1.30-2.31)
**Hospital beds per 1000 inhabitants[n]**	0.99 (0.98-1.00)	0.99 (0.99-1.00)	0.98 (0.94-1.02)
**Year of entry into the cohort**	yes	yes	yes
Obs.:	19,577,629	19,577,649	9,965

Notes: *** p-value <0,001; **p-valor <0,01; *p-valor <0,05. [a] Incidence Rate Ratios. [b] Confidence Interval. [c] Aged between 13 and 24. [d] Aged between 25 and 64. [e] Aged 65 or older. [f] Measured by capita expenses proportional to the baseline minimum wage (MW). Level 1 (More wealth): "1 or more". Level 2: "0.5 to 1". Level 3: "0.25 to 0.49". Lel 4: "0< to 0.24". Level 5 (Lower wealth): "Nothing declared". [g] Water supply: Other – well, spring, and others. [h] Housing Material: No – Coated clay, uncoated clay, wood, and others. [i] Lighting: Non-electric – No meter, lamps, candles, and others. [j] Average rates for the period (2007-2015) by municipality. [l] % of the municipality's population with inadequate baseline sanitation. [m] Baseline municipality unemployment rate. [n] Per 1,000 inhabitants of the baseline municipality. All statistical tests used where two-sided and, where appropriate, adjustments were made for multiple comparisons.

Table S19. Estimates of the average effect of the *Programa Bolsa Família* (PBF), in unadjusted and adjusted Poisson models, on AIDS incidence, mortality and case-fatality rate, 2007-2015 (without robust cluster standard errors at the individual level).

Models	Outcomes (IRR[1] – IC[2] 95%)		
	**Incidence**	**Mortality**	**Case-Fatality**
**Unadjusted Model**			
**PBF**	0.66*** (0.64-0.69)	0.67*** (0.63-0.71)	0.74*** (0.66-0.83)
**Adjusted Model**			
**PBF**	0.59*** (0.57-0.61)	0.60*** (0.57-0.64)	0.75*** (0.66-0.85)
**Sex**			
Female	1 (base)	1 (base)	1 (base)
Male	1.21*** (1.17-1.25)	1.34*** (1.27-1.42)	1.27*** (1.13-1.43)
**Age**			
Adolescents and young people[c]	1 (base)	1 (base)	1 (base)
Adults[d]	2.02*** (1.93-2.10)	3.51*** (3.22-3.84)	1.69*** (1.40-2.04)
Older people[e]	0.32*** (0.28-0.37)	0.78* (0.63-0.95)	2.84*** (1.78-2.04)
**Race/ethnicity**			
White	1 (base)	1 (base)	1 (base)
Mixed-race	1.24*** (1.19-1.29)	1.24*** (1.15-1.32)	1.07 (0.92-1.25)
Black	1.66*** (1.57-1.75)	1.79*** (1.64-1.95)	1.01 (0.83-1.22)
Indigenous	1.36* (1.00-1.85)	0.93 (0.51-1.66)	0.57 (0.20-1.61)
**Education**			
More than high school	1 (base)	1 (base)	1 (base)
High school	1.04 (0.91-1.18)	1.48** (1.10-1.97)	1.95* (1.06-3.58)
Elementary school	1.40*** (1.23-1.60)	2.68*** (2.02-3.56)	2.77** (1.53-5.02)
Attended pre-school	0.91 (0.72-1.15)	1.29 (0.80-2.05)	1.00 (0.35-2.78)
Never attended school	1.28*** (1.11-1.47)	2.83*** (2.11-3.79)	3.62*** (1.95-6.74)
**Wealth[f]**			
Level 1 (More wealth)	1 (base)	1 (base)	1 (base)
Level 2	0.98 (0.89-1.08)	0.91 (077-1.08)	1.22 (0.80-1.87)
Level 3	1.16* (1.05-1.28)	1.20* (1.02-1.42)	1.61* (1.06-2.43)
Level 4	1.57*** (1.42-1.73)	1.55*** (1.31-1.83)	1.31 (0.87-1.98)
Level 5 (Lower wealth)	2.13*** (1.93-2.36)	2.34*** (1.97-2.79)	1.56* (1.02-2.40)
**AIDS treatment**			
Yes	x	x	1 (base)
No	x	x	2.67*** (2.32-3.08)
**Water supply**			
Public network	1 (base)	1 (base)	1 (base)
Other[g]	1.01 (0.96-1.06)	1.07 (0.98-1.16)	1.03 (0.87-1.23)
**Housing material (brick)**			
Yes	1 (base)	1 (base)	1 (base)
No[h]	1.24*** (1.19-1.30)	1.35*** (1.25-1.45)	1.18* (1.00-1.39)
**Lighting**			
Electricity	1 (base)	1 (base)	1 (base)
Non-electric[i]	1.31*** (1.25-1.38)	1.36*** (1.26-1.48)	1.21* (1.02-1.43)
**Region**			
North	1 (base)	1 (base)	1 (base)
Northeast	1.19*** (1.11-1.26)	1.03 (0.92-1.160	1.06 (0.81-1.37)
Southeast	1.19*** (1.11-1.27)	1.37*** (1.22-1.54)	1.10 (0.83-1.45)
South	1.52*** (1.40-1.65)	1.43*** (1.24-1.64)	0.95 (0.70-1.30)
Central-west	1.22*** (1.12-1.34)	1.20* (1.03-1.40)	1.20 (0.85-1.68)
**Area of residence**			
Rural	1 (base)	1 (base)	1 (base)
Urban	1.89*** (1.77-2.03)	2.21*** (1.96-2.48)	1.06 (0.79-1.42)
**Average AIDS incidence rate[j]**	1.03*** (1.02-1.03)	x	x
**Average AIDS mortality rate[j]**	x	1.10*** (1.09-1.10)	x
**Average AIDS case-fatality rate[j]**	x	x	1.01*** (1.00-1.02)
**Inadequate sanitation[l]**	0.99*** (0.99-0.99)	0.99*** (0.98-0.99)	1.00 (0.99-1.01)
**Unemployment rate (%)[m]**	1.01*** (1.01-1.02)	1.02*** (1.01-1.03)	0.98 (0.96-1.01)
**Doctors per 1,000 inhabitants[n]**	1.02 (0.99-1.05)	1.05* (1.00-1.10)	0.85** (0.76-0.95)
**Nurses per 1,000 inhabitants[n]**	0.85*** (0.78-0.92)	0.80*** (0.69-0.93)	1.71** (1.23-2.36)
**Hospital beds per 1,000 inhabitants[n]**	0.99 (0.98-1.01)	0.99 (0.98-1.01)	0.97 (0.92-1.01)
**Year of entry into the cohort**	yes	yes	yes
Obs.:	19,577,629	19,577,649	9,965

Notes: *** p-value <0,001; **p-valor <0,01; *p-valor <0,05. [a] Incidence Rate Ratios. [b] Confidence Interval. [c] Aged between 13 and 24. [d] Aged between 25 and 64. [e] Aged 65 or older. [f] Measured by capita expenses proportional to the baseline minimum wage (MW). Level 1 (More wealth): "1 or more". Level 2: "0.5 to 1". Level 3: "0.25 to 0.49". Level 4: "0< to 0.24". Level 5 (Lower wealth): "Nothing declared". [g] Water supply: Other – well, spring, and others. [h] Housing Material: No – Coated clay, uncoated clay, wood, and others. [i] Lighting: Non-electric – No meter, lamps, candles, and others. [j] Average rates for the period (2007-2015) by municipality. [l] % of the municipal population with inadequate baseline sanitation. [m] Baseline municipal unemployment rate. [n] Per 1,000 inhabitants of the baseline municipality. All statistical tests used where two-sided and, where appropriate, adjustments were made for multiple comparisons.

Table S20. Estimates of the average effect of the *Programa Bolsa Famlília* (PBF) adjusted Poisson model (with robust standard errors) on AIDS incidence, mortality, and the case-fatality rate, 2007-2015 - with municipalities with adequate vital information.

Adjusted Model	Outcomes (IRR[a] – CI[b] 95%)		
	Incidence	Mortality	Case-Fatality
**PBF**	0.60*** (0.57-0.62)	0.61*** (0.57-0.65)	0.75*** (0.65-0.85)
**Sex**			
Female	1 (base)	1 (base)	1 (base)
Male	1.24*** (1.20-1.28)	1.38*** (1.30-1.47)	1.28*** (1.13-1.44)
**Age**			
Adolescents and young people[c]	1 (base)	1 (base)	1 (base)
Adults[d]	2.04*** (1.95-2.13)	3.61*** (3.28-3.97)	1.67*** (1.38-2.01)
Older people[e]	0.34*** (0.29-0.39)	0.84 (0.67-1.05)	2.75*** (1.71-4.42)
**Race/ethnicity**			
White	1 (base)	1 (base)	1 (base)
Mixed-race	1.24*** (1.19-1.30)	1.23*** (1.14-1.32)	1.08 (0.92-1.27)
Black	1.66*** (1.57-1.76)	1.80*** (1.64-1.97)	1.02 (0.84-1.24)
Indigenous	1.24 (0.87-1.78)	0.86 (0.43-1.75)	0.68 (0.24-1.90)
**Education**			
More than high school	1 (base)	1 (base)	1 (base)
High school	1.02 (0.88-1.17)	1.49* (1.09-2.03)	1.94* (1.06-3.58)
Elementary school	1.37*** (1.19-1.56)	2.61*** (1.93-3.55)	2.67** (1.46-4.87)
Attended pre-school	0.89 (0.69-1.15)	1.15 (0.68-1.92)	0.88 (0.30-2.61)
Never attended school	1.24** (1.07-1.44)	2.73*** (1.99-3.75)	3.52*** (1.87-6.59)
**Wealth[f]**			
Level 1 (More wealth)	1 (base)	1 (base)	1 (base)
Level 2	0.96 (0.87-1.06)	0.92 (0.77-1.10)	1.16 (0.75-1.78)
Level 3	1.14* (1.03-1.26)	1.25** (1.04-1.49)	1.55* (1.02-2.36)
Level 4	1.54*** (1.39-1.70)	1.54*** (1.29-1.84)	1.28 (0.84-1.95)
Level 5 (Lower wealth)	2.13*** (1.91-2.36)	2.42*** (2.01-2.91)	1.53 (0.99-2.36)
**AIDS treatment**			
Yes	x	x	1 (base)
No	x	x	2.59*** (2.24-2.99)
**Water supply**			
Public network	1 (base)	1 (base)	1 (base)
Other[g]	1.01 (0.96-1.07)	1.06 (0.97-1.16)	1.06 (0.88-1.26)
**Housing material (brick)**			
Yes	1 (base)	1 (base)	1 (base)
No[h]	1.26*** (1.20-1.33)	1.38*** (1.27-1.50)	1.22* (1.03-1.44)
**Lighting**			
Electricity	1 (base)	1 (base)	1 (base)
Non-electric[i]	1.31*** (1.25-1.38)	1.39*** (1.27-1.51)	1.21* (1.02-1.44)
**Region**			
North	1 (base)	1 (base)	1 (base)
Northeast	1.23*** (1.14-1.32)	1.08 (0.95-1.22)	1.09 (0.83-1.42)
Southeast	1.23*** (1.14-1.32)	1.42*** (1.25-1.62)	1.08 (0.81-1.45)
South	1.59*** (1.45-1.73)	1.43*** (1.22-1.67)	0.93 (0.68-1.28)
Central-west	1.26*** (1.15-1.40)	1.20* (1.01-1.42)	1.07 (0.75-1.53)
**Area of residence**			
Rural	1 (base)	1 (base)	1 (base)
Urban	1.88*** (1.74-2.04)	2.19*** (1.90-2.51)	1.16 (0.83-1.60)
**Mun. average AIDS incidence rate[j]**	1.03*** (1.02-1.03)	x	x
**Mun. average AIDS mortality rate[j]**	x	1.10*** (1.09-1.10)	x
**Mun. average AIDS case-fatality rate[j]**	x	x	1.01*** (1.00-1.02)
**Inadequate sanitation[l]**	0.99*** (0.99-0.99)	0.99*** (0.98-0.99)	1.00 (0.99-1.01)
**Unemployment rate (%)[m]**	1.01*** (1.01-1.02)	1.02*** (1.01-1.03)	0.98 (0.95-1.01)
**Doctors per 1,000 inhabitants[n]**	1.03* (1.00-1.06)	1.06* (1.01-1.11)	0.87** (0.77-0.98)
**Nurses per 1,000 inhabitants[n]**	0.85*** (0.78-0.93)	0.85* (0.73-0.99)	1.65** (1.16-2.34)
**Hospital beds per 1,000 inhabitants[n]**	0.99 (0.98-1.01)	0.99** (0.99-0.99)	0.96 (0.92-1.01)
**Individual`s year of entry into the cohort**	yes	yes	yes
Obs.:	16,180,581	16,180,599	9,570

Notes: *** p-value <0.001; **p-value <0.01; *p-value <0.05. [a] Incidence Rate Ratios. [b] Confidence interval. [c] Aged between 13 and 24. [d] Aged between 25 and 64. [e] Aged 65 or older. [f] Measured by capita expenses proportional to the baseline minimum wage (MW). Level 1 (More wealth): “1 or more”. Level 2: “0.5 to 1”. Level 3: “0.25 to 0.49”. Level 4: “0< to 0.24”. Level 5 (Lower wealth): “Nothing declared”. [g] Water supply: Other – well, spring, and others. [h] Housing Material: No – Coated clay, uncoated clay, wood, and others. [i] Lighting: Non-electric – No meter, lamps, candles, and others. [j] Average rates for the period (2007-2015) by municipality. [l] % of the municipal population with inadequate baseline sanitation. [m] Baseline municipal unemployment rate. [n] Per 1,000 inhabitants of the baseline municipality. All statistical tests used where two-sided and, where appropriate, adjustments were made for multiple comparisons.

Table S21. Estimates of the average effect of the *Programa Bolsa Famlília* (PBF) adjusted Poisson model (with robust standard errors) on AIDS incidence, mortality, and the case-fatality rate, 2007-2015 - with missings category.

Adjusted Model	Outcomes (IRR[a] – CI[b] 95%)		
	Incidence	Mortality	Case-Fatality
**PBF**	0.61*** (0.59-0.63)	0.62*** (0.58-0.65)	0.77*** (0.69-0.85)
**Sex**			
Female	1 (base)	1 (base)	1 (base)
Male	1.29*** (1.25-1.32)	1.41*** (1.34-1.48)	1.28*** (1.15-1.42)
**Age**			
Adolescents and young people[c]	1 (base)	1 (base)	1 (base)
Adults[d]	2.05*** (1.97-2.13)	3.57*** (3.29-3.88)	1.68*** (1.44-1.96)
Older people[e]	0.33*** (0.29-0.37)	0.81* (0.67-0.97)	2.42*** (1.64-3.58)
Missing	3.01 (1.04-1.24)	0.00*** (0.00-0.00)	-
**Race/ethnicity**			
White	1 (base)	1 (base)	1 (base)
Mixed-race	1.23*** (1.18-1.27)	1.23*** (1.15-1.31)	1.02 (0.89-1.16)
Black	1.61*** (1.53-1.69)	1.75*** (1.61-1.90)	0.96 (0.81-1.14)
Indigenous	1.34* (1.00-1.79)	0.97 (0.56-1.70)	0.99 (0.41-2.38)
Missing	1.14* (1.04-1.24)	1.11 (0.95-1.29)	0.95 (0.71-1.27)
**Education**			
More than high school	1 (base)	1 (base)	1 (base)
High school	1.02 (0.90-1.16)	1.50* (1.14-1.97)	2.04* (1.06-3.54)
Elementary school	1.40*** (1.24-1.59)	2.74*** (2.09-3.59)	2.98*** (1.73-5.11)
Attended pre-school	0.90 (0.72-1.13)	1.40 (0.91-2.17)	1.46 (0.59-3.62)
Never attended school	1.28*** (1.13-1.47)	2.88*** (2.17-3.81)	3.93*** (2.24-6.88)
Missing	0.91 (0.80-1.04)	1.46* (1.10-1.94)	2.20** (1.25-3.86)
**Wealth[f]**			
Level 1 (More wealth)	1 (base)	1 (base)	1 (base)
Level 2	0.98 (0.89-1.07)	0.96 (0.82-1.13)	1.29 (0.90-1.86)
Level 3	1.14* (1.04-1.25)	1.22* (1.04-1.42)	1.63* (1.14-2.32)
Level 4	1.55*** (1.42-1.70)	1.59*** (1.35-1.86)	1.36 (0.95-1.95)
Level 5 (Lower wealth)	2.10*** (1.91-2.31)	2.37*** (2.00-2.79)	1.55* (1.07-2.25)
Missing	1.82 (0.69-4.75)	2.71 (0.54-13.57)	-
**AIDS treatment**			
Yes	x	x	1 (base)
No	x	x	2.80*** (2.48-3.16)
**Water supply**			
Public network	1 (base)	1 (base)	1 (base)
Other[g]	1.01 (0.96-1.05)	1.06 (0.98-1.15)	1.03 (0.89-1.20)
Missing	1.60* (1.00-2.55)	2.32* (1.08-4.98)	1.05 (0.72-1.53)
**Housing material (brick)**			
Yes	1 (base)	1 (base)	1 (base)
No[h]	1.24*** (1.19-1.30)	1.35*** (1.25-1.45)	1.21* (1.04-1.39)
Missing	0.54 (0.19-1.49)	0.26 (0.04-1.39)	-
**Lighting**			
Electricity	1 (base)	1 (base)	1 (base)
Non-electric[i]	1.31*** (1.25-1.37)	1.39*** (1.28-1.49)	1.13* (0.97-1.31)
Missing	1.58 (0.88-2.81)	2.44 (0.94-6.26)	-
**Region**			
North	1 (base)	1 (base)	1 (base)
Northeast	1.18*** (1.11-1.25)	1.05 (0.94-1.17)	1.02 (0.83-1.26)
Southeast	1.17*** (1.09-1.24)	1.35*** (1.21-1.50)	1.18 (0.93-1.47)
South	1.47*** (1.37-1.58)	1.41*** (1.24-1.61)	0.96 (0.73-1.27)
Central-west	1.24*** (1.14-1.34)	1.19* (1.03-1.37)	1.24 (0.94-1.64)
Missing	2.22 (0.55-8.93)	0.00*** (0.00-0.00)	
**Area of residence**			
Rural	1 (base)	1 (base)	1 (base)
Urban	1.87*** (1.76-1.99)	2.15*** (1.92-2.40)	1.05 (0.85-1.30)
Missing	4.13*** (3.40-5.00)	4.42*** (3.13-6.23)	0.56 (0.29-1.09)
**Mun. average AIDS incidence rate[j]**	1.03*** (1.02-1.03)	x	x
**Mun. average AIDS mortality rate[j]**	x	1.10*** (1.09-1.10)	x
**Mun. average AIDS case-fatality rate[j]**	x	x	1.01 (0.99-1.02)
**Inadequate sanitation[l]**	0.99*** (0.99-0.99)	0.99*** (0.98-0.99)	0.99 (0.99-1.00)
**Unemployment rate (%)[m]**	1.01*** (1.01-1.02)	1.02*** (1.01-1.03)	1.00 (0.98-1.02)
**Doctors per 1,000 inhabitants[n]**	1.03* (1.00-1.06)	1.06* (1.01-1.11)	0.85*** (0.77-0.93)
**Nurses per 1,000 inhabitants[n]**	0.84*** (0.78-0.91)	0.77* (0.67-0.88)	1.47** (1.11-1.93)
**Hospital beds per 1,000 inhabitants[n]**	0.99 (0.98-1.00)	1.00 (0.98-1.01)	0.99 (0.96-1.02)
**Individual`s year of entry into the cohort**	yes	yes	yes
Obs.:	22,788,743	22,788,743	13,744

Notes: *** p-value <0.001; **p-value <0.01; *p-value <0.05. [a] Incidence Rate Ratios. [b] Confidence interval. [c] Aged between 13 and 24. [d] Aged between 25 and 64. [e] Aged 65 or older. [f] Measured by capita expenses proportional to the baseline minimum wage (MW). Level 1 (More wealth): "1 or more". Level 2: "0.5 to 1". Level 3: "0.25 to 0.49". Level 4: "0< to 0.24". Level 5 (Lower wealth): "Nothing declared". [g] Water supply: Other – well, spring, and others. [h] Housing

Material: No – Coated clay, uncoated clay, wood, and others. [i] Lighting: Non-electric – No meter, lamps, candles, and others. [j] Average rates for the period (2007-2015) by municipality. [l] % of the municipal population with inadequate baseline sanitation. [m] Baseline municipal unemployment rate. [n] Per 1,000 inhabitants of the baseline municipality. All statistical tests used where two-sided and, where appropriate, adjustments were made for multiple comparisons.

5.Triangulation Analyses

We did triangulation analyses using alternative methodologies, including survival models and propensity score matching (PSM). The results estimated using the survival models were weighted by the Inverse Probability of Treatment Weight (IPTW).

Table S22. Estimates of the average effect of the Programa Bolsa Famlília (PBF) Survival Analysis and Propensity Score Matching models on AIDS incidence, mortality, and the case-fatality rate, 2007-2015.

Adjusted Model	Survival Analysis (Hazard Ratio)			Propensity Score Matching Analysis (Odds Ratio)		
	**Incidence**	**Mortality**	**Case-Fatality**	**Incidence**	**Mortality**	**Case-Fatality**
**PBF**	0.65***(0.62-0.67)	0.65***(0.61-0.69)	0.86**(0.77-0.95)	0.68*** (0.65-0.70)	0.68*** (0.64-0.71)	0.46*** (0.35-0.55)
**Sex**					**Logistic regression**	
Female	1 (base)	1 (base)	1 (base)	1 (base)	1 (base)	1 (base)
Male	1.22***(1.18-1.26)	1.36***(1.28-1.43)	1.24***(1.12-1.38)	-0.16***(-0.17:-0.16)	-0.16***(-0.17:-0.16)	-0.64***(-0.72:-0.55)
**Age**						
Adolescents and young people[c]	1 (base)	1 (base)	1 (base)	1 (base)	1 (base)	1 (base)
Adults[d]	2.03***(1.94-2.11)	3.51***(3.21-3.83)	1.82***(1.54-2.16)	-0.26***(-0.26:-0.26)	-0.26***(-0.26:-0.26)	-0.41***(-0.53:-0.29)
Older people[e]	0.33***(0.29-0.38)	0.81* (0.65-0.99)	3.07***(2.07-4.57)	-2.29***(-2.29:-2.28)	-2.29***(-2.30:-2.28)	-2.4***(-2.99:-1.95)
**Race/ethnicity**						
White	1 (base)	1 (base)	1 (base)	1 (base)	1 (base)	1 (base)
Mixed-race	1.23***(1.18-1.28)	1.23***(1.14-1.31)	1.04 (0.91-1.19)	0.07***(0.06-0.07)	0.07***(0.07-0.08)	0.01 (-0.09-0.12)
Black	1.65***(1.56-1.74)	1.77***(1.63-1.93)	1.03 (0.86-1.22)	0.11***(0.10-0.11)	0.11***(0.10-0.11)	0.19** (0.05-0.34)
Indigenous	1.37*(1.01-1.85)	0.90 (0.49-1.62)	0.59 (0.22-1.58)	1.83***(1.82-1.85)	1.85***(1.83-1.86)	0.86* (0.10-1.62)
**Education**						
More than high school	1 (base)	1 (base)	1 (base)	1 (base)	1 (base)	1 (base)
High school	1.02 (0.89-1.16)	1.43* (1.06-1.92)	1.89* (1.05-3.39)	0.81***(0.81-0.83)	0.81***(0.81-0.82)	0.95***(0.58-1.32)
Elementary school	1.38***(1.21-1.57)	2.58***(1.93-3.45)	2.61* (1.46-4.64)	0.96***(0.95-0.97)	0.96***(0.95-0.97)	1.15***(0.79-1.51)
Attended pre-school	0.90 (0.71-1.13)	1.19 (0.74-1.90)	1.02 (0.39-2.64)	0.78***(0.77-0.80)	0.78***(0.77-0.80)	0.83* (0.16-1.49)
Never attended school	1.27**(1.10-1.46)	2.72*** (2.02-3.67)	3.31***(1.82-6.00)	0.83***(0.82-0.84)	0.83***(0.82-0.84)	0.91***(0.52-1.30)
**Wealth[f]**						
Level 1 (More wealth)	1 (base)	1 (base)	1 (base)	1 (base)	1 (base)	1 (base)
Level 2	0.98(0.89-1.08)	0.91 (0.76-1.07)	1.21 (0.81-1.79)	0.82***(0.81-0.83)	0.82***(0.81-0.83)	0.59***(0.30-0.88)
Level 3	1.16**(1.05-1.27)	1.21* (1.02-1.43)	1.51* (1.03-2.22)	1.73***(1.72-1.74)	1.73***(1.72-1.74)	1.60***(1.32-1.89)
Level 4	1.56***(1.42-1.72)	1.54***(1.30-1.83)	1.25 (0.85-1.83)	2.35***(2.35-2.36)	2.35***(2.35-2.36)	2.15***(1.88-2.43)
Level 5 (Lower wealth)	2.11***(1.91-2.34)	2.32***(1.93-2.77)	1.46 (0.98-2.17)	2.21***(2.20-2.22)	2.21***(2.20-2.21)	2.31***(2.02-2.60)
**AIDS treatment**						
Yes	x	x	1 (base)	x	x	1 (base)
No	x	x	2.44***(2.16-2.75)	x	x	0.12* (0.01-0.23)
**Water supply**						
Public network	1 (base)	1 (base)	1 (base)	1 (base)	1 (base)	1 (base)
Other[g]	1.01(0.95-1.05)	1.07 (0.98-1.16)	1.02 (0.87-1.19)	0.13***(0.13-0.14)	0.13***(0.13-0.14)	0.17* (0.03-0.30)
**Housing material (brick)**						
Yes	1 (base)	1 (base)	1 (base)	1 (base)	1 (base)	1 (base)
No [h]	1.24***(1.18-1.29)	1.33*** (1.24-1.44)	1.18* (1.02-1.36)	0.08***(0.07-0.08)	0.07***(0.07-0.08)	-0.02 (-0.15-0.10)
**Lighting**						
Electricity	1 (base)	1 (base)	1 (base)	1 (base)	1 (base)	1 (base)
Non-electric [i]	1.30***(1.23-1.36)	1.35*** (1.25-1.46)	1.19* (1.03-1.38)	0.22***(0.21-0.22)	0.22***(0.22-0.23)	0.45*** (0.32-0.58)
**Region**						
North	1 (base)	1 (base)	1 (base)	1 (base)	1 (base)	1 (base)
Northeast	1.18***(1.11-1.26)	1.03 (0.92-1.16)	1.05 (0.83-1.32)	-0.08***(-0.09:--0.08)	-0.12***(-0.12:-0.11)	0.2 (-0.16-0.21)
Southeast	1.19***(1.10-1.27)	1.34*** (1.19-1.51)	1.11 (0.87-1.43)	0.29***(0.28-0.29)	0.20*** (0.19-0.20)	0.26* (0.06-0.47)

South	1.51***(1.40-1.64)	1.43*** (1.24-1.65)	0.91 (0.69-1.19)	-0.38***(-0.39:-0.38)	-0.42***(-0.43:0.41)	-0.16 (-0.38-0.05)
Central-west	1.21***(1.11-1.32)	1.19* (1.02-1.40)	1.16 (0.86-1.57)	-0.15***(-0.16:-0.15)	-0.21***(-0.21:-0.20)	-0.25 (-0.50-0.00)
**Area of residence**						
Rural	1 (base)	1 (base)	1 (base)	1 (base)	1 (base)	1 (base)
Urban	1.91***(1.78-2.04)	2.21***(1.19-2.48)	1.08 (0.83-1.39)	0.04***(0.04-0.05)	0.05***(0.05-0.06)	-0.21 (-0.43-0.00)
**Mun. average AIDS incidence rate**[j]	1.03***(1.02-1.03)	x	x	0.01***(0.01-0.01)	x	x
**Mun. average AIDS mortality rate**[j]	x	1.10*** (1.09-1.10)	x	x	0.04*** (0.04-0.04)	x
**Mun. average AIDS case-fatality rate**[j]	x	x	1.01*** (1.00-1.02)	x	x	0.00* (0.00-0.01)
**Inadequate sanitation**[l]	0.99*** (0.99-0.99)	0.99*** (0.98-0.99)	1.00 (0.99-1.01)	-0.00***(-0.0:-0.0)	-0.00***(-0.00:-0.00)	-0.00 (-0.01-0.00)
**Unemployment rate (%)**[m]	1.01*** (1.01-1.02)	1.02*** (1.01-1.03)	0.98 (0.96-1.01)	0.01***(0.01-0.1)	0.01***(0.01-0.1)	0.02* (0.00-0.04)
**Doctors per 1,000 inhabitants**[n]	1.02 (0.99-1.05)	1.05* (1.00-1.10)	0.86* (0.78-0.95)	0.11***(0.11-0.11)	0.13***(0.13-0.14)	-0.06 (-0.14:-0.12)
**Nurses per 1,000 inhabitants**[n]	0.85*** (0.78-0.92)	0.82* (0.70-0.95)	1.61** (1.21-2.14)	0.03***(0.02-0.03)	0.03***(0.03-0.04)	0.39** (0.15-0.62)
**Hospital beds per 1,000 inhabitants**[n]	0.99 (0.98-1.01)	0.99 (0.98-1.01)	0.98 (0.94-1.02)	-0.02***(-0.02:-0.03)	-0.03***(-0.03:-0.03)	-0.00 (-0.04-0.03)
**Individual`s year of entry into the cohort**	yes	yes	yes	yes	yes	yes
Obs.:	19,577,629	19,577,649	9,965	19,577,880	19,577,880	10,423

Notes: *** p-value <0.001; **p-value <0.01; *p-value <0.05. [a] Incidence Rate Ratios. [b] Confidence interval. [c] Aged between 13 and 24. [d] Aged between 25 and 64. [e] Aged 65 or older. [f] Measured by capita expenses proportional to the baseline minimum wage (MW). Level 1 (More wealth): "1 or more". Level 2: "0.5 to 1". Level 3: "0.25 to 0.49". Level 4: "0< to 0.24". Level 5 (Lower wealth): "Nothing declared". [g] Water supply: Other – well, spring, and others. [h] Housing Material: No – Coated clay, uncoated clay, wood, and others. [i] Lighting: Non-electric – No meter, lamps, candles, and others. [j] Average rates for the period (2007-2015) by municipality. [l] % of the municipal population with inadequate baseline sanitation. [m] Baseline municipal unemployment rate. [n] Per 1,000 inhabitants of the baseline municipality. All statistical tests used where two-sided and, where appropriate, adjustments were made for multiple comparisons.

6. IPTW Poisson regression unadjusted

To verify the importance of adjusting for all covariates in Poisson multivariate regression with IPTW, we performed the regressions of Poisson bivariate models with IPTW only between AIDS outcomes and PBF exposure, that is, unadjusted models (Table S20).

Table S23. Estimates of the average effect of the *Programa Bolsa Famlília* (PBF), unadjusted Poisson model (with robust standard error), on AIDS incidence, mortality, and case-fatality rate, 2007-2015.

Unadjusted Model	Outcomes (IRR[a] – IC[b] 95%)		
	**Incidence**	**Mortality**	**Case-Fatality**
**PBF**	0.66*** (0.64-0.69)	0.68*** (0.64-0.72)	0.74*** (0.66-0.83)
Obs.:	19,577,629	19,577,649	9,965

Notes: *** p-value <0,001; **p-valor <0,01; *p-valor <0,05. [a] Incidence Rate Ratios.[b] Confidence Interval. All statistical tests used where two-sided and, where appropriate, adjustments were made for multiple comparisons.

7. Complementary analyses

Table S24. Distribution of *Programa Bolsa Famlília* (PBF) beneficiaries and non-beneficiaries by marital status, 2007-2015.

Marital status[a]	*Programa Bolsa Familia (PBF)*		Total
	0	1	
**Married**	1,511,423 (28.77%)	769,797 (22.76%)	2,281,220 (26.42%)
**Divorced/Widowed**	395,426 (7.53%)	187,049 (5.53%)	528,475 (6.75%)
**Single**	3,345,960 (63.70%)	2,425,517 (71.71%)	5,771,477 (66.84%)
**Total**	5,252,809 (100.00%)	3,382,363 (100.00%)	8,635,172 (100.00%)

Note: [a] Individuals with declared information in the Cohort.

Table S25. Estimates of the average effect of the *Programa Bolsa Famlília* (PBF) adjusted Poisson model (with robust standard errors) on AIDS incidence rate, 2007-2015. With marital status (with and without missing values).

Adjusted Model	Incidence (IRR[a] – CI[b] 95%)	Incidence (IRR[a] – CI[b] 95%)
**PBF**	0.49*** (0.47-0.51)	0.58*** (0.56-0.60)
**Sex**		
Female	1 (base)	1 (base)
Male	1.03 (0.99-1.07)	1.28*** (1.23-1.32)
**Age**		
Adolescents and young people[c]	1 (base)	1 (base)
Adults[d]	2.43*** (2.30-2.56)	2.36*** (2.26-2.47)
Older people[e]	0.32*** (0.26-0.39)	0.40*** (0.35-0.46)
**Race/ethnicity**		
White	1 (base)	1 (base)
Mixed-race	1.18*** (1.12-1.24)	1.22*** (1.16-1.26)
Black	1.56*** (1.46-1.67)	1.61*** (1.52-1.69)
Indigenous	1.21 (0.83-1.77)	1.33 (0.98-1.80)
**Education**		
More than high school	1 (base)	1 (base)
High school	1.28* (1.04-1.58)	1.04 (0.91-1.18)
Elementary school	1.67*** (1.37-2.05)	1.43*** (1.25-1.62)
Attended pre-school	1.03 (0.75-1.42)	0.93 (0.74-1.17)
Never attended school	1.45*** (1.18-1.79)	1.29*** (1.12-1.47)
**Wealth[f]**		
Level 1 (More wealth)	1 (base)	1 (base)
Level 2	1.01 (0.37-2.73)	0.99 (0.90-1.09)
Level 3	1.18 (0.45-3.09)	1.19*** (1.08-1.31)
Level 4	1.36 (0.53-3.52)	1.67*** (1.52-1.85)
Level 5 (Lower wealth)	1.88 (0.73-4.85)	2.15*** (1.94-2.38)
**Marital status**		
Married	1 (base)	1 (base)
Divorced/Widowed	2.86*** (2.09-2.50)	2.45*** (2.24-2.67)
Single	2.90*** (2.72-3.08)	2.91*** (2.74-3.09)
Missing	x	2.53*** (2.31-2.77)
**Water supply**		
Public network	1 (base)	1 (base)
Other[g]	1.00 (0.95-1.06)	1.01 (0.96-1.06)
**Housing material (brick)**		
Yes	1 (base)	1 (base)
No[h]	1.24*** (1.17-1.32)	1.21*** (1.15-1.26)
**Lighting**		
Electricity	1 (base)	1 (base)
Non-electric[i]	1.33*** (1.25-1.41)	1.27*** (1.21-1.33)
**Region**		
North	1 (base)	1 (base)
Northeast	1.28*** (1.18-1.39)	1.19*** (1.11-1.26)
Southeast	1.46*** (1.33-1.59)	1.23*** (1.15-1.32)
South	1.88*** (1.69-2.08)	1.59*** (1.47-1.72)
Central-west	1.41*** (1.26-1.58)	1.25*** (1.14-1.36)
**Area of residence**		
Rural	1 (base)	1 (base)
Urban	1.86*** (1.77-2.01)	1.82*** (1.70-1.95)
**Mun. average AIDS incidence rate[j]**	1.03*** (1.02-1.03)	1.03*** (1.02-1.03)
**Inadequate sanitation[l]**	0.99*** (0.99-0.99)	0.99*** (0.99-0.99)
**Unemployment rate (%)[m]**	1.01*** (1.01-1.02)	1.01*** (1.01-1.02)
**Doctors per 1,000 inhabitants[n]**	1.00 (0.97-1.05)	1.02 (0.99-1.05)
**Nurses per 1,000 inhabitants[n]**	0.85* (0.75-0.96)	0.84*** (0.77-0.92)
**Hospital beds per 1,000 inhabitants[n]**	1.00 (0.99-1.01)	0.99 (0.98-1.01)
**Individual`s year of entry into the cohort**	yes	yes
Obs.:	7,227,923	19,577,629

Notes: *** p-value <0.001; **p-value <0.01; *p-value <0.05. [a] Incidence Rate Ratios. [b] Confidence interval. [c] Aged between 13 and 24. [d] Aged between 25 and 64. [e] Aged 65 or older. [f] Measured by capita expenses proportional to the baseline minimum wage (MW). Level 1 (More wealth): "1 or more". Level 2: "0.5 to 1". Level 3: "0.25 to 0.49". Level 4: "0< to 0.24". Level 5 (Lower wealth): "Nothing declared". [g] Water supply: Other – well, spring, and others. [h] Housing Material: No – Coated clay, uncoated clay, wood, and others. [i] Lighting: Non-electric – No meter, lamps, candles, and others. [j] Average rates for the period (2007-2015) by municipality. [l] % of the municipal population with inadequate baseline sanitation. [m] Baseline municipal unemployment rate. [n] Per 1,000 inhabitants of the baseline municipality. All statistical tests used where two-sided and, where appropriate, adjustments were made for multiple comparisons.

Table S26. Distribution of Programa Bolsa Famlília (PBF) beneficiaries and non-beneficiaries by AIDS exposure category, 2007-2015.

**AIDS exposure category**[a]	***Programa Bolsa Familia (PBF)***		**Total**
	0	1	
Perinatal or accident	2<br>(0.01%)	2<br>(0.02%)	4<br>(0.01%)
Hemophiliac/transfusion	3<br>(0.02%)	0<br>(0.00%)	3<br>(0.01%)
Heterosexual	11,902<br>(74.78%)	8,977<br>(81.46%)	20,879<br>(77.51%)
Homosexual	2,767<br>(17.39%)	1,275<br>(11.57%)	4,042<br>(15.01%)
Bisexual	748<br>(4.70%)	411<br>(3.73%)	1,159<br>(4.30%)
Uso of drugs	494<br>(3.10%)	355<br>(3.22%)	849<br>(3.15%)
**Total**	15,916<br>(100.00%)	11,020<br>(100.00%)	26,936<br>(100.00%)

Note: [a] Individuals with declared information in the Cohort.

Table S27. Estimates of the average effect of the *Programa Bolsa Famlília* (PBF) adjusted Poisson model (with robust standard errors) on AIDS case-fatality rate, 2007-2015 (With marital status and AIDS exposure category).

Adjusted Model	Outcomes (IRR[a] – CI[b] 95%)		
	Case-Fatality	Case-Fatality	Case-Fatality
**PBF**	0.75*** (0.66-0.85)	0.78*** (0.68-0.88)	0.77*** (0.68-0.88)
**Sex**			
Female	1 (base)	1 (base)	1 (base)
Male	1.27*** (1.13-1.44)	1.22*** (1.06-1.39)	1.22*** (1.06-1.40)
**Age**			
Adolescents and young people[c]	1 (base)	1 (base)	1 (base)
Adults[d]	1.72*** (1.42-2.07)	1.59*** (1.32-1.93)	1.61*** (1.33-1.95)
Older people[e]	2.89*** (1.81-4.62)	2.65*** (1.66-4.25)	2.71*** (1.69-4.32)
**Race/ethnicity**			
White	1 (base)	1 (base)	1 (base)
Mixed-race	1.07 (0.92-1.25)	1.04 (0.89-1.22)	1.04 (0.89-1.21)
Black	1.00 (0.83-1.22)	0.99 (0.81-1.21)	0.98 (0.81-1.20)
Indigenous	0.57 (0.20-1.59)	0.58 (0.21-1.64)	0.57 (0.20-1.62)
**Education**			
More than high school	1 (base)	1 (base)	1 (base)
High school	1.96* (1.07-3.59)	1.78 (0.97-3.29)	1.79 (0.97-3.30)
Elementary school	2.78** (1.54-5.05)	2.39** (1.30-4.37)	2.39** (1.31-4.38)
Attended pre-school	1.01 (0.36-2.83)	0.81 (0.27-2.35)	0.81 (0.27-2.39)
Never attended school	3.62*** (1.95-6.74)	3.09*** (1.64-5.91)	3.08*** (1.64-5.80)
**Wealth[f]**			
Level 1 (More wealth)	1 (base)	1 (base)	1 (base)
Level 2	1.23 (0.81-1.88)	1.19 (0.77-1.84)	1.20 (0.77-1.85)
Level 3	1.63* (1.07-2.45)	1.61* (1.06-2.45)	1.62* (1.06-2.48)
Level 4	1.31 (0.87-1.99)	1.29 (0.85-1.97)	1.29 (0.85-1.98)
Level 5 (Lower wealth)	1.56* (1.01-2.40)	1.54* (0.99-2.40)	1.53* (0.98-2.38)
**Marital status**			
Married	1 (base)	x	1 (base)
Divorced/Widowed	1.12 (0.80-1.56)	x	1.12 (0.80-1.58)
Single	1.18 (0.93-1.48)	x	1.17 (0.93-1.48)
Missing	1.11 (0.78-1.57)	x	1.11 (0.78-1.57)
**AIDS exposure category**			
Perinatal ou acidente	x	1 (base)	1 (base)
Hemophiliac/transfusion	x	0.73	0.69
Heterosexual	x	173610.4	169849.8
Homosexual	x	116697.1	113454.7
Bisexual	x	124507.6	121277.7
Use of drugs	x	332214.9	324083.6
Missing	x	355381.5	346673.8
**AIDS treatment**			
Yes	1 (base)	1 (base)	1 (base)
No	2.68*** (2.32-3.08)	2.31*** (1.98-2.68)	2.31*** (1.98-2.68)
**Water supply**			
Public network	1 (base)	1 (base)	1 (base)
Other[g]	1.03 (0.87-1.23)	1.01 (0.84-1.21)	1.01 (0.84-1.21)
**Housing material (brick)**			
Yes	1 (base)	1 (base)	1 (base)
No [h]	1.18* (1.00-1.39)	1.19* (1.00-1.40)	1.19* (1.00-1.40)
**Lighting**			
Electricity	1 (base)	1 (base)	1 (base)
Non-electric [i]	1.21* (1.02-1.43)	1.23* (1.03-1.46)	1.22* (1.03-1.45)
**Region**			
North	1 (base)	1 (base)	1 (base)
Northeast	1.06 (0.81-1.37)	1.02 (0.78-1.33)	1.02 (0.78-1.33)
Southeast	1.10 (0.83-1.46)	1.02 (0.77-1.36)	1.02 (0.77-1.37)
South	0.96 (0.71-1.31)	0.88 (0.64-1.20)	0.88 (0.65-1.21)
Central-west	1.21 (0.86-1.70)	1.16 (0.82-1.64)	1.17 (0.83-1.66)
**Area of residence**			
Rural	1 (base)	1 (base)	1 (base)
Urban	1.06 (0.79-1.42)	1.07 (0.79-1.44)	1.07 (0.79-1.43)
**Mun. average AIDS case-fatality rate[j]**	1.01*** (1.00-1.02)	1.02*** (1.01-1.02)	1.02*** (1.01-1.02)
**Inadequate sanitation [l]**	1.00 (0.99-1.01)	1.00 (0.99-1.01)	1.00 (0.99-1.01)
**Unemployment rate (%)[m]**	0.98 (0.96-1.01)	0.98 (0.96-1.01)	0.98 (0.96-1.01)
**Doctors per 1,000 inhabitants[n]**	0.85** (0.76-0.95)	0.86** (0.76-0.96)	0.86** (0.77-0.96)
**Nurses per 1,000 inhabitants[n]**	1.69** (1.23-2.34)	1.60** (1.15-2.22)	1.58** (1.14-2.19)
**Hospital beds per 1,000 inhabitants[n]**	0.97 (0.92-1.01)	0.97 (0.93-1.02)	0.97 (0.93-1.02)
**Individual`s year of entry into the cohort**	yes	yes	yes
Obs.:	9,965	9,965	9,965

Notes: *** p-value <0.001; **p-value <0.01; *p-value <0.05. [a] Incidence Rate Ratios. [b] Confidence interval. [c] Aged between 13 and 24. [d] Aged between 25 and 64. [e] Aged 65 or older. [f] Measured by capita

expenses proportional to the baseline minimum wage (MW). Level 1 (More wealth): “1 or more”. Level 2: “0.5 to 1”. Level 3: “0.25 to 0.49”. Level 4: “0< to 0.24”. Level 5 (Lower wealth): “Nothing declared”. [g] Water supply: Other – well, spring, and others. [h] Housing Material: No – Coated clay, uncoated clay, wood, and others. [i] Lighting: Non-electric – No meter, lamps, candles, and others. [j] Average rates for the period (2007-2015) by municipality. [l] % of the municipal population with inadequate baseline sanitation. [m] Baseline municipal unemployment rate. [n] Per 1,000 inhabitants of the baseline municipality. All statistical tests used where two-sided and, where appropriate, adjustments were made for multiple comparisons.

Table S28. Estimates of the average effect of the *Programa Bolsa Famlília* (PBF) adjusted Poisson model (with robust standard errors) on HIV notification rate, after 2013.

Adjusted Model	Incidence (IRR[a] – CI[b] 95%)
**PBF**	0.64*** (0.62-0.66)
**Sex**	
Female	1 (base)
Male	0.88*** (0.85-0.91)
**Age**	
Adolescents and young people[c]	1 (base)
Adults[d]	0.99 (0.96-1.02)
Older people[e]	0.81** (0.70-0.93)
**Race/ethnicity**	
White	1 (base)
Mixed-race	1.02 (0.98-1.05)
Black	1.02 (0.97-1.07)
Indigenous	0.95 (0.82-1.10)
**Education**	
More than high school	1 (base)
High school	1.03 (0.95-1.12)
Elementary school	1.08 (0.99-1.17)
Attended pre-school	1.04 (0.87-1.24)
Never attended school	1.04 (0.94-1.15)
**Wealth[f]**	
Level 1 (More wealth)	1 (base)
Level 2	0.99 (0.94-1.05)
Level 3	1.07 (1.01-1.14)
Level 4	1.15*** (1.09-1.22)
Level 5 (Lower wealth)	1.19*** (1.11-1.28)
**Water supply**	
Public network	1 (base)
Other[g]	1.02 (0.87-1.06)
**Housing material (brick)**	
Yes	1 (base)
No [h]	1.01 (0.96-1.04)
**Lighting**	
Electricity	1 (base)
Non-electric [i]	1.03 (0.98-1.08)
**Region**	
North	1 (base)
Northeast	1.00 (0.95-1.06)
Southeast	1.03 (0.96-1.08)
South	1.03 (0.96-1.09)
Central-west	1.04 (0.96-1.11)
**Area of residence**	
Rural	1 (base)
Urban	0.97 (0.92-1.03)
**Inadequate sanitation [l]**	1.00*** (1.00-1.00)
**Unemployment rate (%)[m]**	1.00 (0.99-1.01)
**Doctors per 1,000 inhabitants[n]**	0.99 (0.96-1.01)
**Nurses per 1,000 inhabitants[n]**	1.06 (0.99-1.14)
**Hospital beds per 1,000 inhabitants[n]**	0.99 (0.98-1.01)
**Individual`s year of entry into the cohort**	yes
Obs.:	5,983

Notes: *** p-value <0.001; **p-value <0.01; *p-value <0.05. [a] Incidence Rate Ratios. [b] Confidence interval. [c] Aged between 13 and 24. [d] Aged between 25 and 64. [e] Aged 65 or older. [f] Measured by capita expenses proportional to the baseline minimum wage (MW). Level 1 (More wealth): "1 or more". Level 2: "0.5 to 1". Level 3: "0.25 to 0.49". Level 4: "0< to 0.24". Level 5 (Lower wealth): "Nothing declared". [g] Water supply: Other – well, spring, and others. [h] Housing Material: No – Coated clay, uncoated clay, wood, and others. [i] Lighting: Non-electric – No meter, lamps, candles, and others. [j] Average rates for the period (2007-2015) by municipality. [l] % of the municipal population with inadequate baseline sanitation. [m] Baseline municipal unemployment rate. [n] Per 1,000 inhabitants of the baseline municipality. All statistical tests used where two-sided and, where appropriate, adjustments were made for multiple comparisons.

Table S29. Estimates of the average effect of the *Programa Bolsa Famlília* (PBF) adjusted Poisson model (with robust standard errors) on AIDS incidence rate - as a new follow-up time for the group exposed to PBF.

Adjusted Model	Incidence (IRR[a] – CI[b] 95%)
**PBF**	0.71*** (0.68-0.73)
**Sex**	
Female	1 (base)
Male	1.23*** (1.19-1.27)
**Age**	
Adolescents and young people[c]	1 (base)
Adults[d]	2.02*** (1.93-2.10)
Older people[e]	0.33*** (0.29-0.38)
**Race/ethnicity**	
White	1 (base)
Mixed-race	1.23*** (1.18-1.28)
Black	1.64*** (1.56-1.73)
Indigenous	1.33 (0.98-1.79)
**Education**	
More than high school	1 (base)
High school	1.01 (0.88-1.15)
Elementary school	1.36*** (1.19-1.54)
Attended pre-school	0.88 (0.69-1.11)
Never attended school	1.24** (1.08-1.43)
**Wealth[f]**	
Level 1 (More wealth)	1 (base)
Level 2	0.97 (0.88-1.07)
Level 3	1.12* (1.02-1.24)
Level 4	1.50*** (1.36-1.65)
Level 5 (Lower wealth)	2.02*** (1.83-2.25)
**Water supply**	
Public network	1 (base)
Other[g]	1.01 (0.96-1.06)
**Housing material (brick)**	
Yes	1 (base)
No [h]	1.24*** (1.18-1.29)
**Lighting**	
Electricity	1 (base)
Non-electric [i]	1.30*** (1.24-1.36)
**Region**	
North	1 (base)
Northeast	1.18*** (1.11-1.26)
Southeast	1.21*** (1.13-1.29)
South	1.55*** (1.44-1.68)
Central-west	1.24*** (1.14-1.35)
**Area of residence**	
Rural	1 (base)
Urban	1.91*** (1.78-2.04)
**Mun. average AIDS incidence rate[j]**	1,03*** (1.02-1.03)
**Inadequate sanitation [l]**	0.99*** (0.99-0.99)
**Unemployment rate (%)[m]**	1.01*** (1.01-1.02)
**Doctors per 1,000 inhabitants[n]**	1.02 (0.99-1.06)
**Nurses per 1,000 inhabitants[n]**	0.85*** (0.78-0.92)
**Hospital beds per 1,000 inhabitants[n]**	0.99 (0.98-1.01)
**Individual`s year of entry into the cohort**	yes
Obs.:	19,577,629

Notes: *** p-value <0.001; **p-value <0.01; *p-value <0.05. [a] Incidence Rate Ratios. [b] Confidence interval. [c] Aged between 13 and 24. [d] Aged between 25 and 64. [e] Aged 65 or older. [f] Measured by capita expenses proportional to the baseline minimum wage (MW). Level 1 (More wealth): "1 or more". Level 2: "0.5 to 1". Level 3: "0.25 to 0.49". Level 4: "0< to 0.24". Level 5 (Lower wealth): "Nothing declared". [g] Water supply: Other – well, spring, and others. [h] Housing Material: No – Coated clay, uncoated clay, wood, and others. [i] Lighting: Non-electric – No meter, lamps, candles, and others. [j] Average rates for the period (2007-2015) by municipality. [l] % of the municipal population with inadequate baseline sanitation. [m] Baseline municipal unemployment rate. [n] Per 1,000 inhabitants of the baseline municipality. All statistical tests used where two-sided and, where appropriate, adjustments were made for multiple comparisons.

Table S30. Estimates of the average effect of the *Programa Bolsa Famlília* (PBF) adjusted Poisson model (with robust standard errors) on AIDS Case-fatality rate, 2007-2015 - without ART adherence.

Adjusted Model	Case-fatality (IRR[a] – CI[b] 95%)
**PBF**	0.76*** (0.67-0.86)
**Sex**	
Female	1 (base)
Male	1.27*** (1.14-1.43)
**Age**	
Adolescents and young people[c]	1 (base)
Adults[d]	1.64*** (1.37-1.96)
Older people[e]	2.88*** (1.82-4.56)
**Race/ethnicity**	
White	1 (base)
Mixed-race	1.09 (0.94-1.26)
Black	1.06 (0.88-1.28)
Indigenous	0.58 (0.21-1.66)
**Education**	
More than high school	1 (base)
High school	2.01* (1.08-3.74)
Elementary school	2.83** (1.53-5.20)
Attended pre-school	1.17 (0.44-3.12)
Never attended school	3.80*** (2.02-7.17)
**Wealth[f]**	
Level 1 (More wealth)	1 (base)
Level 2	1.22 (0.81-1.86)
Level 3	1.64* (1.09-2.46)
Level 4	1.38 (0.92-2.07)
Level 5 (Lower wealth)	1.63** (1.07-2.47)
**Water supply**	
Public network	1 (base)
Other[g]	1.03 (0.87-1.22)
**Housing material (brick)**	
Yes	1 (base)
No [h]	1.19** (1.01-1.40)
**Lighting**	
Electricity	1 (base)
Non-electric [i]	1.27** (1.04-1.44)
**Region**	
North	1 (base)
Northeast	1.12 (0.87-1.44)
Southeast	1.29 (0.99-1.69)
South	0.92 (0.68-1.24)
Central-west	1.21 (0.87-1.69)
**Area of residence**	
Rural	1 (base)
Urban	1.06 (0.70-1.41)
**Mun. average AIDS case-fatality rate[j]**	1,01*** (1.00-1.02)
**Inadequate sanitation [l]**	1.00 (0.99-1.01)
**Unemployment rate (%)[m]**	0.99 (0.97-1.02)
**Doctors per 1,000 inhabitants[n]**	0.88* (0.80-0.98)
**Nurses per 1,000 inhabitants[n]**	1.55** (1.14-2.10)
**Hospital beds per 1,000 inhabitants[n]**	0.98 (0.94-1.02)
**Individual`s year of entry into the cohort**	yes
Obs.:	9,965

Notes: *** p-value <0.001; **p-value <0.01; *p-value <0.05. [a] Incidence Rate Ratios. [b] Confidence interval. [c] Aged between 13 and 24. [d] Aged between 25 and 64. [e] Aged 65 or older. [f] Measured by capita expenses proportional to the baseline minimum wage (MW). Level 1 (More wealth): “1 or more”. Level 2: “0.5 to 1”. Level 3: “0.25 to 0.49”. Level 4: “0< to 0.24”. Level 5 (Lower wealth): “Nothing declared”. [g] Water supply: Other – well, spring, and others. [h] Housing Material: No – Coated clay, uncoated clay, wood, and others. [i] Lighting: Non-electric – No meter, lamps, candles, and others. [j] Average rates for the period (2007-2015) by municipality. [l] % of the municipal population with inadequate baseline sanitation. [m] Baseline municipal unemployment rate. [n] Per 1,000 inhabitants of the baseline municipality. All statistical tests used where two-sided and, where appropriate, adjustments were made for multiple comparisons.

Table S31. Covariate balancing test before and after weighting by IPTW.

Variables	Before-IPTW			After-IPTW		
	BF	N-BF	Difference	BF	N-BF	Difference
**Sex**	0.3749	0.4410	-0.07*	0.3802	0.3831	0.00
**Age**	0.6855	0.8874	-0.20*	0.6764	0.6779	0.00
**Race/ethnicity**	0.8062	0.6932	0.11*	0.8052	0.8033	0.00
**Education**	1.8585	1.8705	-0.01	1.8679	1.8589	0.01
**Wealth**	2.8759	2.1751	0.70*	2.8461	2.8358	0.01
**Water supply**	0.2515	0.2039	0.05	0.2589	0.2479	0.01
**Housing material (brick)**	0.1823	0.1585	0.02	0.1897	0.1763	0.01
**Lighting**	0.1508	0.0804	0.07	0.1559	0.1502	0.01
**Region**	2.6770	2.8432	-0.17*	2.6801	2.6770	0.00
**Area of residence**	0.8211	0.8261	0.00	0.8152	0.8294	-0.01
**Mun. average AIDS incidence rate**	21.661	19.139	0.25*	21.478	21.875	-0.40
**Mun. average AIDS mortality rate**	6.4521	5.6316	0.82*	6.3985	6.5145	-0.12
**Mun. average AIDS case-fatality rate**	30.861	30.885	-0.02	30.876	30.840	0.04
**Inadequate sanitation**	10.892	10.013	0.88*	11.006	10.681	0.33
**Unemployment rate (%)**	8.4605	7.3827	1.08*	8.3862	8.4133	-0.03
**Doctors per 1,000 inhabitants**	1.4682	1.3458	0.12*	1.4624	1.4966	-0.03
**Nurses per 1,000 inhabitants**	0.6741	0.6927	-0.02	0.6739	0.6856	-0.01
**Hospital beds per 1,000 inhabitants**	2.3326	2.3213	0.01	2.3250	2.3332	-0.01

Notes: *p-value <0.05. Mean difference test between BF and N-BF (null hypothesis: mean difference equal to zero). All statistical tests used where two-sided and, where appropriate, adjustments were made for multiple comparisons.

Table S32. Estimates of the average effect of the *Programa Bolsa Famlília* (PBF) adjusted Poisson model (with robust standard errors) on AIDS incidence rate, 2007-2015 - Interaction between PBF (BF=1) and Sex (male=1).

**Adjusted Model**	**Incidence (IRR[a] – CI[b] 95%)**
**PBF**	0.61*** (0.58-0.64)
**Sex**	
Female	1 (base)
Male	1.23*** (1.18-1.28)
**PBF*Sex**	0.94 (0.88-1.01)
**Age**	
Adolescents and young people[c]	1 (base)
Adults[d]	2.02*** (1.93-2.10)
Older people[e]	0.32*** (0.28-0.37)
**Race/ethnicity**	
White	1 (base)
Mixed-race	1.24*** (1.19-1.29)
Black	1.65*** (1.57-1.75)
Indigenous	1.36 (0.99-1.83)
**Education**	
More than high school	1 (base)
High school	1.03 (0.90-1.18)
Elementary school	1.41*** (1.24-1.61)
Attended pre-school	0.91 (0.72-1.15)
Never attended school	1.28*** (1.12-1.47)
**Wealth[f]**	
Level 1 (More wealth)	1 (base)
Level 2	0.99 (0.89-1.09)
Level 3	1.16* (1.05-1.28)
Level 4	1.57*** (1.42-1.73)
Level 5 (Lower wealth)	2.13*** (1.92-2.36)
**Water supply**	
Public network	1 (base)
Other[g]	1.01 (0.96-1.06)
**Housing material (brick)**	
Yes	1 (base)
No [h]	1.25*** (1.19-1.30)
**Lighting**	
Electricity	1 (base)
Non-electric [i]	1.31*** (1.25-1.38)
**Region**	
North	1 (base)
Northeast	1.19*** (1.11-1.27)
Southeast	1.19*** (1.11-1.28)
South	1.52*** (1.40-1.65)
Central-west	1.23*** (1.12-1.34)
**Area of residence**	
Rural	1 (base)
Urban	1.89*** (1.77-2.03)
**Mun. average AIDS incidence rate[j]**	1,03*** (1.02-1.03)
**Inadequate sanitation [l]**	0.99*** (0.99-0.99)
**Unemployment rate (%)[m]**	1.01*** (1.01-1.02)
**Doctors per 1,000 inhabitants[n]**	1.03 (0.99-1.06)
**Nurses per 1,000 inhabitants[n]**	0.85*** (0.78-0.93)
**Hospital beds per 1,000 inhabitants[n]**	0.99 (0.98-1.01)
**Individual`s year of entry into the cohort**	yes
Obs.:	19,577,629

Notes: *** p-value <0.001; **p-value <0.01; *p-value <0.05. [a] Incidence Rate Ratios. [b] Confidence interval. [c] Aged between 13 and 24. [d] Aged between 25 and 64. [e] Aged 65 or older. [f] Measured by capita expenses proportional to the baseline minimum wage (MW). Level 1 (More wealth): "1 or more". Level 2: "0.5 to 1". Level 3: "0.25 to 0.49". Level 4: "0< to 0.24". Level 5 (Lower wealth): "Nothing declared". [g] Water supply: Other – well, spring, and others. [h] Housing Material: No – Coated clay, uncoated clay, wood, and others. [i] Lighting: Non-electric – No meter, lamps, candles, and others. [j] Average rates for the period (2007-2015) by municipality. [l] % of the municipal population with inadequate baseline sanitation. [m] Baseline municipal unemployment rate. [n] Per 1,000 inhabitants of the baseline municipality. All statistical tests used where two-sided and, where appropriate, adjustments were made for multiple comparisons.

8. IPTW Poisson regression stratified models

Tables S31-S35 present all models of Poisson regressions with IPTW, unadjusted and adjusted, for all socioeconomic and demographic variables, for the association between AIDS incidence, mortality and fatality rates and the *Programa Bolsa Família* (PBF) by wealth subpopulations, sex and age (Brazil, 2007-2015).

Table S33. Estimates of the average effect of the *Programa Bolsa Famlília* (PBF) adjusted Poisson model (with robust standard errors) on AIDS incidence rate in Brazil, 2007-2015.

Models	Incidence (IRR[a] – CI[b] 95%)				
	Total	Wealth[f]			
		Quartile 1[o]	Quartile 2[p]	Quartile 3[q]	Quartile 4[r]
**Unadjusted Model**					
**PBF**	0.66*** (0.64-0.68)	0.45*** (0.42-0.46)	0.67*** (0.63-0.72)	0.86*** (0.79-0.93)	1.02 (0.89-1.18)
**Adjusted Model**					
**PBF**	0.59*** (0.57-0.61)	0.45*** (0.42-0.47)	0.63***(0.59-0.67)	0.84***(0.77-0.91)	1.00 (0.86-1.15)
**Sex**					
Female	1 (base)	1 (base)	1 (base)	1 (base)	1 (base)
Male	1.21*** (1.17-1.25)	0.98 (0.94-1.03)	1.17*** (1.09-1.25)	1.47*** (1.36-1.59)	2.26*** (2.07-2.47)
**Age**					
Adolescents and young people[c]	1 (base)	1 (base)	1 (base)	1 (base)	1 (base)
Adults[d]	2.02*** (1.93-2.10)	1.97*** (1.86-2.07)	1.96*** (1.79-2.15)	2.17***(1.92-2.44)	2.63***(2.28-3.04)
Older people[e]	0.32*** (0.28-0.37)	0.22** (0.16-0.30)	0.28*** (0.21-0.39)	0.47***(0.32-0.69)	0.63** (0.48-0.84)
**Race/ethnicity**					
White	1 (base)	1 (base)	1 (base)	1 (base)	1 (base)
Mixed-race	1.24*** (1.19-1.29)	1.18** (1.11-1.25)	1.20*** (1.10-1.30)	1.33***(1.20-1.48)	1.22*** (1.11-1.35)
Black	1.66*** (1.57-1.75)	1.57*** (1.45-1.70)	1.73*** (1.56-1.92)	1.82***(1.59-2.08)	1.54*** (1.32-1.79)
Indigenous	1.36* (1.00-1.85)	1.35 (0.92-1.98)	1.01 (0.54-1.89)	1.05 (0.41-2.67)	3.32** (1.52-7.26)
**Education**					
More than high school	1 (base)	1 (base)	1 (base)	1 (base)	1 (base)
High school	1.04 (0.91-1.18)	1.12 (0.81-1.54)	0.94 (0.72-1.24)	1.12 (0.82-1.54)	0.85 (0.68-1.07)
Elementary school	1.40*** (1.23-1.60)	1.54** (1.12-2.10)	1.35** (1.04-1.76)	1.70** (1.24-2.33)	0.89 (0.71-1.12)
Attended pre-school	0.91 (0.72-1.15)	0.98 (0.65-1.49)	0.81 (0.50-1.32)	1.24 (0.70-2.19)	0.46** (0.26-0.83)
Never attended school	1.28*** (1.11-1.47)	1.50** (1.09-2.06)	1.12 (0.84-1.49)	1.89*** (1.34-2.66)	0.69** (0.51-0.92)
**Wealth[f]**					
Level 1 (More wealth)	1 (base)	x	x	x	x
Level 2	0.98 (0.89-1.08)	x	x	x	x
Level 3	1.16* (1.05-1.28)	x	x	x	x
Level 4	1.57*** (1.42-1.73)	x	x	x	x
Level 5 (Lower wealth)	2.13*** (1.93-2.36)	x	x	x	x
**Water supply**					
Public network	1 (base)	1 (base)	1 (base)	1 (base)	1 (base)
Other[g]	1.01 (0.96-1.06)	1.00 (0.94-1.07)	1.00 (0.91-1.10)	0.99 (0.87-1.14)	0.99 (0.83-1.17)
**Housing material (brick)**					
Yes	1 (base)	1 (base)	1 (base)	1 (base)	1 (base)
No[h]	1.24*** (1.19-1.30)	1.23*** (1.16-1.31)	1.26*** (1.15-1.38)	1.23*** (1.09-1.38)	1.15* (1.00-1.32)
**Lighting**					
Electricity	1 (base)	1 (base)	1 (base)	1 (base)	1 (base)
Non-electric[i]	1.31*** (1.25-1.38)	1.39*** (1.30-1.48)	1.30*** (1.18-1.44)	1.19** (1.05-1.35)	1.09 (0.89-1.32)
**Region**					
North	1 (base)	1 (base)	1 (base)	1 (base)	1 (base)

Northeast	1.19*** (1.11-1.26)	1.32** (1.21-1.44)	1.15* (1.00-1.32)	1.14 (0.95-1.36)	1.04 (0.84-1.28)
Southeast	1.19*** (1.11-1.27)	1.40*** (1.27-1.54)	1.15* (1.00-1.33)	1.05 (0.88-1.26)	0.84 (0.68-1.02)
South	1.52*** (1.40-1.65)	1.71*** (1.52-1.92)	1.54*** (1.31-1.81)	1.26** (1.04-1.54)	1.01 (0.81-1.26)
Central-west	1.22*** (1.12-1.34)	1.30 (1.14-1.47)	1.32** (1.11-1.57)	0.99 (0.80-1.23)	0.88 (0.70-1.12)
**Area of residence**					
Rural	1 (base)	1 (base)	1 (base)	1 (base)	1 (base)
Urban	1.89*** (1.77-2.03)	1.98*** (1.81-2.16)	1.77*** (1.54-2.04)	1.84*** (1.53-2.23)	2.32*** (1.77-3.05)
**Mun. average AIDS incidence rate[j]**	1.03*** (1.02-1.03)	1.03*** (1.03-1.03)	1.02*** (1.02-1.03)	1.02***(1.02-1.03)	1.02*** (1.02-1.03)
**Inadequate sanitation[l]**	0.99*** (0.99-0.99)	0.99** (0.99-0.99)	0.99 (0.99-1.00)	0.99 (0.98-1.00)	0.98** (0.98-0.99)
**Unemployment rate (%)[m]**	1.01*** (1.01-1.02)	1.00* (1.00-1.01)	1.02*** (1.00-1.03)	1.01 (0.99-1.03)	1.00 (0.98-1.02)
**Doctors per 1,000 inhabitants[n]**	1.02 (0.99-1.05)	1.03 (0.99-1.08)	1.02 (0.97-1.08)	0.97 (0.90-1.03)	1.07** (1.01-1.14)
**Nurses per 1,000 inhabitants[n]**	0.85*** (0.78-0.92)	0.99 (0.87-1.13)	0.93 (0.75-1.14)	0.96 (0.80-1.16)	0.78** (0.65-0.93)
**Hospital beds per 1,000 inhabitants[n]**	0.99 (0.98-1.01)	0.99*** (0.99-0.99)	0.99*** (0.99-0.99)	0.99 (0.99-1.00)	0.99** (0.99-1.00)
**Individual`s year of entry into the cohort**	yes	yes	yes	yes	yes
Obs.:	19,577,629	5,503,505	3,868,703	5,051,629	5,158,593

Notes: *** p-value <0.001; **p-value <0.01; *p-value <0.05. [a] Incidence rate ratios. [b] Confidence interval. [c] Aged between 13 and 24. [d] Aged between 25 and 64. [e] Aged 65 or older. [f] Measured by capita expenses proportional to the baseline minimum wage (MW). Level 1 (More wealth): "1 or more". Level 2: "0.5 to 1". Level 3: "0.25 to 0.49". Level 4: "0< to 0.24". Level 5 (Lower wealth): "Nothing declared". [g] Water supply: Other – well, spring, and others. [h] Housing Material: No – Coated clay, uncoated clay, wood, and others. [i] Lighting: Non-electric – No meter, lamps, candles, and others. [j] Average rates for the period (2007-2015) by municipality. [l] % of the municipal population with inadequate baseline sanitation. [m] Baseline municipal unemployment rate. [n] Per 1,000 inhabitants of the baseline municipality. [o] Quartile 1: 0% a 0.1%. [p] Quartile 2: 0.1%< a 18.5%. [q] Quartile 3: 18.5% < a 56.5%. [r] Quartile 4: 56.5<. All statistical tests used where two-sided and, where appropriate, adjustments were made for multiple comparisons.

Table S34. Estimates of the average effect of the *Programa Bolsa Famlília* (PBF) adjusted Poisson model (with robust standard errors) on AIDS mortality rate in Brazil, 2007-2015.

Models	Mortality (IRR[a] – CI[b] 95%)				
	Total	Wealth[f]			
		Quartile 1[o]	Quartile 2[p]	Quartile 3[q]	Quartile 4[r]
**Unadjusted Model**					
**PBF**	0.68*** (0.64-0.72)	0.45*** (0.41-0.48)	0.67*** (0.60-0.75)	0.85* (0.73-0.98)	0.99 (0.78-1.27)
**PBF**	0.61*** (0.57-0.64)	0.46*** (0.42-0.49)	0.65***(0.58-0.73)	0.84** (0.72-0.96)	0.98 (0.77-1.25)
**Sex**					
Female	1 (base)	1 (base)	1 (base)	1 (base)	1 (base)
Male	1.35*** (1.27-1.42)	1.15*** (1.06-1.25)	1.34*** (1.20-1.50)	1.52*** (1.32-1.74)	2.54*** (2.17-2.96)
**Age**					
Adolescents and young people[c]	1 (base)	1 (base)	1 (base)	1 (base)	1 (base)
Adults[d]	3.50*** (3.20-3.82)	3.25*** (2.91-3.62)	3,75*** (3.07-4.56)	3.83*** (2.97-4.95)	6.56*** (4.49-9.58)
Older people[e]	0.78* (0.64-0.96)	0.53** (0.36-0.80)	0.68 (0.16-1.21)	1.21 (0.68-2.17)	2.18*** (1.35-3.52)
**Race/ethnicity**					
White	1 (base)	1 (base)	1 (base)	1 (base)	1 (base)
Mixed-race	1.24*** (1.15-1.32)	1.15** (1.04-1.27)	1.21** (1.06-1.39)	1.33** (1.11-1.60)	1.22* (1.02-1.45)
Black	1.78*** (1.64-1.94)	1.64*** (1.45-1.86)	1.98*** (1.68-2.33)	2.04*** (1.64-2.53)	1.47** (1.13-1.90)
Indigenous	0.89 (0.49-1.62)	0.86 (0.39-1.89)	0.44 (0.16-1.21)	0.99 (0.30-3.31)	3.87* (1.30-11.5)
**Education**					
More than high school	1 (base)	1 (base)	1 (base)	1 (base)	1 (base)
High school	1.45** (1.07-1.94)	1.38 (0.70-2.72)	0.97 (0.58-1.61)	2.12* (1.04-4.33)	1.66* (1.02-2.71)
Elementary school	2.61*** (1.96-3.49)	2.52** (1.30-4.87)	1.81** (1.12-2.94)	4.36*** (2.16-8.80)	2.41*** (1.50-3.87)
Attended pre-school	1.20 (0.75-1.91)	1.53 (0.67-3.49)	0.75 (0.28-1.99)	1.98 (0.68-5.78)	0.34 (0.07-1.58)
Never attended school	2.74*** (2.03-3.70)	2.83** (1.45-5.51)	1.76* (1.06-2.93)	4.99*** (2.39-10.4)	2.18** (1.28-3.72)
**Wealth[f]**					
Level 1 (More wealth)	1 (base)	x	x	x	x
Level 2	0.91 (076-1.08)	x	x	x	x
Level 3	1.22* (1.03-1.45)	x	x	x	x
Level 4	1.55*** (1.31-1.84)	x	x	x	x
Level 5 (Lower wealth)	2.33*** (1.95-2.79)	x	x	x	x
**Water supply**					
Public network	1 (base)	1 (base)	1 (base)	1 (base)	1 (base)
Other[g]	1.07 (0.98-1.16)	1.07 (0.96-1.19)	1.06 (0.91-1.24)	0.96 (0.75-1.23)	1.04 (0.77-1.42)
**Housing material (brick)**					
Yes	1 (base)	1 (base)	1 (base)	1 (base)	1 (base)
No[h]	1.33*** (1.24-1.44)	1.32*** (1.19-1.47)	1.44*** (1.23-1.68)	1.38** (1.13-1.69)	0.97 (0.75-1.25)
**Lighting**					
Electricity	1 (base)	1 (base)	1 (base)	1 (base)	1 (base)
Non-electric[i]	1.37*** (1.26-1.48)	1.45*** (1.30-1.62)	1.30** (1.11-1.53)	1.31** (1.06-1.63)	1.35 (0.99-1.85)
**Region**					
North	1 (base)	1 (base)	1 (base)	1 (base)	1 (base)
Northeast	1.03 (0.92-1.16)	1.22** (1.05-1.42)	1.00 (0.78-1.28)	0.89 (0.64-1.25)	0.70 (0.48-1.02)
Southeast	1.38*** (1.22-1.55)	1.55*** (1.32-1.82)	1.34* (1.05-1.70)	1.15 (0.83-1.59)	0.72 (0.51-1.01)

South	1.40*** (1.22-1.62)	1.73*** (1.41-2.12)	1.47** (1.11-1.96)	0.96 (0.65-1.42)	0.76 (0.51-1.13)
Central-west	1.19* (1.02-1.40)	1.29** (1.03-1.61)	1.31** (0.97-1.77)	0.85 (0.57-1.26)	0.67 (0.44-1.03)
**Area of residence**					
Rural	1 (base)	1 (base)	1 (base)	1 (base)	1 (base)
Urban	2.21*** (1.97-2.49)	2.32*** (2.00-2.69)	2.07*** (1.63-2.62)	2.18*** (1.80-3.69)	2.27** (1.40-3.67)
**Mun. average AIDS mortality rate**[j]	1.10*** (1.09-1.10)	1.10*** (1.09-1.11)	1.09*** (1.08-1.10)	1.10*** (1.08-1.11)	1.09*** (1.07-1.10)
**Inadequate sanitation**[l]	0.99*** (0.98-0.99)	0.99** (0.99-0.99)	0.99 (0.99-1.00)	0.98* (0.97-0.99)	0.98 (0.99-1.07)
**Unemployment rate (%)**[m]	1.02*** (1.01-1.03)	1.00* (0.99-1.02)	1.02** (1.00-1.05)	1.04** (1.01-1.07)	1.03 (0.99-1.07)
**Doctors per 1,000 inhabitants**[n]	1.05* (1.00-1.10)	1.08** (1.00-1.15)	1.03 (0.64-1.31)	0.87** (0.78-0.97)	1.16** (1.05-1.29)
**Nurses per 1,000 inhabitants**[n]	0.86 (0.74-1.00)	0.82 (0.65-1.02)	0.92 (0.64-1.14)	1.15 (0.84-1.57)	0.74 (0.54-1.00)
**Hospital beds per 1,000 inhabitants**[n]	0.99 (0.99-1.00)	0.99 (0.99-1.00)	0.99* (0.99-1.00)	1.00 (0.99-1.00)	0.99** (0.99-0.99)
**Individual`s year of entry into the cohort**	yes	yes	yes	yes	yes
Obs.:	19,577,649	5,503,510	3,868,708	5,051,636	5,158,596

Notes: *** p-value <0.001; **p-value <0.01; *p-value <0.05. [a] Incidence rate ratios. [b] Confidence interval. [c] Aged between 13 and 24. [d] Aged between 25 and 64. [e] Aged 65 or older. [f] Measured by capita expenses proportional to the baseline minimum wage (MW). Level 1 (More wealth): “1 or more”. Level 2: “0.5 to 1”. Level 3: “0.25 to 0.49”. Level 4: “0< to 0.24”. Level 5 (Lower wealth): “Nothing declared”.. [g] Water supply: Other – well, spring, and others. [h] Housing Material: No – Coated clay, uncoated clay, wood, and others. [i] Lighting: Non-electric – No meter, lamps, candles, and others. [j] Average rates for the period (2007-2015) by municipality. [l]% of the municipal population with inadequate baseline sanitation. [m] Baseline municipal unemployment rate. [n] Per 1,000 inhabitants of the baseline municipality. [o] Quartile 1: 0% a 0.1%. [p] Quartile 2: 0.1%< a 18.5%. [q] Quartile 3: 18.5% < a 56.5%. [r] Quartile 4: 56.5<. All statistical tests used where two-sided and, where appropriate, adjustments were made for multiple comparisons.

Table S35. Estimates of the average effect of the *Programa Bolsa Famlília* (PBF) adjusted Poisson model (with robust standard errors) on AIDS case-fatality rate in Brazil, 2007-2015.

Models	Case-fatality rate (IRR[a] – CI[b] 95%)				
	Total	Wealth[f]			
		Quartile 1[o]	Quartile 2[p]	Quartile 3[q]	Quartile 4[r]
**Unadjusted Model**					
**PBF**	0.74*** (0.66-0.83)	0.63*** (0.52-0.75)	0.76* (0.61-0.94)	1.02 (0.79-1.32)	0.79 (0.53-1.17)
**PBF**	0.75*** (0.66-0.85)	0.63*** (0.51-0.76)	0.77* (0.60-0.98)	1.05 (0.80-1.38)	0.93 (0.60-1.42)
**Sex**					
Female	1 (base)	1 (base)	1 (base)	1 (base)	1 (base)
Male	1.27*** (1.13-1.43)	1.17 (0.99-1.38)	1.68*** (1.32-2.14)	1.24 (0.93-1.66)	1.89*** (1.33-2.70)
**Age**					
Adolescents and young people[c]	1 (base)	1 (base)	1 (base)	1 (base)	1 (base)
Adults[d]	1.69*** (1.40-2.04)	1.51*** (1.20-1.89)	2.44*** (1.60-3.71)	1.74* (1.01-3.00)	1.74 (0.94-3.24)
Older people[e]	2.84*** (1.78-4.54)	3.32** (1.38-7.96)	5.00** (1.75-14.2)	3.01* (1.08-8.36)	2.79* (1.14-6.84)
**Race/ethnicity**					
White	1 (base)	1 (base)	1 (base)	1 (base)	1 (base)
Mixed-race	1.07 (0.92-1.25)	1.01 (0.81-1.25)	0.94 (0.70-1.26)	1.25 (0.86-1.82)	1.00 (0.69-1.44)
Black	1.01 (0.83-1.22)	0.99 (0.75-1.29)	1.09 (0.76-1.56)	1.07 (0.66-1.75)	0.71 (0.39-1.30)
Indigenous	0.57 (0.20-1.61)	0.35 (0.07-1.71)	0.40 (0.04-3.49)	76.4*** (25.8-226)	0.00*** (0.00-0.00)
**Education**					
More than high school	1 (base)	1 (base)	1 (base)	1 (base)	1 (base)
High school	1.95* (1.06-3.58)	1.69 (0.39-7.19)	3.70* (1.17-11.7)	0.99 (0.36-2.73)	2.13 (0.86-5.24)
Elementary school	2.77** (1.53-5.02)	2.33 (0.56-9.66)	4.14** (1.35-12.6)	1.40 (0.52-3.74)	3.40** (1.40-8.25)
Attended pre-school	1.00 (0.35-2.78)	1.39 (0.23-8.31)	1.32 (0.19-8.97)	0.52 (0.06-4.56)	-
Never attended school	3.62*** (1.95-6.74)	3.32 (0.78-14.02)	4.69** (1.41-15.5)	2.07 (0.71-6.08)	4.24** (1.45-12.33)
**Wealth[f]**					
Level 1 (More wealth)	1 (base)	x	x	x	x
Level 2	1.22 (0.80-1.87)	x	x	x	x
Level 3	1.61* (1.06-2.43)	x	x	x	x
Level 4	1.31 (0.87-1.98)	x	x	x	x
Level 5 (Lower wealth)	1.56* (1.02-2.40)	x	x	x	x
**AIDS treatment**					
Yes	1 (base)	1 (base)	1 (base)	1 (base)	1 (base)
No	2.67*** (2.32-3.08)	2.44*** (2.00-2.97)	2.75*** (2.08-3.65)	3.63*** (2.63-5.03)	2.40*** (1.61-3.57)
**Water supply**					
Public network	1 (base)	1 (base)	1 (base)	1 (base)	1 (base)
Other[g]	1.03 (0.87-1.23)	1.10 (0.88-1.37)	1.03 (0.74-1.44)	0.75 (0.46-1.21)	1.28 (0.65-2.51)
**Housing material (brick)**					
Yes	1 (base)	1 (base)	1 (base)	1 (base)	1 (base)
No[h]	1.18* (1.00-1.39)	1.19 (0.94-1.50)	1.39* (1.01-1.92)	1.18 (0.76-1.83)	0.71 (0.41-1.22)
**Lighting**					
Electricity	1 (base)	1 (base)	1 (base)	1 (base)	1 (base)
Non-electric[i]	1.21* (1.02-1.43)	1.25* (1.00-1.57)	0.91 (0.66-1.27)	1.65** (1.11-2.47)	1.32 (0.69-2.51)
**Region**					

North	1 (base)	1 (base)	1 (base)	1 (base)	1 (base)
Northeast	1.06 (0.81-1.37)	1.35 (0.93-1.95)	0.61 (0.37-1.02)	1.30 (0.66-2.57)	0.53 (0.25-1.09)
Southeast	1.10 (0.83-1.45)	1.37 (0.89-2.08)	0.63 (0.37-1.08)	1.44 (0.72-2.88)	0.55 (0.25-1.20)
South	0.95 (0.70-1.30)	1.13 (0.73-1.79)	0.65 (0.37-1.12)	1.28 (0.57-2.86)	0.56 (0.23-1.37)
Central-west	1.20 (0.85-1.68)	1.49 (0.88-2.53)	0.73 (0.38-1.40)	1.92 (0.84-4.38)	0.78 (0.34-1.80)
**Area of residence**					
Rural	1 (base)	1 (base)	1 (base)	1 (base)	1 (base)
Urban	1.06 (0.79-1.42)	1.12 (0.79-1.60)	0.83 (0.47-1.45)	0.99 (0.38-2.56)	1.98 (0.35-11.2)
**Mun. average AIDS case-fatality rate**[j]	1.01*** (1.00-1.02)	1.01** (1.00-1.02)	1.02** (1.00-1.04)	1.00 (0.99-1.02)	1.04** (1.01-1.06)
**Inadequate sanitation**[l]	1.00 (0.99-1.01)	1.00 (0.99-1.01)	0.99 (0.96-1.01)	1.00 (0.97-1.02)	1.02 (0.98-1.06)
**Unemployment rate (%)**[m]	0.98 (0.96-1.01)	0.97 (0.93-1.00)	1.01 (0.95-1.07)	1.03 (0.95-1.11)	0.99 (0.90-1.09)
**Doctors per 1,000 inhabitants**[n]	0.85** (0.76-0.95)	0.86 (0.73-1.02)	0.86 (0.70-1.06)	0.81 (0.62-1.06)	0.90 (0.66-1.22)
**Nurses per 1,000 inhabitants**[n]	1.71** (1.23-2.36)	1.55 (0.94-2.57)	1.48 (0.74-2.97)	1.27 (0.60-2.69)	2.35* (1.02-5.40)
**Hospital beds per 1,000 inhabitants**[n]	0.97 (0.92-1.01)	0.96 (0.91-1.01)	1.00 (0.86-1.15)	1.04 (0.89-1.22)	0.99 (0.85-1.15)
**Individual`s year of entry into the cohort**	yes	yes	yes	yes	yes
Obs.:	9,965	4,059	2,398	1,880	1,629

Notes: *** p-value <0.001; **p-value <0.01; *p-value <0.05. [a] Incidence rate ratios. [b] Confidence interval. [c] Aged between 13 and 24. [d] Aged between 25 and 64. [e] Aged 65 or older. [f] Measured by capita expenses proportional to the baseline minimum wage (MW). Level 1 (More wealth): "1 or more". Level 2: "0.5 to 1". Level 3: "0.25 to 0.49". Level 4: "0< to 0.24". Level 5 (Lower wealth): "Nothing declared".. [g] Water supply: Other – well, spring, and others. [h] Housing Material: No – Coated clay, uncoated clay, wood, and others. [i] Lighting: Non-electric – No meter, lamps, candles, and others. [j] Average rates for the period (2007-2015) by municipality. [l] % of the municipal population with inadequate baseline sanitation. [m] Baseline municipal unemployment rate. [n] Per 1,000 inhabitants of the baseline municipality. [o] Quartile 1: 0% a 0.1%. [p] Quartile 2: 0.1%< a 18.5%. [q] Quartile 3: 18.5% < a 56.5%. [r] Quartile 4: 56.5<. All statistical tests used where two-sided and, where appropriate, adjustments were made for multiple comparisons.

Table S36. Estimates of the average effect of the *Programa Bolsa Famlília* (PBF), adjusted Poisson model (with robust standard error), on AIDS incidence, mortality and case-fatality rate in Brazil, 2007-2015 – by subpopulation of sex.

Models	Female (IRR[a] – IC[b] 95%)			Male (IRR[a] – IC[b] 95%)		
	Incidence	Mortality	Case-Fatality	Incidence	Mortality	Case-Fatality
**Unadjusted Model**						
**PBF**	0.72*** (0.69-0.76)	0.71*** (0.65-0.76)	0.63*** (0.53-0.74)	0.64*** (0.61-0.67)	0.68*** (0.63-0.74)	0.88 (0.75-1.02)
**Adjusted Model**						
**PBF**	0.60*** (0.57-0.63)	0.58*** (0.53-0.62)	0.64*** (0.53-0.78)	0.60*** (0.57-0.63)	0.64*** (0.59-0.70)	0.87 (0.74-1.03)
**Race/ethnicity**						
White	1 (base)	1 (base)	1 (base)	1 (base)	1 (base)	1 (base)
Mixed-race	1.30*** (1.23-1.38)	1.28*** (1.16-1.42)	1.13 (0.91-1.39)	1.15*** (1.09-1.22)	1.19*** (1.08-1.31)	1.07 (0.86-1.34)
Black	1.83*** (1.70-1.97)	2.00*** (1.77-2.26)	1.12 (0.85-1.48)	1.46*** (1.35-1.58)	1.56*** (1.38-1.77)	1.00 (0.75-1.31)
Indigenous	1.05 (0.67-1.64)	1.22 (0.57-2.61)	0.42 (0.08-2.18)	1.63* (1.07-2.49)	0.54 (0.21-1.35)	0.80 (0.22-2.93)
**Age**						
Adolescents and Youth people[c]	1 (base)	1 (base)	1 (base)	1 (base)	1 (base)	1 (base)
Adults[d]	1.95*** (1.84-2.07)	3.03*** (2.69-3.42)	1.62*** (1.25-2.09)	2.14*** (2.01-2.27)	4.11*** (3.60-4.69)	1.70*** (1.30-2.22)
Older people[e]	0.22*** (0.17-0.27)	0.44*** (0.31-0.61)	2.59 (0.96-6.95)	0.45*** (0.38-0.53)	1.22 (0.95-1.59)	3.22* (1.84-5.63)
**Education**						
More than High School	1 (base)	1 (base)	1 (base)	1 (base)	1 (base)	1 (base)
High school	1.60*** (1.27-2.02)	1.96* (1.16-3.30)	2.84* (1.18-6.79)	0.71*** (0.60-0.84)	1.11 (0.77-1.58)	1.55 (0.77-3.12)
Elementary school	2.64*** (2.10-3.32)	4.02*** (2.40-6.72)	3.27* (1.39-7.69)	0.77** (0.66-0.90)	1.72** (1.22-2.43)	2.56* (1.29-5.07)
Attended pre-school	1.62** (1.13-2.33)	2.10 (1.00-4.39)	2.27 (0.59-8.76)	0.50*** (0.37-0.70)	0.70 (0.38-1.29)	0.56 (0.12-2.63)
Never attended school	2.39** (1.88-3.03)	4.49*** (2.65-7.63)	4.74** (1.95-11.51)	0.70*** (0.59-0.83)	1.70** (1.18-2.44)	2.87* (1.38-5.96)
**Wealth[f]**						
Level 1 (More wealth)	1 (base)	1 (base)	1 (base)	1 (base)	1 (base)	1 (base)
Level 2	1.11 (0.94-1.32)	0.96 (0.72-1.29)	1.00 (0.48-2.08)	0.95 (0.84-1.07)	0.89 (0.72-1.10)	1.30 (0.79-2.13)
Level 3	1.42*** (1.21-1.67)	1.43* (1.08-1.89)	1.71 (0.87-3.38)	1.10 (0.97-1.24)	1.15 (0.93-1.42)	1.36 (0.83-2.25)
Level 4	1.89*** (1.61-2.23)	1.62** (1.22-2.15)	1.06 (0.54-2.06)	1.50*** (1.32-1.70)	1.62*** (1.31-2.01)	1.49 (0.90-2.46)
Level 5 (Lower wealth)	2.51*** (2.12-2.97)	2.45*** (1.83-3.29)	1.47 (0.74-2.91)	2.15*** (1.88-2.45)	2.52*** (2.02-3.16)	1.42 (0.83-2.40)
**AIDS Treatment**						
Yes	x	x	1 (base)	x	x	1 (base)
No	x	x	2.41*** (1.96-2.96)	x	x	2.89*** (2.38-3.50)
**Water supply**						
Public network	1 (base)	1 (base)	1 (base)	1 (base)	1 (base)	1 (base)
Others[g]	0.98 (0.92-1.05)	0.98 (0.87-1.10)	0.97 (0.76-1.25)	1.04 (0.97-1.12)	1.16* (1.03-1.30)	1.05 (0.83-1.33)
**Housing material (Brick)**						
Yes	1 (base)	1 (base)	1 (base)	1 (base)	1 (base)	1 (base)
No[h]	1.20*** (1.13-1.28)	1.25*** (1.12-1.39)	0.95 (0.75-1.20)	1.27*** (1.19-1.36)	1.40*** (1.26-1.55)	1.35* (1.07-1.70)
**Lighting**						
Electricity	1 (base)	1 (base)	1 (base)	1 (base)	1 (base)	1 (base)
Non-electric[i]	1.41*** (1.32-1.51)	1.56*** (1.39-1.74)	1.19 (0.94-1.50)	1.20*** (1.12-1.30)	1.18* (1.05-1.33)	1.17 (0.92-1.48)
**Region**						
North	1 (base)	1 (base)	1 (base)	1 (base)	1 (base)	1 (base)
Northeast	1.09* (1.00-1.19)	0.87 (0.74-1.02)	0.86 (0.61-1.22)	1.36*** (1.24-1.50)	1.25* (1.06-1.48)	1.17 (0.79-1.74)
Southeast	1.20*** (1.09-1.32)	1.36*** (1.16-1.59)	1.06 (0.72-1.56)	1.27*** (1.14-1.41)	1.41*** (1.19-1.68)	0.96 (0.63-1.45)

South	1.64*** (1.47-1.84)	1.31** (1.07-1.60)	0.69 (0.45-1.06)	1.35*** (1.20-1.52)	1.52*** (1.24-1.86)	1.15 (0.74-1.79)
Central-west	1.27*** (1.13-1.43)	1.16 (0.94-1.43)	1.22 (0.77-1.93)	1.19** (1.05-1.36)	1.23 (0.99-1.54)	1.02 (0.62-1.68)
**Area of residence**						
Rural	1 (base)	1 (base)	1 (base)	1 (base)	1 (base)	1 (base)
Urban	1.72*** (1.57-1.90)	1.95*** (1.65-2.32)	0.84 (0.56-1.25)	2.07*** (1.88-2.27)	2.42*** (2.07-2.84)	1.17 (0.77-1.76)
**Average AIDS incidence rate**[j]	1.03*** (1.02-1.03)	x	x	1.03*** (1.02-1.03)	x	x
**Average AIDS mortality rate**[j]	x	1.10*** (1.08-1.10)	x	x	1.10*** (1.09-1.11)	x
**Average AIDS case-fatality rate**[j]	x	x	1.01*** (1.00-1.02)	x	x	1.02*** (1.01-1.03)
**Inadequate sanitation**[l]	0.99** (0.99-0.99)	0.99* (0.98-0.99)	1.00 (0.99-1.02)	0.99*** (0.99-0.99)	0.99** (0.98-0.99)	0.99 (0.98-1.01)
**Unemployment rate (%)**[m]	1.01*** (1.00-1.02)	1.01* (1.00-1.03)	0.98 (0.95-1.02)	1.01*** (1.00-1.02)	1.03*** (1.01-1.04)	0.99 (0.95-1.03)
**Doctors per 1000 inhabitants**[n]	0.99 (0.95-1.03)	1.02 (0.96-1.09)	0.86 (0.73-1.01)	1.06** (1.02-1.10)	1.07* (1.01-1.14)	0.85* (0.74-0.98)
**Nurses per 1000 inhabitants**[n]	0.97 (0.86-1.10)	0.86 (0.69-1.08)	1.89* (1.16-3.07)	0.89 (0.79-1.01)	0.87 (0.72-1.06)	1.67* (1.10-2.52)
**Hospital beds per 1000 inhabitants**[n]	0.99*** (0.99-0.99)	0.99** (0.99-0.99)	0.96 (0.89-1.03)	0.99 (0.99-1.00)	0.99 (0.99-1.00)	0.97 (0.92-1.02)
**Year of entry of individual in cohort**	yes	yes	yes	yes	yes	yes
Obs.:	11,309,111	11,309,119	5,289	8,268,518	8,268,530	4,676

Notes: *** p-value <0,001; **p-valor <0,01; *p-valor <0,05. [a] Incidence Rate Ratios. [b] Confidence Interval. [c] Aged between 13 and 24. [d] Aged between 25 and 64. [e] Aged 65 or older. [f] Measured by capita expenses proportional to the baseline minimum wage (MW). Level 1 (More wealth): "1 or more". Level 2: "0.5 to 1". Level 3: "0.25 to 0.49". Level 4: "0< to 0.24". Level 5 (Lower wealth): "Nothing declared". [g] Water supply: Other – well, spring, and others. [h] Housing Material: No – Coated clay, uncoated clay, wood, and others. [i] Lighting: Non-electric – No meter, lamps, candles, and others. [j] Average rates for the period (2007-2015) by municipality. [l] % of the municipal population with inadequate baseline sanitation. [m] Baseline municipal unemployment rate. [n] Per 1,000 inhabitants of the baseline municipality. All statistical tests used where two-sided and, where appropriate, adjustments were made for multiple comparisons.

Table S37. Estimates of the average effect of the *Programa Bolsa Famlília* (PBF), adjusted Poisson model (with robust standard error), on AIDS incidence, mortality and case-fatality rate in Brazil, 2007-2015 – by age subpopulation.

Models	Adolescents and Youth people (IRR[a] – IC[b] 95%)			Adults (IRR[a] – IC[b] 95%)			Older people (IRR[a] – IC[b] 95%)		
	Incidence	Mortality	Case-Fatality	Incidence	Mortality	Case-Fatality	Incidence	Mortality	Case-Fatality
**Unadjusted Model**									
**PBF**	0.52*** (0.48-0.56)	0.55*** (0.46-0.64)	0.67*** (0.47-0.96)	0.66*** (0.63-0.68)	0.66*** (0.62-0.69)	0.76*** (0.67-0.86)	1.24 (0.83-1.87)	1.62 (0.97-2.82)	1.51 (0.41-5.50)
**Adjusted Model**									
**PBF**	0.47*** (0.44-0.51)	0.46*** (0.39-0.54)	0.72 (0.46-1.12)	0.62*** (0.60-0.64)	0.61*** (0.57-0.65)	0.75*** (0.65-0.85)	1.18 (0.78-1.78)	1.53 (0.91-2.59)	0.05 (0.0-4.10)
**Sex**									
Females	1 (base)	1 (base)	1 (base)	1 (base)	1 (base)	1 (base)	1 (base)	1 (base)	1 (base)
Male	1.06 (0.99-1.15)	0.95 (0.81-1.12)	1.29 (0.88-1.90)	1.22*** (1.18-1.27)	1.36*** (1.29-1.45)	1.26*** (1.11-1.43)	2.20*** (1.76-2.76)	2.48*** (1.81-3.40)	24.75** (3.1-197)
**Race/ethnicity**									
White	1 (base)	1 (base)	1 (base)	1 (base)	1 (base)	1 (base)	1 (base)	1 (base)	1 (base)
Mixed-race	1.16** (1.05-1.28)	1.50*** (1.20-1.88)	1.89** (1.17-3.05)	1.25*** (1.19-1.31)	1.22*** (1.13-1.31)	1.01 (0.86-1.19)	1.39* (1.07-1.81)	1.25 (0.85-1.83)	1.37 (0.10-18.5)
Black	1.52*** (1.33-1.74)	2.55*** (1.93-3.35)	2.58** (1.38-4.81)	1.67*** (1.58-1.78)	1.74*** (1.59-1.91)	0.92 (0.75-1.14)	2.09*** (1.46-2.98)	2.11* (1.31-3.40)	0.37 (0.05-2.55)
Indigenous	1.55 (0.86-2.80)	1.46 (0.36-5.92)		1.21 (0.84-1.73)	0.82 (0.42-1.58)	0.49 (0.15-1.60)	5.26** (1.53-18.01)	1.62 (0.39-6.66)	0.00*** (0.0-0.0)
**Education**									
More than High School	1 (base)	1 (base)	1 (base)	1 (base)	1 (base)	1 (base)	1 (base)	1 (base)	1 (base)
High school	0.73 (0.52-1.04)	0.82 (0.32-2.68)	4.16 (0.51-33.8)	1.11 (0.96-1.29)	1.60* (1.19-2.14)	1.88* (1.04-3.41)	0.79 (0.27-2.28)	5.13 (0.66-39.7)	9.15*** (9.4-88.6)
Elementary school	0.94 (0.66-1.32)	1.71 (0.60-4.86)	6.54 (0.81-52.5)	1.54*** (1.34-1.78)	2.90*** (1.18-3.86)	2.69** (1.51-4.81)	0.85 (0.33-2.15)	3.72 (0.51-26.7)	4.15* (1.85-9.26)
Attended pre-school	0.58 (0.30-1.17)	1.08 (0.23-5.04)	0.00*** (0.0-0.0)	1.00 (0.78-1.30)	1.39 (0.84-2.29)	0.87 (0.29-2.61)	1.10 (0.34-3.50)	3.45 (0.35-33.2)	
Never attended school	1.07 (0.72-1.57)	2.53 (0.85-7.56)	12.0* (1.2-114.8)	1.40*** (1.20-1.63)	3.11*** (2.31-4.18)	3.69*** (2.01-6.77)	0.91 (0.35-2.35)	3.54 (0.48-25.8)	
**Wealth[c]**									
Level 1 (More wealth)	1 (base)	1 (base)	1 (base)	1 (base)	1 (base)	1 (base)	1 (base)	1 (base)	
Level 2	0.91 (0.67-1.24)	0.93 (0.32-2.68)	11.54** (1.99-66.6)	0.99 (0.89-1.10)	0.92 (0.76-1.10)	1.09 (0.69-1.69)	1.15 (0.80-1.67)	0.89 (0.55-1.45)	1 (base)
Level 3	1.29 (0.96-1.73)	1.71 (0.60-4.86)	6.68* (1.18-37.5)	1.14* (1.02-1.26)	1.20* (1.00-1.43)	1.52 (0.99-2.33)	1.52* (1.00-2.31)	1.18 (0.67-2.07)	0.88 (0.10-7.54)
Level 4	1.70*** (1.27-2.27)	1.08 (0.23-5.04)	5.52* (1.01-30.1))	1.53*** (1.37-1.70)	1.54*** (1.29-1.85)	1.22 (0.80-1.88)	2.03** (1.23-3.36)	1.94 (0.99-3.79)	4.65 (0.64-33.7)
Level 5 (Lower wealth)	1.93*** (1.43-2.60)	2.53 (0.85-7.56)	6.51* (1.17-36.2)	2.14*** (1.92-2.40)	2.37*** (1.96-2.86)	1.45 (0.93-2.27)	2.99*** (1.81-5.95)	2.99** (1.57-5.68)	0.45 (0.4-5.18)
**AIDS Treatment**									
Yes	x	x	1 (base)	x	x	1 (base)	x	x	1 (base)
No	x	x	2.55*** (1.71-3.80)	x	x	2.73*** (2.34-3.18)	x	x	5.57 (0.52-59.02)
**Water supply**									
Public network	1 (base)	1 (base)	1 (base)	1 (base)	1 (base)	1 (base)	1 (base)	1 (base)	1 (base)
Others[d]	1.06 (0.95-1.17)	1.20 (0.97-1.50)	0.80 (0.49-1.31)	0.99 (0.94-1.05)	1.04 (0.95-1.14)	1.08 (0.90-1.31)	1.14 (0.80-1.61)	1.38 (0.85-2.24)	3.15 (0.71-13.8)
**Housing material (Brick)**									
Yes	1 (base)	1 (base)	1 (base)	1 (base)	1 (base)	1 (base)	1 (base)	1 (base)	1 (base)
No[e]	1.23*** (1.11-1.36)	1.39** (1.11-1.73)	1.02 (0.63-1.66)	1.25*** (1.18-1.31)	1.34*** (1.24-1.46)	1.20* (1.01-1.43)	1.17 (0.85-1.61)	1.29 (0.83-2.00)	0.26 (0.04-1.61)
**Lighting**									
Electricity	1 (base)	1 (base)	1 (base)	1 (base)	1 (base)	1 (base)	1 (base)	1 (base)	1 (base)
Non-electric[f]	1.19** (1.07-1.33)	1.28* (1.02-1.59)	1.14 (0.63-1.66)	1.33*** (1.26-1.41)	1.37*** (1.25-1.49)	1.21* (1.01-1.45)	1.34 (0.87-2.05)	1.59 (0.92-2.75)	1.54 (0.19-12.26)
**Region**									
North	1 (base)	1 (base)	1 (base)	1 (base)	1 (base)	1 (base)	1 (base)	1 (base)	1 (base)
Northeast	1.12 (0.98-1.28)	0.86 (0.65-1.14)	0.62 (0.31-1.23)	1.23*** (1.14-1.32)	1.11 (0.98-1.26)	1.18 (0.88-1.57)	0.81 (0.50-1.31)	0.66 (0.32-1.34)	0.06* (0.00-0.53)
Southeast	0.96 (0.82-1.11)	0.94 (0.69-1.29)	0.95 (0.47-1.94)	1.27*** (1.18-1.38)	1.51*** (1.33-1.71)	1.17 (0.86-1.60)	1.13 (0.72-1.77)	1.23 (0.65-2.31)	0.36 (0.04-3.07)
South	1.11 (0.92-1.32)	0.70 (0.46-1.07)	0.80 (0.34-1.91)	1.65*** (1.51-1.81)	1.61*** (1.38-1.88)	0.99 (0.71-1.38)	1.53 (0.92-2.55)	1.26 (0.59-2.70)	0.11 (0.00-2.91)

Central-west	1.05 (0.86-1.28)	0.56* (0.340.92)	0.49 (0.14-1.66)	1.28*** (1.16-1.42)	1.35*** (1.14-1.59)	1.33 (0.92-1.92)	1.62 (0.97-2.71)	1.51 (0.74-3.10)	0.12 (0.00-1.73)
**Area of residence**									
Rural	1 (base)	1 (base)	1 (base)	1 (base)	1 (base)	1 (base)	1 (base)	1 (base)	1 (base)
Urban	1.84*** (1.59-2.12)	2.23*** (1.65-2.32)	1.13 (0.31-4.07)	1.89*** (1.75-2.05)	2.24*** (1.97-2.54)	1.05 (0.77-1.43)	3.60*** (2.04-6.35)	2.58* (1.25-5.31)	0.11 (0.00-1.84)
**Average AIDS incidence rate[g]**	1.03*** (1.02-1.03)	x	x	1.03*** (1.02-1.03)	x	x	1.03*** (1.02-1.03)	x	x
**Average AIDS mortality rate[g]**	x	1.10*** (1.08-1.12)	x	x	1.09*** (1.09-1.10)	x	x	1.10*** (1.07-1.13)	x
**Average AIDS case-fatality rate[g]**	x	x	1.03*** (1.00-1.05)	x	x	1.01*** (1.00-1.02)	x	x	0.96 (0.83-1.11)
**Inadequate sanitation [h]**	0.99 (0.99-1.00)	0.99 (0.98-1.00)	0.97 (0.95-1.00)	0.99*** (0.99-0.99)	0.99** (0.98-0.99)	1.00 (0.99-1.01)	0.99 (0.97-1.01)	0.98 (0.95-1.00)	1.00 (0.89-1.14)
**Unemployment rate (%)[i]**	1.01 (1.00-1.02)	1.02 (0.99-1.04)	1.02 (0.93-1.11)	1.01*** (1.01-1.02)	1.02*** (1.01-1.03)	0.98 (0.95-1.01)	1.01 (0.97-1.06)	1.03 (0.97-1.09)	0.79 (0.51-1.22)
**Doctors per 1000 inhabitants[j]**	1.01 (0.94-1.08)	1.00 (0.86-1.17)	0.78 (0.41-1.51)	1.02 (0.99-1.05)	1.05* (1.00-1.10)	0.86* (0.77-0.97)	1.23* (1.04-1.45)	1.19 (0.96-1.48)	0.35 (0.11-1.16)
**Nurses per 1000 inhabitants[j]**	0.76* (0.62-0.94)	0.87 (0.55-1.36)	2.63 (0.54-12.8)	0.87* (0.79-0.96)	0.79* (0.68-0.93)	1.56* (1.10-2.21)	0.57* (0.34-0.96)	0.61 (0.32-1.17)	2.73 (0.09-81.86)
**Hospital beds per 1000 inhabitants[j]**	1.00 (0.98-1.03)	1.00 (0.96-1.05)	0.97 (0.81-1.16)	0.99 (0.98-1.00)	0.99 (0.97-1.01)	0.96 (0.91-1.01)	0.98 (0.90-1.05)	0.99 (0.91-1.09)	0.99 (0.40-2.41)
**Year of entry into the cohort**	yes	yes	yes	yes	yes	yes	yes	yes	yes
Obs.:	5,258,575	5,258,576	1,805	12,405,560	12,405,579	7,973	1,913,494	1,913,494	154

Notes: *** p-value <0,001; **p-valor <0,01; *p-valor <0,05. [a] Incidence-Rate Ratios. [b] Confidence Interval. [c] Measured by capita expenses proportional to the baseline minimum wage (MW). Level 1 (More wealth): "1 or more". Level 2: "0.5 to 1". Level 3: "0.25 to 0.49". Level 4: "0< to 0.24". Level 5 (Lower wealth): "Nothing declared". [d] Water supply: Others – well, spring, and others. [e] Housing Material: No – Coated clay, uncoated clay, wood, and others. [f] Lighting: Non-electric – No meter, lamp, candle and others. [g] Average rates for the period (2007-2015) by municipality. [h] % of the municipality's population with inadequate baseline sanitation. [i] Baseline municipality unemployment rate. [j] Per 1,000 inhabitants of the baseline municipality. All statistical tests used where two-sided and, where appropriate, adjustments were made for multiple comparisons.

REFERENCES

1 Sanni Ali M, Ichihara MY, Lopes LC, *et al.* Administrative data linkage in Brazil: Potentials for health technology assessment. *Front Pharmacol* 2019; **10**: 1–20.

2 Barreto ML, Ichihara MY, Pescarini JM, *et al.* Cohort profile: The 100 Million Brazilian Cohort. 2021; : 1–12.

3 DATASUS. Ministério da Saúde. https://datasus.saude.gov.br/ (accessed Dec 20, 2021).

4 Pita R, Pinto C, Sena S, *et al.* On the Accuracy and Scalability of Probabilistic Data Linkage over the Brazilian 114 Million Cohort. *IEEE J Biomed Health Inform* 2018; **22**: 346–53.

5 Pinto C, Pita R, Barbosa G, *et al.* Probabilistic Integration of Large Brazilian Socioeconomic and Clinical Databases. *Proc IEEE Symp Comput Based Med Syst* 2017; **2017-June**: 515–20.

6 Barreto, Marcos1, Alves, André, Sena, Samila, Fiaccone, Rosemeire, Amorim, Leila, Ichihara, Maria Yuri, and Barreto M. Assessing the accuracy of probabilistic record linkage of social and health databases in the 100 million Brazilian cohort. *Int J Popul Data Sci* 2017; **0**: 23889.

7 Pita R, Pinto C, Barreto M, *et al.* Design and evaluation of probabilistic record linkage methods supporting the Brazilian 100-million cohort initiative. *Int J Popul Data Sci* 2017; **1**: 23889.

8 Bolsa Família — MINISTÉRIO DA CIDADANIA Secretaria Especial do Desenvolvimento Social. http://mds.gov.br/assuntos/bolsa-familia (accessed Jan 14, 2022).

9 Lindert K, Linder A, Hobbs J, de la Brière B. The Nuts and Bolts of Brazil's Bolsa Família Program: Implementing Conditional Cash Transfers in a Decentralized Context. *Social Protection Discussion Paper Series* 2007; : 144.

10 Lawlor DA, Tilling K, Smith GD. Triangulation in aetiological epidemiology. *Int J Epidemiol* 2016; **45**: 1866–86.
