# Supplementary material for: Income determines the impact of cash transfers on HIV/AIDS: cohort study of 22.7 million Brazilians
Source: Nat Commun. 2024 Feb 12;15:1307. doi: 10.1038/s41467-024-44975-z (PMC10861499; doi:10.1038/s41467-024-44975-z)
Supplement: Supplementary file 3 — Reporting Summary [file 41467_2024_44975_MOESM3_ESM.pdf]

Corresponding author(s): Davide Rasella
Last updated by author(s): Dec 21, 2023

Reporting Summary

Nature Portfolio wishes to improve the reproducibility of the work that we publish. This form provides structure for consistency and transparency in reporting. For further information on Nature Portfolio policies, see our Editorial Policies and the Editorial Policy Checklist.

Statistics

For all statistical analyses, confirm that the following items are present in the figure legend, table legend, main text, or Methods section.

n/a	Confirmed	
☐	☒	The exact sample size (*n*) for each experimental group/condition, given as a discrete number and unit of measurement
☐	☒	A statement on whether measurements were taken from distinct samples or whether the same sample was measured repeatedly
☐	☒	The statistical test(s) used AND whether they are one- or two-sided<br>*Only common tests should be described solely by name; describe more complex techniques in the Methods section.*
☐	☒	A description of all covariates tested
☐	☒	A description of any assumptions or corrections, such as tests of normality and adjustment for multiple comparisons
☐	☒	A full description of the statistical parameters including central tendency (e.g. means) or other basic estimates (e.g. regression coefficient) AND variation (e.g. standard deviation) or associated estimates of uncertainty (e.g. confidence intervals)
☐	☒	For null hypothesis testing, the test statistic (e.g. *F*, *t*, *r*) with confidence intervals, effect sizes, degrees of freedom and *P* value noted<br>*Give P values as exact values whenever suitable.*
☒	☐	For Bayesian analysis, information on the choice of priors and Markov chain Monte Carlo settings
☒	☐	For hierarchical and complex designs, identification of the appropriate level for tests and full reporting of outcomes
☐	☒	Estimates of effect sizes (e.g. Cohen's *d*, Pearson's *r*), indicating how they were calculated

Our web collection on statistics for biologists contains articles on many of the points above.

Software and code

Policy information about availability of computer code

Data collection	100 Million Brazilian Cohort [Three individual-level related datasets were linked: the Unified Registry for Social Programs: the Notifiable Diseases Information System (SINAN) and the Mortality Information System (SIM)].
Data analysis	Statistical software: Stata 15

For manuscripts utilizing custom algorithms or software that are central to the research but not yet described in published literature, software must be made available to editors and reviewers. We strongly encourage code deposition in a community repository (e.g. GitHub). See the Nature Portfolio guidelines for submitting code & software for further information.

Data

Policy information about availability of data

All manuscripts must include a data availability statement. This statement should provide the following information, where applicable:

- Accession codes, unique identifiers, or web links for publicly available datasets
- A description of any restrictions on data availability
- For clinical datasets or third party data, please ensure that the statement adheres to our policy

DATA AVAILABILITY
The protocol for the creation of the 100 Million Brazilians Cohort and the cohort profile of the 100 Million Brazilians Cohort is available in the publications referenced in the article and further material is available at: https://cdidacs.bahia.fiocruz.br/en/platform/cohort-of-100-millionbrazilians.

The linkage protocols are explained in the referenced publications and the codes are available at: https://gitHub.com/gcgbarbosa/cidacs-rl.
However, the datasets generated during and analyzed during the current study are not publicly available due to confidentiality and ethical issues. To request access, contact us at: https://cidacs.bahia.fiocruz.br/contato/fale-conosco/.
CODE AVAILABILITY
The code can only be shared on request due to confidentiality and ethical issues.

Research involving human participants, their data, or biological material

Policy information about studies with human participants or human data. See also policy information about sex, gender (identity/presentation), and sexual orientation and race, ethnicity and racism.

Reporting on sex and gender	The findings apply to only on sex. This information has been collected, and consent has been obtained for sharing of individual-level data; Overall numbers: 22,788,998, 57,99% (13,214,290) are female and 42,01% (9,574,707) are male.
Reporting on race, ethnicity, or other socially relevant groupings	Were definited how White, Mixed-race, Black and Indigenous. They were provided by Unified Registry for Social Programs. The method used to classify people into the different categories was self-report.
Population characteristics	This study has a quasi-experimental cohort study design, based on the longitudinal information of 22.7 million individuals aged 13 and older, from January 1 2007 to December 31, 2015. At the individual level, the demographic and socioeconomic and variables included in the models were sex, race, age, educational achievement, per capita expenditures used as a proxy for wealth, household characteristics (adequate water supply, household construction material, and installed electric power), geographic region, area of residence (urban and rural), and year of entry into the cohort. When the study outcome was the case fatality rate, we also included compliance with antiretroviral treatment (ART) as an independent variable. As municipal-level variables, we included the proportion of the population with inadequate sanitation, the unemployment rate, and a set of healthcare service-related variables (calculated as a rate per 1,000 inhabitants in the municipality): the number of doctors, nurses, and hospital beds.
Recruitment	The CadUnico is an administrative database, to which Brazilians aged 16 or over can apply who sought to benefit from the Brazilian government's social programs [incluided Programa Bolsa Familia (PBF)] . To address any potential limitations in our study design, selection bias, it is crucial to clarify that the assumption of the PBF rules selecting the more "motivated" individuals and leaving the vulnerable population in the "non-benefit" comparator group does not apply. This is because the participants of this study are only individuals registered in the Unified Registry, and after this registration process, the centralized system of the Ministry of Social Development enrolls them into the PBF based on specific eligibility rules with no influence during this enrollment phase of any condition or CCT conditionality.
Ethics oversight	This study was approved by the Research Ethics Committee of the Institute of Collective Health of the Federal University of Bahia (ISC/UFBA), under number 41691315.0.0000.5030 (Report No:3.783.920).

Note that full information on the approval of the study protocol must also be provided in the manuscript.

Field-specific reporting

Please select the one below that is the best fit for your research. If you are not sure, read the appropriate sections before making your selection.

☐ Life sciences ☒ Behavioural & social sciences ☐ Ecological, evolutionary & environmental sciences

For a reference copy of the document with all sections, see nature.com/documents/nr-reporting-summary-flat.pdf

Behavioural & social sciences study design

All studies must disclose on these points even when the disclosure is negative.

Study description	Type quantitative: study of the Cohort. This study has a quasi-experimental cohort study design, based on the longitudinal information of 22.7 million individuals aged 13 and older, from January 1 2007 to December 31, 2015.
Research sample	Dataset and source: 100 Million Brazilian Cohort [Three individual-level related datasets were linked: the Unified Registry for Social Programs: the Notifiable Diseases Information System (SINAN) and the Mortality Information System (SIM)]. Unified Registry had approximately 114 million individuals on its register, which represents around 50% of the Brazilian population. It is a social tool that identifies and characterizes especially low-income families, allowing the government to know the socioeconomic aspect of the poorest and use it for the selection of social programs. Therefore, the sample is representative.
Sampling strategy	The selection process to achieve our final cohort, which was composed of 22,788,998 individuals between 2007 and 2015 is because the period for which HIV/AIDS data were available. The population under study (sampling procedure) was achieved by selecting all individuals aged 13 and older from the 100 Million Brazilian Cohort,20 a consolidated cohort created through the linkage between the Federal Government Unified Registry for Social Programs (Cadastro Único) – that gathers data from the lower-income half of the Brazilian population, identifying and characterizing low-income families for social programs eligibility - and health-related datasets from the Brazilian Ministry of Health's
Data collection	The data collection and treatement procedure was computer and Stata software. Upon registration in Unified Registry for Social

Data collection	Programs, individuals receive a unique identifier code.<br>The researcher was a blind to experimental condition and the study hypothesis during data collection.
Timing	From January 1 2007 to December 31, 2015
Data exclusions	Data were excluded (is described in Figure 1): Individuals in the 100 Million Brazilian Cohort baseline (2001-2015) (n=114,028,659) - Excluded (n=20,354) Duplicate cleaning;<br>Excluded (n=60,998,196) Individuals who entered the cohort between 2001 and 2006;<br>Excluded (n= 24,691,578: 28,627 AIDS cases and 3,337 AIDS-related deaths):<br>-24,606,051 individuals under the age of 13 (763 AIDS cases and 276 AIDS-related deaths)<br>-50,013 individuals with a date of death before their date of entry into the cohort (799 AIDS cases and 748 AIDS-related deaths)<br>-35,119 individuals with AIDS cases, with a diagnosis date before their date of entry into the cohort (26,876 AIDS cases and 2,282 AIDS-related deaths)<br>-395 individuals with AIDS due to probable vertical transmission (189 AIDS cases and 31 AIDS-related deaths)<br>Obs.: individuals with AIDS were considered non-AIDS cases since the date of diagnosis only occurred after the cohort had ended, 2015 (3,142 AIDS cases and 35 AIDS-related deaths)<br>Excluded (n= 5,529,533: 5,707 AIDS cases and 1,880 AIDS-related deaths)<br>-Cuts with Programa Bolsa Família (PBF) variables<br>-2,778,206 individuals with a start date for BF receipt before 2007 (3,009 AIDS cases and 936 AIDS-related deaths)<br>-2,587,932 individuals with a start date for BF receipt before their date of entry into the cohort (2,552 AIDS cases and 885 AIDS-related deaths)<br>-163,395 individuals with 30 days or less of receiving BF in the cohort (146 AIDS cases and 59 AIDS-related deaths)<br>Individuals in the cohort (2007-2015) (n= 22,788,998) (22,212 AIDS cases and 7,650 AIDS-related deaths).
Non-participation	No participants dropped out/declined participation
Randomization	The allocation was not random, participants were allocated to groups how: the beneficiary group was defined as eligible individuals who received PBF benefits (extremely poor families and poor families), and their exposure started with the receipt of the benefit, until the end of their cohort follow-up. The non-beneficiary group was defined as individuals who had never benefited from PBF throughout their follow-up period. In case of administrative delays and non-receipt of the benefits, eligible individuals were classified in the non-beneficiary group.

Reporting for specific materials, systems and methods

We require information from authors about some types of materials, experimental systems and methods used in many studies. Here, indicate whether each material, system or method listed is relevant to your study. If you are not sure if a list item applies to your research, read the appropriate section before selecting a response.

Materials & experimental systems

n/a	Involved in the study	
☒	☐	Antibodies
☒	☐	Eukaryotic cell lines
☒	☐	Palaeontology and archaeology
☒	☐	Animals and other organisms
☒	☐	Clinical data
☒	☐	Dual use research of concern
☒	☐	Plants

Methods

n/a	Involved in the study	
☒	☐	ChIP-seq
☒	☐	Flow cytometry
☒	☐	MRI-based neuroimaging
